# Human-specific morphoregulatory signatures in basal radial glia characterise neocortex evolution

**DOI:** 10.1038/s41467-026-78474-0

**Published:** 2026-10-09

**Authors:** Theresa M. Schütze, Annika Kolodziejczyk, Nora Ditzer, Silvia Vangelisti, Emanuele Capra, Jeong-Eun Lee, Ilaria Chiaradia, Vida Kufrin, Seiya Yamada, Jula Peters, Maximilian Krause, Christina Eugster Oegema, Razvan P. Derihaci, Cahit Birdir, Takashi Namba, Alexander A. Wurm, Ulrich Martin, Pauline Wimberger, Katherine R. Long, Madeline A. Lancaster, Nereo Kalebic, Boyan Bonev, Mareike Albert

**Affiliations:** 1 Center for Regenerative Therapies TU Dresden, TUD Dresden University of Technology, Dresden, Germany; 2 Research Unit Brain Epigenomics, Helmholtz Center Munich, Munich, Germany; 3 Human Technopole, Milan, Italy; 4 MRC Laboratory of Molecular Biology, Cambridge Biomedical Campus, Cambridge, UK; 5 Department of Pediatric Hematology and Oncology, University Hospital Dresden, Dresden, Germany; 6 Department of Translational Medical Oncology, National Center for Tumor Diseases Dresden, a partnership between DKFZ, Faculty of Medicine of the Technische Universität Dresden, University Hospital Carl Gustav Carus Dresden, and Helmholtz-Zentrum Dresden - Rossendorf, Dresden, Germany; 7 Neuroscience Center, HiLIFE - Helsinki Institute of Life Science, University of Helsinki, Helsinki, Finland; 8 Max Planck Institute of Molecular Cell Biology and Genetics, Dresden, Germany; 9 DRESDEN-concept Genome Center, Center for Molecular and Cellular Bioengineering Technology Platform, TUD Dresden University of Technology, Dresden, Germany; 10 Department of Gynaecology and Obstetrics, Technische Universität Dresden, Dresden, Germany; 11 National Center for Tumor Diseases, Dresden, Germany; 12 Department of Obstetrics and Gynecology, University Hospital of Heidelberg, Heidelberg, Germany; 13 Department of Developmental Biology, Fujita Health University School of Medicine, Toyoake, Japan; 14 Division of Developmental Neurobiology, International Center for Brain Science (ICBS), Fujita Health University, Toyoake, Japan; 15 Leibniz Research Laboratories for Biotechnology and Artificial Organs, Department of Cardiothoracic, Transplantation and Vascular Surgery, Hannover Medical School, Hannover, Germany; REBIRTH-Cluster of Excellence, Hannover, Germany; 16 Centre for Developmental Neurobiology, Institute of Psychiatry, Psychology and Neuroscience, King’s College London, London, UK; 17 MRC Centre for Neurodevelopmental Disorders, King’s College London, London, UK

**Keywords:** Neural progenitors, Evolutionary developmental biology

## Abstract

As the seat of our cognition, the human neocortex is an object of immense fascination. Human neocortex expansion during evolution has been attributed to an increase in the proliferative capacity of neural progenitor cells during development, particularly basal radial glia. Despite their evolutionary relevance, the genomic changes driving human basal radial glia biology remain uncharacterised. We use comparative chromatin and transcriptional profiling of neural progenitor cells isolated from gorilla, chimpanzee and human cerebral organoids to identify cis-regulatory elements that have gained activity in humans. Focusing specifically on basal radial glia, we discover that morphoregulatory enhancer activity and gene expression signatures distinguish human basal radial glia from other great apes. Functional analysis of the morphoregulatory genes *FAM107A* and *CNGA3* in human organoids reveals that these genes contribute to the morphological complexity of human basal radial glia. Taken together, our inter-species comparison of basal radial glia suggests that human-specific morphoregulatory signatures characterise neocortex evolution.

## Abstract (web summary)

Inter-species comparison of great ape brain development using cerebral organoids provides novel insights into neocortex evolution. This work identifies human-specific gene regulatory signatures linked to cell morphology.

## Introduction

As the seat of higher-level cognitive functions, including speech, self-reflection, and long-term planning^1,2^, the neocortex is central to the experience of being human. In the course of primate evolution, the neocortex has undergone a number of significant changes^3,4^, including a drastic increase in size and neuron number, both of which peak in humans^5^. Neocortical neurons arise embryonically from three main classes of neural progenitor cells: (i) apical radial glia (aRG) that divide in the ventricular zone of the developing cortex, (ii) basal intermediate progenitors (bIPs) without ventricular contact that divide in the subventricular zone, and (iii) basal (or outer) radial glia (bRG/oRG) that also divide in the subventricular zone and have repeatedly been implicated in the evolution of neocortex size. The abundance^6–9^ and proliferative potential^10–12^ of bRG are tightly linked to increased neuron numbers in the adult neocortex and overall neocortex size. Key factors underlying bRG function include their morphological complexity^12,13^, lineage relationships^12,14,15^ and altered cell metabolism^16^, all of which correlate with larger neocortex size across species.

The human-specific genomic changes underlying human bRG biology remain to be elucidated, promising unique insights into the mechanisms that ultimately provide the basis for higher cognitive functions. While a limited number of human-specific genes affecting bRG proliferative potential and neocortex size have been described^3,4,17^, a large part of great ape species-specific biology likely originates in the differential regulation of shared genes orchestrated by cis-regulatory elements (CREs)^18–21^. Inter-species differences in CRE activity may alter the expression of associated target genes, affecting radial glia biology, as shown for the Wnt receptor FZD8^22^, the growth factor EPIREGULIN^23^ and *HAR1984*^24^. Epigenomic comparisons of foetal brain tissue from different species have identified candidate CREs, termed human-gained enhancers (HGEs), with a potential role in neocortex evolution^25,26^. Yet, since great ape foetal tissue is not accessible for research, truly human-specific changes in bRG CRE activity have not been elucidated. Recent advances in induced pluripotent stem cells (iPSCs) from great apes have allowed epigenomic comparisons of cultured neural progenitor cells and neurons^27,28^. These comparisons, however, provide a limited reflection of in vivo development due to the inability to recapitulate tissue morphology in 2D cultures. Great ape cerebral organoid models can offer additional insights into human-specific enhancer activity^29^, in particular, as cerebral organoids contain bRG^30,31^.

In this work, using 3D organoid models, we set out to identify CRE activity patterns in human bRG that are likely to contribute to their extensive proliferative potential, neuronal output, and, ultimately, human neocortex expansion. We identify human-specific bRG signatures involved in the regulation of neuronal cell morphology, in particular, cell projections.

## Results

### Distinct molecular profiles of great ape bRG

**Fig. 1:**
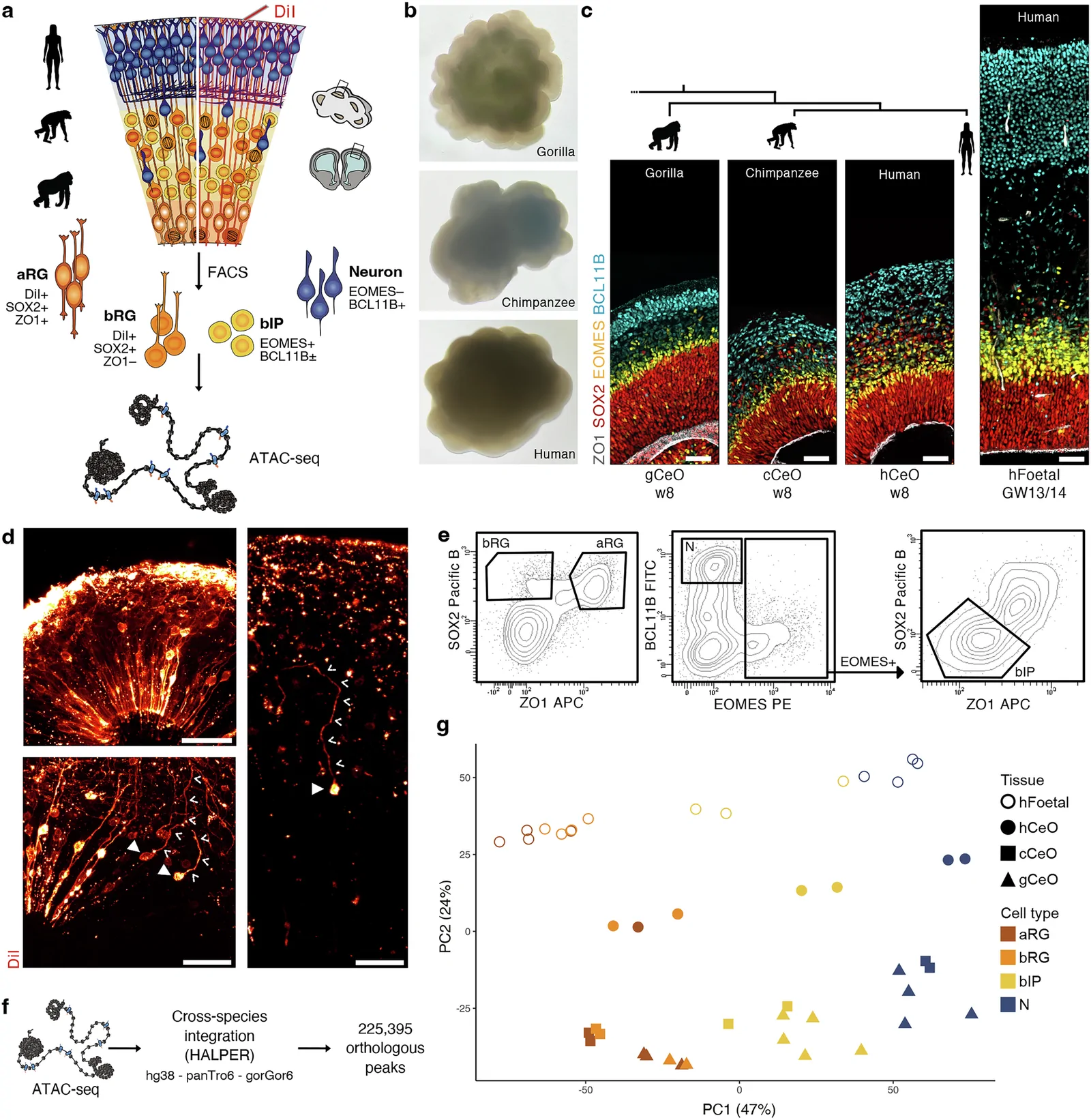
Cell type-specific ATAC-seq reveals distinct cellular profiles in great ape corticogenesis. **a** Scheme of the isolation of aRG, bRG, bIPs, and N from gorilla, chimpanzee, and human CeOs and human foetal cortex. The lipophilic dye DiI labels cells with processes reaching the basal side of the tissue, followed by tissue dissociation, immunocytochemistry and FACS. ATAC-seq is performed on all cell populations. **b** Brightfield image of a whole gorilla, chimpanzee and human CeO at w8. **c** Immunohistochemistry for ZO1, SOX2, EOMES, and BCL11B in gorilla, chimpanzee, and human CeOs and human foetal cortex. Representative images of ≥ 3 replicates. **d** Sparse DiI labelling reveals radial tissue morphology and highlights the presence of bRG cell bodies (filled arrowheads) and processes (open arrowheads). Representative images of ≥3 replicates. **e** Left: FACS plots of GW13 human foetal cortex showing separation of aRG and bRG based on expression of ZO1, whereas SOX2 is expressed in both RG populations. Both radial glia populations are further sub-gated for DiI to select cells with basal processes. Right: FACS plot of the same GW13 human foetal cortex sample, showing gating for neurons as BCL11B+/EOMES– and gating for bIPs as EOMES+/SOX2–/ZO1–. **f** ATAC-seq libraries are sequenced by next-generation sequencing, and orthologs for regions mapped to hg38, panTro6, and gorGor6 are determined using HALPER^40^. **g** Principal component analysis of the 1000 most variable regions shows a separation of cell types on PC1 (47%) and species on PC2 (24%). gCeO, cCeO, hCeO, gorilla, chimpanzee and human cerebral organoid; hFoetal, human foetal cortex. Scale bars, 50 μm.

To characterise human-specific gene-regulatory signatures, we studied chromatin accessibility and gene expression in aRG, bRG, bIPs, and neurons during great ape cortical development (Fig. 1a). We generated cerebral organoids (CeOs)^32^ from gorilla, chimpanzee, and human iPSCs (Fig. 1b), which contain SOX2+ aRG and bRG, EOMES+ bIPs and BCL11B+ neurons, comparable to human foetal brain tissue (Fig. 1c), and express the forebrain marker FOXG1 (Supplementary Fig. 1a). Application of the lipophilic dye DiI to the surface of CeOs revealed the presence of bRG with characteristic basal processes (Fig. 1d and Supplementary Fig. 1b), including morphological variety previously described in macaque and human foetal cortex^12,13^. Moreover, immunohistochemistry identified bRG labelled with classical markers (SOX2+, EOMES–, HOPX+, and pVIM+ with a basal process) in the subventricular zone (Supplementary Fig. 1c), confirming that CeOs can be used to study bRG.

We next aimed to isolate specific cell populations from CeOs derived from all three species as well as from human foetal cortex. A key consideration was the developmental stage at which to perform these analyses. We selected week 8 of CeO development and gestational weeks (GW) 13–14 of human foetal cortex, as all four cell types of interest are present at these stages (Fig. 1c). Moreover, at this developmental stage, CeO and foetal brain development show a high degree of similarity at both the cellular and transcriptomic level^33,34^, supporting their comparability. Direct cross-species alignment of developmental timing is complicated by the limited availability of foetal tissue from great apes. However, gestation length and the timing of *in utero* developmental milestones are highly temporally correlated across humans, chimpanzees, and gorillas^3,35^ (Supplementary Fig. 1d). Consistent with this, comparative CeO studies indicate that early cortical development, specifically the transition from neuroepithelial cells to aRG, is delayed in humans relative to non-human great apes^36^, whereas mid-neurogenic stages (approximately 1–3 months of organoid age) appear highly synchronised across species^29,37,38^ (Supplementary Fig. 1e). We further validated this equivalency in our system by performing a time-course analysis of CeO development by immunohistochemistry across species (Supplementary Fig. 1f).

Having established the timing, we isolated specific cell populations (Fig. 1e and Supplementary Fig. 2a‒c), based on the presence/absence of ventricular contact, basal processes, and marker protein expression^10,39^. RT-qPCR was used to confirm the identity of sorted cell populations (Supplementary Fig. 2d‒f). We assayed open chromatin using ATAC-seq and integrated all great ape data sets using HALPER^40^, yielding 225,395 orthologous peaks (Fig. 1f). We observed expected patterns of cell type-specific accessibility at marker genes and clustering of cell types (Supplementary Fig. 3). Principal component analysis revealed a separation of cell types on PC1, accounting for 47% of the variation in the data, with a trajectory from radial glia to bIPs to neurons, demonstrating that broad cellular signatures were conserved across hominids (Fig. 1g). We confirmed that these patterns are not dependent on cell line or organoid protocol by isolating cells from sliced cortical organoids^31^ (COs) of two additional human cell lines (Supplementary Fig. 4). In line with previous literature^14,41–44^, aRG and bRG were not distinctly separated at this developmental stage and molecular level (Fig. 1g and Supplementary Fig. 3b and 4f); rather bRG showed a pattern of shared accessibility features with the other neural cell types (Supplementary Fig. 3c). PC2 accounted for 24% of the variation and separated the species, revealing divergent species-specific molecular profiles of the different cell types, including human-specific features of bRG (Fig. 1g and Supplementary Fig. 4e).

### Human bRG chromatin accessibility links to morphoregulatory genes

**Fig. 2:**
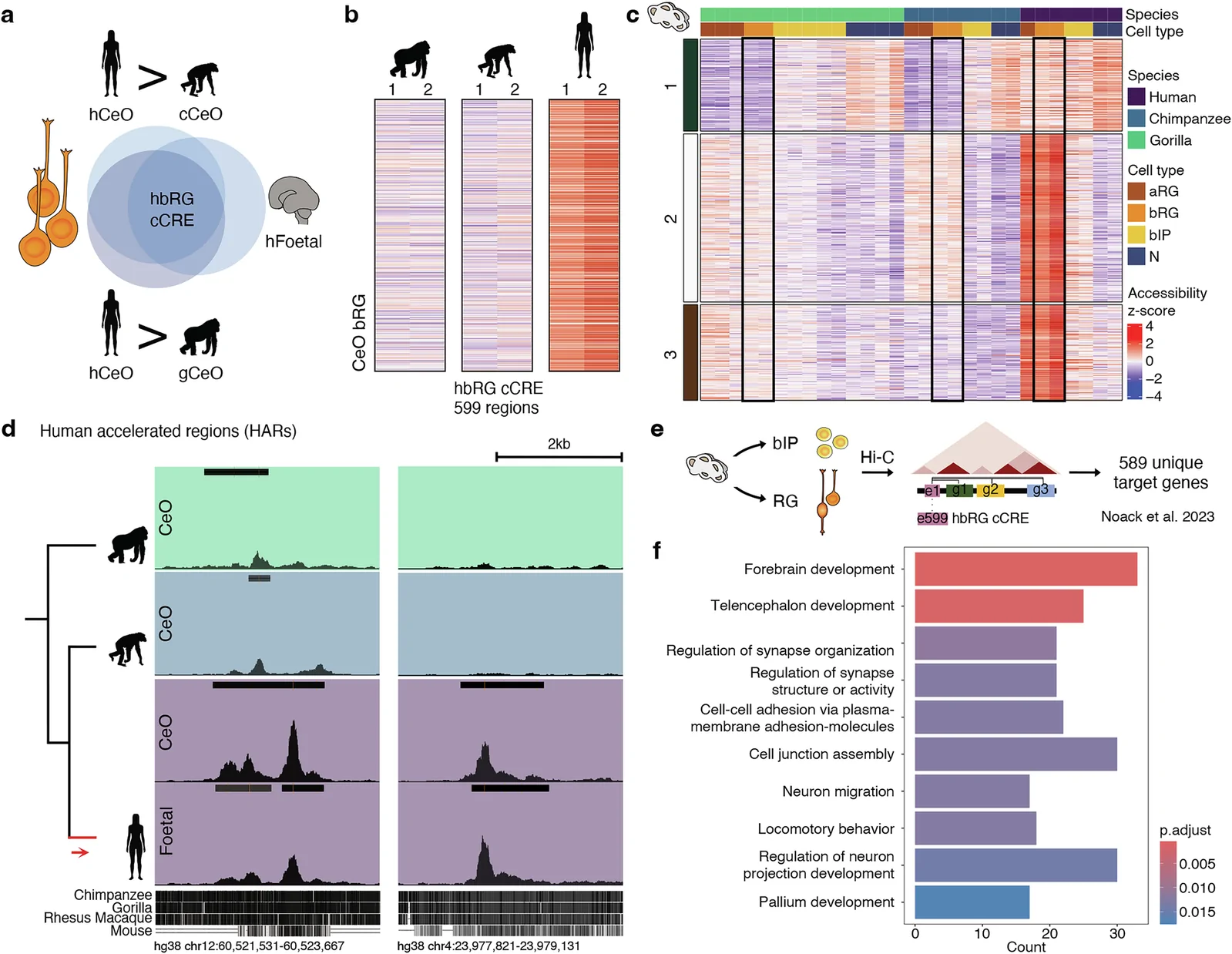
Human-specific patterns of chromatin accessibility in bRG link to cell morphology genes. **a** Human-increased chromatin accessibility in bRG candidate cis-regulatory elements (hbRG cCRE) is identified by overlapping regions found to be significantly more accessible (log2FC ≥ 1.5; *p* ≤ 0.05) in human bRG than in both chimpanzee and gorilla. Regions are benchmarked against peaks in human foetal bRG. **b** Accessibility z-scores of the 599 hbRG cCRE from (a) in the two replicates of gorilla, chimpanzee and human CeO bRG. **c** K-means clustering of the 599 hbRG cCRE reveals three broad patterns of accessibility across all CeO samples and cell types. Black boxes highlight gorilla, chimpanzee and human bRG. **d** Two examples of human accelerated regions that are found among the hbRG cCRE. Shown are merged bigWig tracks of at least two replicates, with standardised track height 0–3. Called peaks are represented by black bars in tracks. Cactus evolutionary sequence conservation and chromosome coordinates are indicated. **e** Candidate CREs were linked to target genes using cell type-specific Hi-C data from radial glia and bIPs^49^, resulting in 589 unique target genes in physical contact with the 599 hbRG cCREs. **f** GO term analysis of target genes indicates enrichment of genes linked to forebrain development, regulation of cell-cell contacts and regulation of mature neural cell morphology. GO term enrichment was assessed using a hypergeometric test, with *P*-values adjusted for multiple comparisons using the Benjamini–Hochberg method. GO terms with adjusted *p* < 0.05 and *q* < 0.1 were considered significant. Top 10 terms displayed with *p* ≤ 0.05.

Given the evolutionary relevance of bRG, we next aimed to define species-specific patterns of bRG chromatin accessibility by multivariate analysis. We therefore identified differentially accessible distal chromatin peaks in human compared to chimpanzee and gorilla bRG isolated from CeOs (log2FC ≥ 1.5; *p* ≤ 0.05). To ensure in vivo developmental relevance, we further cross-referenced these peaks with regions accessible in bRG that we isolated from human foetal cortex (Fig. 2a). This analysis led to the identification of 599 candidate CREs with higher accessibility in human bRG, which we termed human bRG candidate CREs (hbRG cCREs) (Fig. 2a, b and Supplementary Data 1). These hbRG cCREs fell into three broad clusters of shared accessibility with other cell types. Cluster 1 showed ancestral accessibility in bIPs and neurons, gained in human aRG and bRG. Clusters 2 and 3 showed strongly increased accessibility in human aRG and bRG, shared to a lesser degree with radial glia of both other great apes (cluster 2) or only with gorilla (cluster 3) (Fig. 2c). Interestingly, clusters 2 and 3 also showed some accessibility in human bIPs, indicating they may be more broadly relevant to human neural progenitor cell biology.

The accessibility of the 599 candidate CREs was consistent across human CO from the additional two human cell lines (Supplementary Fig. 4g, h). Moreover, published scATAC-seq peak sets of CeOs (age 60‒120 days) from six human and three chimpanzee cell lines^29^ showed substantial overlap with our data (Supplementary Fig. 5). Together, these comparisons indicate strong concordance between datasets and suggest that our findings generalise across independent human and chimpanzee cell lines despite differences in organoid age and experimental platforms.

Characterising hbRG cCREs further, we found that 63% overlap with CRE signatures defined by the ENCODE Project^45^ and three regions overlapped with the VISTA enhancer database^46^ (Supplementary Fig. 4i). Human accelerated regions, repeatedly indicated to harbour significant gene-regulatory potential relevant to human brain evolution^22,24,47,48^, were represented among the hbRG cCREs (Fig. 2d and Supplementary Fig. 4j) (genome-wide enrichment tested by Fisher’s exact test; *p* = 0.0509; odds ratio = 2.3), adding support to the notion that human accelerated regions are highly relevant in the context of human-specific neurodevelopmental gene regulation.

To explore the potential biological role of hbRG cCRE activity, we leveraged cell type-specific Hi-C data of cortical organoids^49^ to link our candidate regions to their target genes. We found that the 599 regions target a total of 589 unique genes (Fig. 2e). GO term enrichment analysis revealed that the gene set is associated with a broad range of neural developmental processes, including forebrain and telencephalon development, synapse organisation and activity, cell-cell adhesion and cell junction assembly, neuronal migration, and locomotory behaviour (Fig. 2f). Many of these processes, such as synapse formation, cell junction assembly and neuron projection development, are inherently morphoregulatory, as they involve structural organisation and remodelling at the cellular and tissue levels. This suggests that the gene set contributes to both the function and morphology of neural cells, often in the context of mature neural cells, rather than progenitors. We thus hypothesise that human bRG employ both ancestral neuronal CREs (Fig. 2c; cluster 1) as well as increased activity of ancestral radial glia CREs (Fig. 2c; clusters 2 and 3) to regulate genes classically associated with controlling morphoregulatory processes in mature neural cells such as neurons.

### Human bRG express factors associated with cell projections

**Fig. 3:**
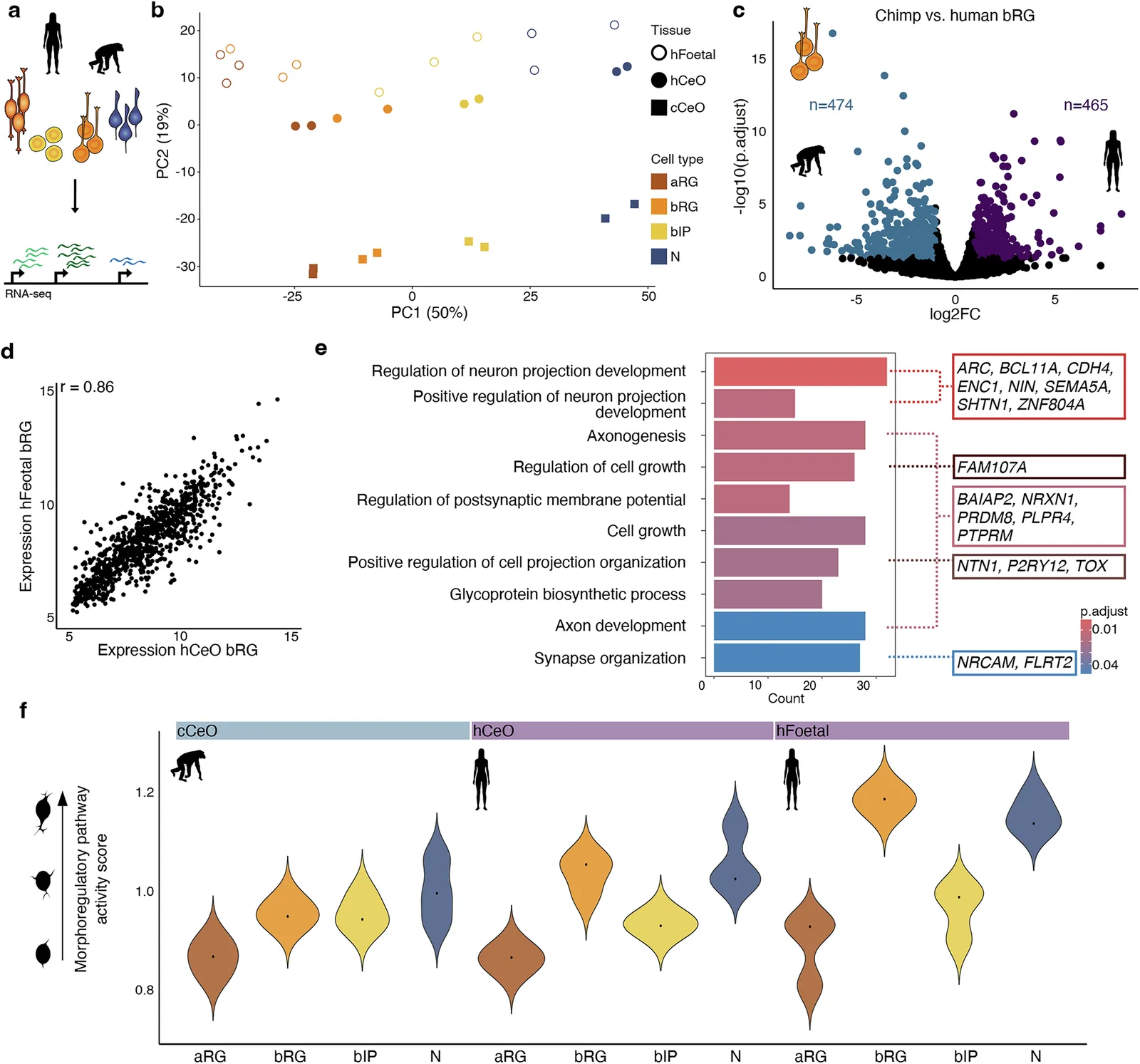
Genes more highly expressed in human than chimpanzee bRG indicate regulation of morphoregulatory pathways. **a** RNA-seq is performed on the four sorted cell types from chimpanzee and human CeOs and human foetal cortex. **b** Principal Component analysis of 500 most variable genes shows a separation of cell types on PC1 (50%) and species on PC2 (19%). **c** Volcano plot showing genes significantly upregulated in chimpanzee (blue) and human (purple) bRG (adjusted *p* ≤ 0.05; log2FC ≥ 1). Differential expression was assessed using the two-sided Wald test implemented in DESeq2. *P*-values were adjusted for multiple comparisons using the Benjamini–Hochberg method. **d** Correlation of variance-stabilised expression of bRG DEGs in human CeO and foetal bRG. **e** GO term analysis of human bRG upregulated genes indicates enrichment of genes linked to the regulation of cell morphology. GO term enrichment was assessed using a hypergeometric test, with *P*-values adjusted for multiple comparisons using the Benjamini–Hochberg method. GO terms with adjusted *p* < 0.05 and *q* < 0.1 were considered significant. All detected genes were used as background. Top 10 terms displayed with *p* ≤ 0.05. Example genes are indicated on the right. **f** Morphoregulatory pathway activity score, adapted from^53^ and calculated based on GO term gene groups ‘Cell morphogenesis’, ‘Cell projection morphogenesis’ and ‘Cell projection organisation’, indicates higher morphoregulatory activity in human bRG compared to chimpanzee bRG. Source data are provided as a Source Data file.

To further investigate the possibility of altered morphoregulatory dynamics in human bRG, we generated RNA-seq data from chimpanzee and human CeO and foetal tissue (Fig. 3a). We evaluated two mapping strategies to account for potential biases arising from differences in genome assemblies and annotations. First, all datasets were mapped to the human reference genome (hg38), which yielded clear separation of cell types and high concordance between biological replicates (Supplementary Fig. 6a). Second, reads were aligned to species-specific genome assemblies, with human gene annotations projected using the Comparative Annotation Toolkit (CAT) and filtered with CrossFilt^50^ to reduce biases from non-reciprocal mappings. Results were highly concordant between approaches (Supplementary Fig. 6b‒d). However, mapping to hg38 enabled the detection of additional genes of interest (e.g., *HES5*) that are not reliably represented in current non-human primate assemblies. We therefore used hg38-based mapping for all downstream analyses.

Principal component analysis (Fig. 3b) and gene expression correlation analysis (Supplementary Fig. 6a) mirrored cell type- and species-specific patterns observed at the chromatin level. Marker gene analysis confirmed both regional and cell type identity at the transcriptomic level (Supplementary Fig. 6e, f). We found a total of 939 differentially expressed genes (log2FC ≥ 1; adjusted *p* ≤ 0.05) between chimpanzee and human bRG (Fig. 3c and Supplementary Data 2, 3), whose expression pattern in hCeO was highly correlated to that of hFoetal bRG (Fig. 3d). GO term analysis refined the pattern observed in hbRG cCRE target genes, with genes upregulated in human bRG predominantly enriched in processes regulating neural cell morphology (Fig. 3e). Of note, GO terms classifying processes such as the ‘Regulation of neuron or cell projections’ and ‘Axonogenesis’ were specific to genes upregulated in human bRG, and not detected for aRG and bIPs (Supplementary Fig. 6g, h). This suggests that altered morphoregulatory dynamics of cell projections are a human bRG-specific phenotype. Indeed, *PALMD*, previously identified as a crucial regulator of basal progenitor morphological complexity and proliferative potential in the context of neocortex evolution^13^, was significantly upregulated in human bRG compared to chimpanzee bRG, but not in human aRG or bIPs (Supplementary Fig. 6i).

To further validate human bRG upregulated genes, we examined their expression patterns in cell types isolated from human CO derived from two additional human cell lines as well as from gorilla CeOs (Supplementary Fig. 7a‒d and Supplementary Data 3). Expression patterns across human cell lines were highly consistent (Supplementary Fig. 7e‒g). Moreover, the genes upregulated in human compared to chimpanzee bRG showed broadly lower expression in gorilla compared to human bRG (Supplementary Fig. 7h, i), suggesting human up- rather than chimpanzee downregulation. Conversely, genes upregulated in chimpanzee bRG were generally more highly expressed in gorilla than in human bRG (Supplementary Fig. 7h, i). In addition, many of the morphoregulatory genes upregulated in human compared to chimpanzee bRG (Fig. 3e) were also expressed at lower levels in gorilla bRG (Supplementary Fig. 7j and Supplementary Data 2, 3) and chimpanzee bRG of published scRNA-seq data^29,51,52^ (Supplementary Fig. 8a; *n* = 186 human and chimpanzee CeO bRG cells). Finally, the generalizability of patterns across datasets and organoid stages was further supported by pan-radial glia differentially expressed genes identified in our data, showing broadly consistent species-specific expression patterns in previously published scRNA-seq data^29^ (Supplementary Fig. 8b‒h).

Given that both hbRG cCRE target genes and human bRG upregulated genes indicated differential regulation of cell morphology, we explored this aspect in more detail. Leveraging previously established approaches to determine a glycolysis score^34^ or metabolic pathway activity^53^, we calculated a ‘Morphoregulatory pathway activity score’, using the gene sets contained within the GO-Terms ‘Cell morphogenesis’, ‘Cell projection morphogenesis’ and ‘Cell projection organisation’ (Fig. 3f; see Methods for details). In short, for each pathway, gene expression values were normalised by the mean expression of each gene across all samples and summarised as a weighted average, giving lower weight to genes present in multiple pathways. To validate the score in a cortical context, we applied the same framework to GO terms associated with apical polarity (‘Apical junction assembly’, ‘Establishment of epithelial cell apical basal polarity’ and ‘Regulation of adherens junction organization’), which resulted in the expected high pathway activity in aRG compared to bRG, bIPs and neurons (Supplementary Fig. 9a). Furthermore, in a published dataset of astrocyte maturation in human organoids^54^, the morphoregulatory pathway activity score increased with cellular maturation and complexity (Supplementary Fig. 9b).

In our chimpanzee CeOs, the morphoregulatory pathway activity score increased from aRG to bRG and bIPs and then again to neurons, in line with the biological progression from a bipolar cell (aRG) to what in primates often are morphologically more complex basal progenitors^12,13^ to, finally, highly complex neurons (Fig. 3f). In humans, the pathway activity mirrored the general increase from aRG to basal progenitors to neurons. Of note, human bRG showed a score almost identical to neurons, outstripping both human aRG and bIPs as well as chimpanzee neural progenitor cells (Fig. 3f). These results further underline the concept that altered morphoregulatory signatures are a key feature of human bRG biology.

### Human bRG show signatures of co-option from neurons

As many of the genes contributing to the GO-Term enrichments and morphoregulatory pathway score are classically associated with neuronal morphogenesis, this raises the possibility that aspects of (ancestral) neuronal gene expression programs may be active in human bRG. To explore this possibility, we examined human bRG-upregulated genes in published data sets of different developmental stages of human foetal, macaque foetal and mouse embryonic cortex (Supplementary Fig. 10, 11).

The 465 human bRG-upregulated genes (Fig. 3c) segregated into two broad classes in human foetal cell types: those more highly expressed in bIPs and neurons (Supplementary Fig. 10a; cluster 1) and those with relatively higher expression in radial glia (Supplementary Fig. 10a; cluster 2). Projection of the 465 human bRG-upregulated genes onto UMAP embeddings across developmental stages (GW11–18) in human foetal snRNA-seq datasets^55^ confirmed consistent expression in radial glia as well as excitatory and inhibitory neurons (Supplementary Fig. 10b‒d). This pattern was further supported by cross-species analyses. In the macaque foetal cortex^56^, the same gene set was primarily expressed in neural progenitor cells and excitatory neurons (Supplementary Fig. 10e), with enrichment in bRG-like populations (Supplementary Fig. 10f). Notably, a subset of morphoregulatory candidate genes, including *BCL11A*, *ENC1*, *BAIAP2*, and *PTPRM*, showed strong expression in human CeO and foetal bRG (Supplementary Fig. 8a, 11a) but low or absent expression in macaque bRG (Supplementary Fig. 11b, c), highlighting human-specific upregulation despite the broader conserved primate expression program. In the mouse developing brain^57^, orthologous genes showed predominant expression in cortical neurons (Supplementary Fig. 10g). In contrast, genes upregulated in chimpanzee bRG did not display this neuronal-biased expression pattern (Supplementary Fig. 10h).

Together, these analyses indicate that human bRG-upregulated genes are embedded within conserved neuronal gene expression programs across species, but are more strongly or selectively activated in human bRG. This supports a model in which human bRG partially co-opt ancestral neuronal gene expression programs, potentially contributing to their enhanced morphological complexity.

Consistent with this, the 589 target genes of hbRG cCREs (Fig. 2e) also segregated into neuron-high and radial glia-high expression clusters (Supplementary Fig. 12a), with ‘neuron-high’ including genes such as *STMN2* (Supplementary Fig. 12b), a microtubule-binding protein regulating neuronal neurite outgrowths^58^, and *AUTS2* (Supplementary Fig. 12c), an autism risk gene previously shown to regulate actin dynamics and neurite formation in neurons^59^. Interestingly, ‘radial glia-high’ revealed a number of genes more highly expressed in chimpanzee neurons than neural progenitor cells (Supplementary Fig. 12d, chimpanzee cluster 1). Example genes include *PTPRM* (Supplementary Fig. 12e), a protein tyrosine phosphatase receptor shown to be critical for synapse formation^60^, and *NRG1* (Supplementary Fig. 12f), a stimulator of neurite outgrowth in various neuron types^61^. These species-specific expression patterns were broadly conserved in gorilla CeO, in human CO from two additional cell lines, and published chimpanzee and human organoids^29^ (Supplementary Fig. 12g‒i).

Taken together, these findings extend the concept of co-option to both gene expression and regulatory elements, suggesting that human bRG may have repurposed ancestral neuronal programs at multiple regulatory levels to modify, and potentially increase, their morphological complexity.

### Increased activity of human bRG morphoregulatory enhancers

**Fig. 4:**
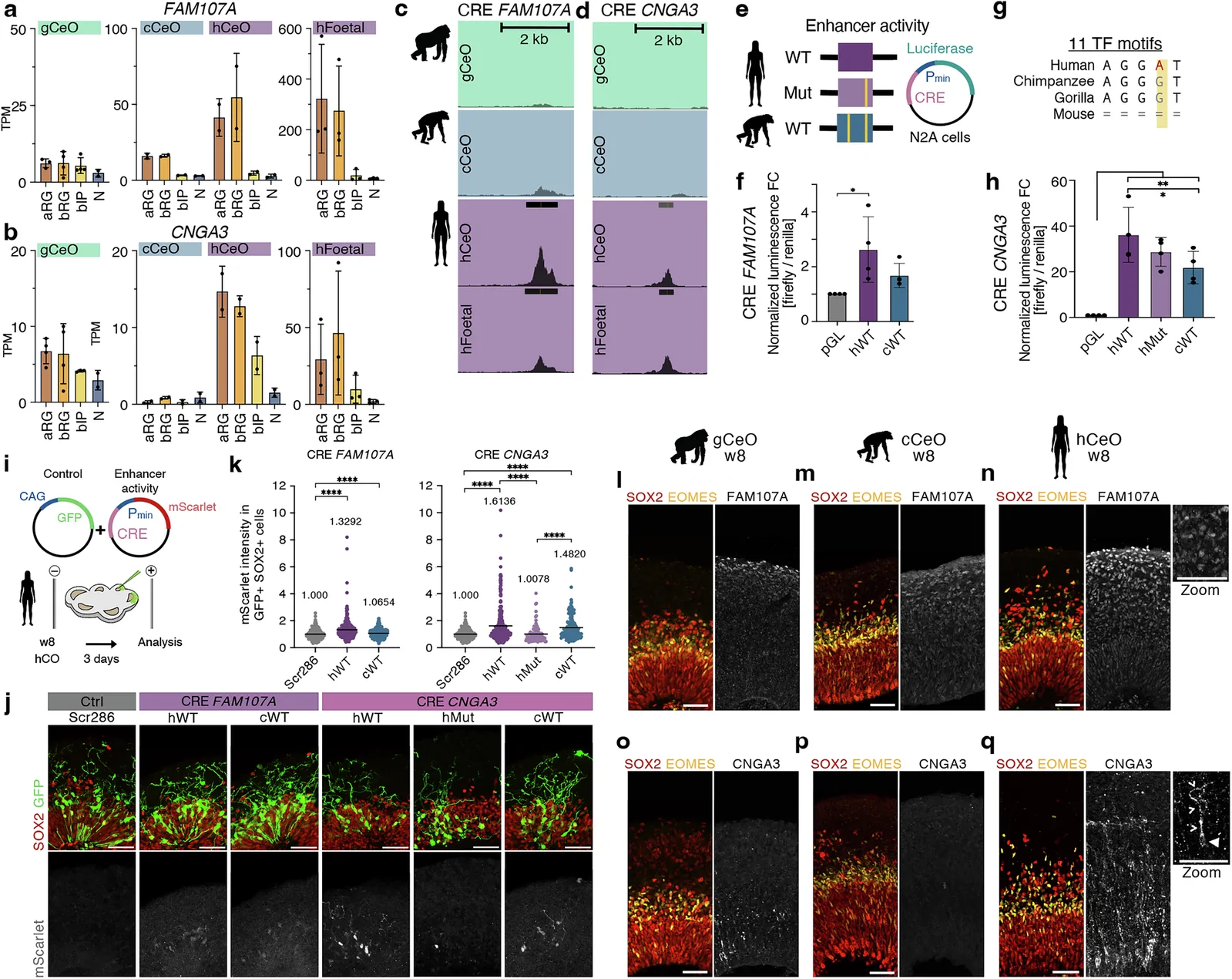
Inter-species differences in *FAM107A* and *CNGA3* gene regulatory networks and expression. Gene expression of **a** *FAM107A* and **b** *CNGA3* determined by RNA-seq (see Supplementary Data 2 and 3). Mean ± SD in transcripts per million (TPM). ATAC-seq for **c** *FAM107A*-linked hbRG cCRE (chr3: 59,505,735–59,506,833) and **d** *CNGA3*-linked hbRG cCRE (chr2: 98,149,173–98,149,634). Merged bigWig tracks of at least two replicates, with standardised track height 0–3. Black bars represent called peaks. **e** Luciferase reporter gene assays in mouse N2A cells for the 230 bp-centre hbRG cCRE human wild-type sequence, a mutated human sequence with a reverted chimpanzee nucleotide in the transcription factor binding site, and the chimpanzee sequence. **f** Normalised luminescence activity for the empty reporter (pGL) and human and chimpanzee wild-type *FAM107A*-linked hbRG cCRE sequences. Mean ± SD from four independent experiments; *p < 0.05, **p < 0.01; ANOVA with Tukey’s multiple comparison post hoc test. **g** The *CNGA3*-linked hbRG cCRE peak centre contains a human-specific nucleotide change, creating binding sites for eleven transcription factors (TF). **h** Normalised luminescence activity for the empty backbone, human and chimpanzee wild-type *CNGA3*-linked hbRG cCRE sequences, and human mutated sequence. Mean ± SD from four independent experiments; *p < 0.05, **p < 0.01; ANOVA with Tukey’s multiple comparison post hoc test. **i** Scheme of hCO electroporation. **j** Immunoh**i**stochemistry for SOX2 and GFP of an electroporated hCO. **k** Quantification of mScarlet intensity in SOX2 + GFP+ radial glia in hCO, relative to Scr control. Scale bar, 50 µm. ****p < 0.0001; one-way ANOVA with Dunnett post hoc test. 4–7 ventricles from 2–5 organoids per construct. Immunohistochemistry for SOX2, EOMES, and FAM107A in gorilla (**l**), chimpanzee (**m**) and human (**n**) CeOs shows protein expression in all cells, including bRG in the SVZ (zoom). Scale bars, 50 μm. 2–5 organoids. Immunohistochemistry for SOX2, EOMES, and CNGA3 in gorilla (**o**), chimpanzee (**p**) and human (**q**) CeOs shows protein expression in human radial glia, including bRG (zoom) and in gorilla radial glia but not in chimpanzee. Scale bars, 50 μm. 2–5 organoids. Source data are provided as a Source Data file.

As increased expression of morphoregulatory genes in basal progenitors was shown to be highly relevant for neocortex size evolution^13^, we aimed to functionally investigate CRE-gene pairs falling into this category. To select candidates, we screened our hbRG cCRE for regions linked to genes significantly upregulated in human bRG (log2FC > 1.5) that had a previously described morphoregulatory function and were also robustly expressed in human foetal bRG (TPM > 10). For functional analysis, we chose *FAM107A* for its previous association with human bRG biology^43^, and *CNGA3* for its association with both neurite outgrowth and synaptic plasticity in functional characterisation studies in the mouse retina^62^ and hippocampus^63^. *FAM107A*, encoding an actin-binding protein suggested as a key component of the human bRG molecular signature^43^, showed significantly higher gene expression in humans compared to gorilla and chimpanzee bRG (Figs. 3c, 4a and Supplementary Fig. 13a). CNGA3 is a cGMP-activated depolarising ion channel described to be expressed in cone photoreceptors, whose dysregulation leads to abnormal neurite outgrowth in the retina^62^. A recent study describes expression in non-cortical brain regions^64^. In CeOs, *CNGA3* expression was low in gorilla, largely absent in chimpanzees, but robustly expressed in human neural progenitor cells (Figs. 3c, 4b and Supplementary Fig. 13b). The cCREs targeting *FAM107A* (Fig. 4c) and *CNGA3* (Fig. 4d) showed higher accessibility in human compared to gorilla and chimpanzee bRG.

Luciferase reporter assays in N2A cells (Fig. 4e) revealed that only the human sequence of the *FAM107A* cCRE, and not the orthologous chimpanzee sequence, significantly drove reporter gene expression (Fig. 4f). This indicates that the human region has a higher gene regulatory potential than its chimpanzee ortholog. The *CNGA3*-linked hbRG cCRE has no ortholog in mice and contains within its central region a human-specific G-to-A conversion that creates putative binding sites for eleven transcription factors (Fig. 4g), including ELF1, previously shown to drive glioma proliferation^65^, and ELK1, essential for neuronal function and cytoskeletal dynamics^66^. Luciferase reporter assays showed substantial gene regulatory potential for both the human and chimpanzee *CNGA3* cCRE sequences, with the human sequence showing significantly higher activity (Fig. 4h). The hbRG cCRE targeting *CNGA3* could thus be shown to be a bona fide enhancer with higher activity potential for the human compared to the chimpanzee sequence.

To test whether these cCREs display enhancer activity also in human radial glia, we cloned the candidate regions into a plasmid containing a minimal promoter upstream of a fluorescent reporter. We electroporated the constructs into human COs^23^, along with a control plasmid expressing GFP, and assessed enhancer activity 3 days post-electroporation (Fig. 4i). The human sequences of the *FAM107A*- and *CNGA3*-linked CREs drove significant reporter gene expression in SOX2+ radial glia in human COs (Fig. 4j, k). The corresponding chimpanzee sequences showed a small, statistically insignificant reduction in reporter gene expression compared to the human sequence, whereas the mutation of the central human-specific transcription factor binding site in the *CNGA3* CRE led to a significant reduction in reporter gene expression. The enhancer activity assay, therefore, confirms that the two human bRG-linked CREs have the potential to enhance gene expression from a reporter plasmid in human COs.

To address whether the *FAM107A* CRE contributes to *FAM107A* expression in human cortical development, we targeted the genomic locus for silencing using a dCas9-KRAB-MeCP2 CRISPRi approach^67^ by electroporating human COs^68^. This led to a small, albeit statistically insignificant, reduction in *FAM107A* expression in PAX6+ radial glia (Supplementary Fig. 13c‒f), suggesting that the cCRE may have a small contribution to *FAM107A* expression in human cortical development, but that additional active enhancers likely compensate^69^ and maintain *FAM107A* expression. Likewise, dCas9-KRAB-MeCP2 CRISPRi targeting of the *CNGA3* CRE led to a small, statistically insignificant, reduction in gene expression (Supplementary Fig. 13f).

In line with RNA expression levels, FAM107A protein was expressed at higher levels in human compared to gorilla and chimpanzee CeOs (Fig. 4l‒n and Supplementary Fig. 13g). Of note, gorilla CeOs showed sparse CNGA3 protein expression in radial glia, while chimpanzee CeOs did not show protein expression of CNGA3 (Fig. 4o, p). As gorillas have both a larger brain and higher cortical neuron numbers compared to chimpanzees^5,70^, human-gorilla shared features are an interesting perspective. CNGA3 was clearly detectable and abundant in human CeOs and foetal tissue, including in cells with typical bRG morphology (Fig. 4q and Supplementary Fig. 13h, i). Interestingly, CNGA3 is expressed in some, but not all, radial glia; however, we could not observe a direct correlation between CNGA3 expression and the morphology of individual cells (Supplementary Fig. 13i).

Together, these findings demonstrate that example human bRG-associated CREs exhibit increased regulatory activity compared to their non-human primate orthologues and likely modulate the expression of genes implicated in morphoregulatory processes. This supports a model in which human-specific differences in bRG behaviour arise, at least in part, through quantitative changes in cis-regulatory activity within conserved gene regulatory networks.

### *FAM107A* and *CNGA3* regulate the morphological complexity of bRG

**Fig. 5:**
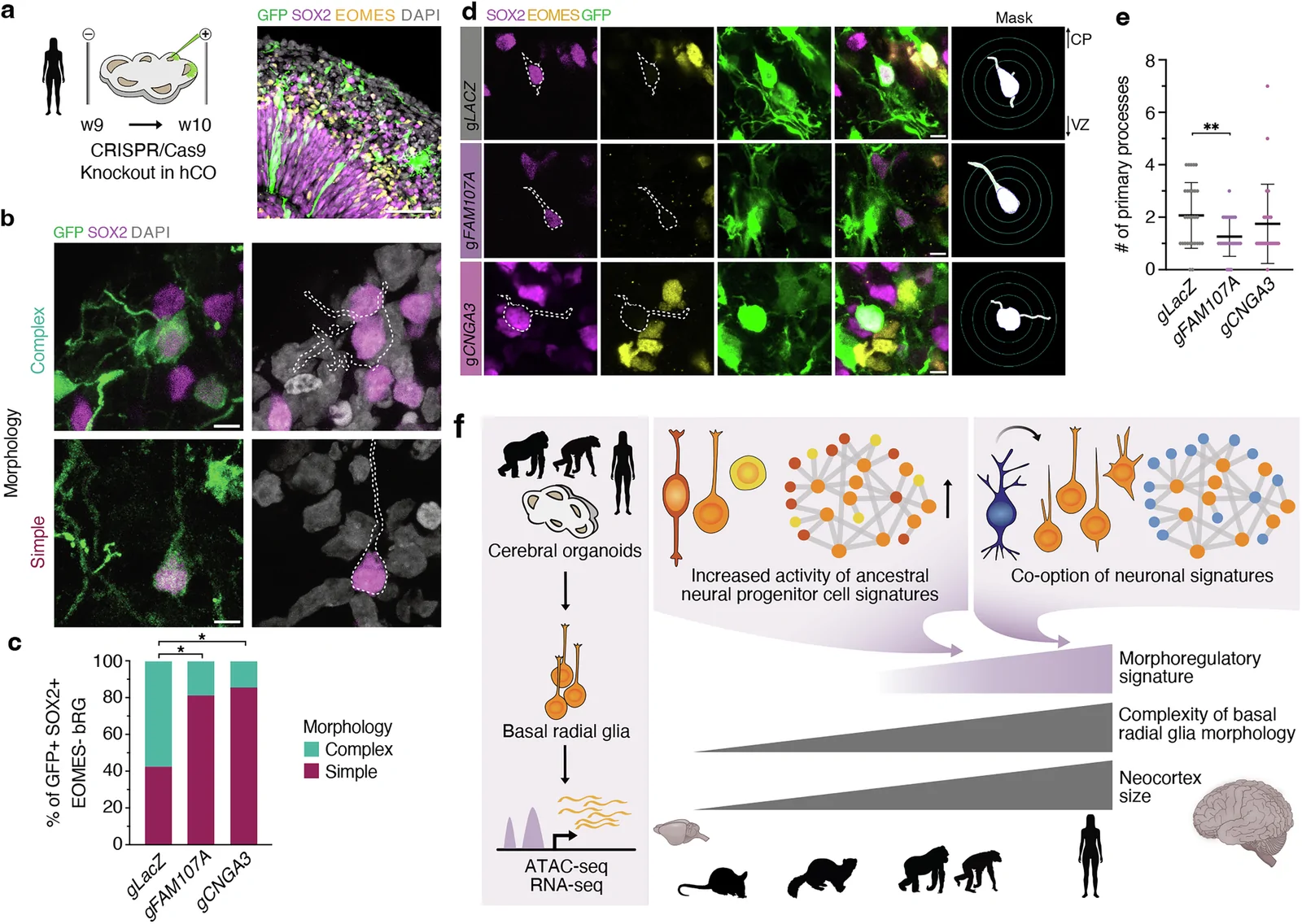
*FAM107A* and *CNGA3* are required for the morphological complexity of human bRG. **a** Scheme of the CRISPR/Cas9-mediated knockout in hCOs and electroporated hCO ventricle analysed by immunofluorescence for GFP, SOX2 and EOMES, and staining for DAPI. Scale bar, 50 μm. **b** Examples of electroporated GFP + SOX2+ bRG in the subventricular zone of electroporated hCOs with simple and complex morphologies in hCOs revealed by immunofluorescence for GFP and SOX2, and staining for DAPI. Scale bars, 5 μm. **c** Quantification of the relative distribution of bRG according to their morphological complexity^13,72^. Mean of 3 independent hCO differentiations and KO experiments. *p < 0.05; 2-way ANOVA with Bonferroni post hoc test. **d** Representative images of analysed SOX2+/EOMES– bRG in the subventricular zone of electroporated hCOs by immunofluorescence for GFP, SOX2 and EOMES, along with the relative cell mask. **e** Quantifications of the number of primary processes of bRG after *FAM107A* and *CNGA3* knockout. Mean ± SD of 3 independent hCO differentiations and KO experiments. **p < 0.01; two-sided Welch’s t-test. **f** Summary of findings. Inter-species comparison of bRG isolated from CeOs suggests that human-specific morphoregulatory signatures characterise neocortex evolution. Source data are provided as a Source Data file.

To directly assess whether *FAM107A* and *CNGA3* influence human bRG morphology, we performed CRISPR/Cas9-mediated knockout by electroporating human COs with ribonucleoprotein complexes^23,68^ (Fig. 5a). We confirmed efficient targeting in vitro and in human iPSCs, and reduced FAM107A and CNGA3 protein levels in organoids (Supplementary Fig. 14). We then analysed the morphology of SOX2 + EOMES– bRG in the subventricular zone of human COs (Fig. 5b), which contained different bRG morphotypes^71^, including monopolar, bipolar, multipolar, and nonpolar cells, as well as cells with bifurcated processes (Fig. 5b and Supplementary Fig. 15a). Morphological analysis revealed that the proportion of bRG with complex morphologies was reduced upon knockout of both *FAM107A* and *CNGA3* compared to control (Fig. 5c and Supplementary Fig. 15b), supporting a role for these genes in regulating human bRG morphoregulatory programs. While both genes are also expressed in aRG, their knockouts did not affect aRG canonical morphology (Supplementary Fig. 15c, d), indicating bRG-specific effects.

We next segmented individual SOX2 + EOMES– bRG, generated single cell masks and quantified a series of morphological parameters, as previously established in human COs^72^. Knockout of *FAM107A* significantly reduced both the number of all processes and primary processes that grow directly from the cell body, without affecting process length, branching, or arborisation, while *CNGA3* knockout did not significantly alter any of the measured morphological parameters (Fig. 5d, e; Supplementary Fig. 15e‒i).

The morphological complexity of bRG increases from rodents to carnivores to primates, and is linked to their proliferative capacity^73^. Namely, the most complex bRG morphotype in the macaque foetal cortex provides the largest number of neuronal progeny^12^. In line with this, increasing process growth in mouse basal progenitors leads to increased neuronal production^13,72^. Similarly, knockout of *FAM107A* in human CO led to a significant decrease in the number of HuCD+ KI67– neurons in the subventricular zone/cortical plate of human CO (Supplementary Fig. 15j, k), suggesting that FAM107A contributes to the morphological complexity of human bRG and its developmental consequences.

Increasing FAM107A levels in chimpanzee CeO by overexpression of human *FAM107A* from a CAG promoter produced a similar phenotype as the knockout in human (Supplementary Fig. 16). This suggests that *FAM107A* expression levels are tightly regulated by gene regulatory networks in the human developing neocortex to achieve appropriate expression levels. This is in agreement with previous work on morphoregulatory proteins, which showed that both too low and too high expression levels affected cell morphology in the same direction^74^.

Overall, our findings suggest that human bRG gene regulatory networks enhance the activity of morphoregulatory pathways, including *FAM107A* and *CNGA3*, likely contributing to the elaboration of bRG morphology in human cortical development and evolution (Fig. 5f).

## Discussion

In this study, we used a comprehensive genomics approach to identify human-specific gene-regulatory patterns of bRG that may underlie their unique biology. In light of the inaccessibility of foetal tissue from non-human great ape species, we generated three-dimensional great ape cerebral organoid models, from which we isolated bRG based on a combinational code of marker protein expression and cell biological features. This combined approach enabled an unbiased characterisation of human-specific bRG signatures, which led to the discovery of a consistent pattern of human bRG gene regulatory regions, their target genes, and human-upregulated genes involved in the regulation of neuronal cell morphology, in particular cell projections.

Neurons are characterised by great morphological complexity that underlies their specific connectivity patterns^75^. Our data show strongly increased accessibility in ancestral neural progenitor CREs as well as a subset of human bRG CREs that exhibit concomitant and ancestral activity in neurons, reflected in the expression patterns of their target genes. This dual expression pattern is conserved across species and developmental stages, indicating that these genes are embedded within ancestral neuronal programs rather than representing human-specific innovations. This suggests that human bRG gained activity in neural progenitor cell-active morphoregulatory CREs and genes, and may have repurposed neuronal gene regulatory networks to regulate and likely complexify their morphology. Notably, some of these patterns extend to human aRG, which share many molecular features with bRG. Different mechanisms contribute to the regulation of neuronal morphogenesis and neurite growth, including actin dynamics and neuronal activity^76^. In line with this, we show that the genes *FAM107A* and *CNGA3*, encoding an actin-binding protein^43^ and a cGMP-activated depolarising ion channel^62^, respectively, are functionally required for the morphological complexity of human bRG. Additionally, we identified several other genes linked to the regulation of cell growth and cell projections, which may act in concert with *FAM107A* and *CNGA3* to shape bRG morphology, stemness, proliferation, and neuronal output.

Increased morphological complexity and resulting increased proliferative potential of bRG have been shown to correlate with neocortex size^13^, likely mediated by the increased exposure to pro-proliferative signals from the extracellular environment^73^. The basal process of bRG receives signals from projection neurons, interneurons, blood vessels, the basal lamina and the extracellular matrix. A more complex bRG morphology was proposed to increase the surface area and to enable reaching new territories, providing access to signals from various sources^73^. Our data suggest that this pattern extends to a human-specific context and that morphoregulatory signatures distinguish human bRG from other great apes. We propose that the co-option of morphoregulatory and neuronal signatures is a key evolutionary mechanism contributing to these changes.

Given the inaccessibility of non-human great ape foetal tissue, cerebral organoids provide a powerful platform to study gene regulatory programs important for great ape neocortex development and cortical evolution. These organoid models have been shown to parallel important aspects of neural development, particularly during the first trimester, including cellular morphology and tissue architecture as well as overall gene expression patterns^34,51,77–79^. Additionally, the overall epigenomic landscape is conserved in neural organoids, including chromatin accessibility and active and repressive histone modifications^80–83^. As with any in vitro system, cerebral organoids harbour several limitations. Particularly relevant to the study of cortical evolution is the limited expansion and formation of the outer subventricular zone that happens during the second trimester of human foetal brain development. Further improvements of organoid media, such as the addition of Leukaemia Inhibitory Factor (LIF)^84,85^, may increase bRG proliferation and promote the generation of a structured outer subventricular zone. Moreover, potential inter-species differences in developmental timing may influence the manifestation of species-specific phenotypes, even though key developmental milestones during mid-neurogenesis are largely conserved across great apes^3,35^, and mid-neurogenic organoid development appears highly aligned between gorilla, chimpanzee and human^29,37,38^.

Taken together, we employed a unique approach to provide unbiased insights into human-specific molecular programs of bRG, a neural progenitor cell type that plays a central role in increased neuronal output and neocortex expansion during evolution^3,4,17^. Many adaptive sequence changes in noncoding regions are likely to have small effect sizes, making them hard to detect experimentally^86,87^. A specific trait may involve a small number of genomic changes with large effects, or many genomic changes with small effects^87^. Previous work has identified a limited number of human-specific genes with large effects that regulate bRG proliferation and neocortex size^3,4,17^. Yet, a large part of human-specific biology has been proposed to originate from the differential expression of shared genes^18–20^, many of which are expected to have smaller effects on neocortex evolution^86,87^. One example of a factor differentially expressed between mouse and primate species is the growth factor EPIREGULIN, which we previously found to regulate human basal progenitor cell proliferation^23^. Moreover, the human accelerated region HARE5 was reported to regulate the transcription of the FZD8 receptor and to modify radial glia cell behaviour^22^. Along the same lines, HAR1984 was recently shown to contribute to human-specific chromatin architecture and neurogenesis^24^. Here, we extend this concept and identify several hundred genomic regions with inter-species differences in CRE activity in great apes, influencing gene expression programs linked to cell morphology. Our study broadens our understanding of the biological processes that distinguish human basal radial glia from other great ape species and identifies human-specific morphoregulatory signatures as a characteristic of neocortex evolution.

## Methods

### Human foetal brain tissue

Human foetal brain tissue was obtained from two sources. Firstly, the Department of Gynaecology and Obstetrics, University Clinic Carl Gustav Carus of the Technische Universität Dresden, following elective pregnancy termination and informed written maternal consent and with approval of the local University Hospital Ethical Review Committee (IRB00001473; IORG0001076; ethical approval number EK 355092018). Second, human foetal material was provided by the Joint MRC/Wellcome Trust (grant# MR/X008304/1 and 226202/Z/22/Z), Human Developmental Biology Resource (http://hdbr.org). Foetal age was determined as gestation week (GW) 13 or 14 through ultrasound measurements of crown-rump length and other established developmental stage criteria. Due to the protection of data privacy, the sex of the human foetuses supplying the tissue cannot be disclosed; however, sex is unlikely to impact this study’s outcomes significantly. Tissue dissection was performed in Tyrode’s solution (TUD) or L15 media (HDBR), and the tissue was used for experimental purposes within 1–6 hours following the procedure. Human foetal brain tissue is protected and is not available for sharing due to data privacy laws and ethical permissions.

### Human and primate induced pluripotent stem cell lines

All iPSC lines were grown under standard conditions (5% CO_2_, 37 °C) on Matrigel-coated plates. The human iPSC lines WTC11 (UCSFi001-A; Gladstone Institutes, distributed by Coriell Institutes)^88^, HPSI0114i-kolf_2-C1 (Wellcome Trust Sanger Institute, HipSci) and CRTDi004-A^89^, and the chimpanzee iPSC line SandraA^77^ were cultured in mTeSR1, whereas StemFlex medium was used for the gorilla iPSC line GC1^90^. The lines WTC11, HPSI0114i-kolf_2-C1 and GC1 were passaged using ReLeSR. For CRTDi004-A and SandraA, TrypLE Express enzyme and Y-27632 Rho kinase inhibitor were used. All experiments involving human iPSCs were performed in accordance with the ethical standards of the institutional and/or national research committee, as well as with the 1964 Helsinki Declaration, and approved by the University Hospital Ethical Review Committee (IRB00001473; IORG0001076; ethical approval number SR-EK- 456092021).

### Generation of cerebral and cortical organoids

Cerebral organoids were generated as whole spheres using the STEMdiff Cerebral Organoid Kit or equivalent self-made medium based on previous publications with small modifications as described in detail^32,91^. Briefly, 9000 cells were seeded into 96-well Ultra-Low Attachment plates and cultured in mTeSR1 containing 10 µM Y-27632 Rho kinase inhibitor. Plates were spun for 3 min at 300 x *g* to settle cells. After 48 hours, the medium was changed to mTeSR1 without Y-27632 Rho kinase inhibitor (organoid formation medium). On day 4, the now-formed embryoid bodies (EBs) were changed to neural induction medium. EBs were embedded in Matrigel on day 8, transferred to differentiation medium containing B27 supplement without vitamin A (expansion medium), and henceforth cultured on an orbital shaker. Starting on day 14, young organoids were changed to, and then maintained in, differentiation medium containing B27 supplement with vitamin A (maturation medium), changed every 2–3 days until collection for experiments. All cerebral organoids were collected for experiments on w8 ± 3 days.

CRISPRi, the enhancer activity assay, and knockout experiments were carried out in sliced cortical organoids^31^ generated using the CRTDi004-A iPSC line, as described before^23,68^. In short, iPSC colonies were grown to a diameter of 1.5 mm and detached using collagenase for 1 hour at 37 °C to initiate EB formation in forebrain medium 1 in 6-well ultra-low attachment plates. Medium was changed on days 3 and 4 of organoid culture. Subsequently, half of the medium was replaced with forebrain medium 2 on days 5 and 6, before EBs were embedded in Matrigel cookies on day 7 in forebrain medium 2. After one week, cortical organoids were mechanically released from Matrigel and cultured in forebrain medium 3 on an orbital shaker at 120 rpm from then on. On day 35, forebrain medium 3 was supplemented with Matrigel for the remaining culture. Cortical organoids were sliced into 500 µm-thick slices using a vibratome at 45 days of culture. Medium was changed to forebrain medium 4 on day 63. Reagents are listed in Supplementary Information Table 2.

### Mouse Neuro-2A (N2A) cell line

The mouse neuroblastoma cell line was grown in standard conditions (5% CO_2_, 37 °C) on 6-well Nunclon Delta Surface plates. Cells were cultured in DMEM/F12 supplemented with 10% Foetal Bovine Serum and 1% Penicillin/Streptomycin. Cells were passaged using Trypsin-EDTA.

### Tissue labelling with the lipophilic dye DiI

The protocol for DiI-labelling was adapted from a previously published study^10^. For each experiment, about two-thirds of the experimental tissue was labelled in pre-warmed (37 °C) Tyrode’s solution with CellTracker CM-DiI Dye (1 mM in DMSO), the fixable variant of the previously published DiI for cell sorting^10^. More specifically, human foetal cortical tissue, roughly amounting to 30–50 million live cells, was labelled by lightly brushing a shortened microloader pipette tip over the entire basal surface of the cerebral hemisphere. After one passage, the hemisphere was transferred to a fresh TS-containing Petri dish to wash away excess dye. The labelling and washing process was repeated up to 5 times until the entire basal surface was uniformly coated in CM-DiI, as established by inspection under a fluorescent microscope. After labelling, the primary tissue was transferred to 20 mL glass flasks containing DMEM/F12 supplemented with 1x N2 and 1x B27, with 1–3 cortical fragments per flask. Tissue was incubated under rotation (FFTC)^92^ with 40% O_2_, 5% CO_2_ and 55% N_2_ at 37 °C in a whole-embryo culture incubator for 4 hours up to overnight.

For cerebral organoids, the entire surface of 5–10 organoids was labelled, and organoids were subsequently returned to culture in their respective medium for 3 hours. For all experiments, labelled and unlabelled tissue originated from the same organoid batch or from the same foetal sample, respectively. Unlabelled tissue was cultured separately but under the same conditions as labelled tissue. Post-incubation, organoids were further dissected in pre-warmed (37 °C) Tyrode’s solution to enrich for visible ventricle-like regions. Thus, for each experiment, a single piece of tissue or batch of organoids was split into two parallel pools (DiI-labelled and unlabelled), which were processed simultaneously under identical conditions.

### Immunocytochemistry

Experimental tissue was dissociated using the MACS Neural Tissue Dissociation Kit P with minor modifications for each tissue. For organoids, 3–6 organoids, depending on size, were combined in 1 mL enzyme mix 1 (50 µL enzyme P + 950 µL buffer X), incubated for 6 min at 300 rpm and at 37 °C in a thermoshaker, quenched with 30 µL enzyme mix 2 (10 µL enzyme A + 20 µL buffer Y), and conditions were recombined after trituration to single cells. For foetal tissue, up to 400 mg of tissue was combined in 1950 µL of enzyme mix 1 (50 µL enzyme P + 1900 µL buffer X), incubated for 15 min, quenched with 30 µL enzyme mix 2 (10 µL enzyme A + 20 µL buffer Y) and, if applicable, conditions recombined after trituration to single cells. After trituration to single cells, all tissues were spun for 5 min at 300 x *g* and 4 °C to pellet cells and resuspended in 1 mL cold TS. Cells were counted, and viability was assessed using 10 µL of the cell suspension by Trypan blue staining.

The protocol for immunocytochemistry, subsequent cell sorting and processing was adapted from previously established approaches^39,93^. All centrifugation steps were carried out at 4 °C unless otherwise noted. For fixation, cells were resuspended to no more than 20 million cells/mL and incubated at 1% v/v formaldehyde in TS for exactly 10 min at 10 rpm rotation at room temperature (RT). Fixation was quenched with 0.2 M glycine for 5 min at 10 rpm rotation. Cells were pelleted for 5 min at 500 x *g*, washed once in 1% BSA in TS and re-pelleted the same way. Cells were permeabilised on a rocking platform for 10 min at 4 °C in buffer containing 0.1% saponin and 0.2% BSA in Tyrode’s solution and subsequently pelleted for 3 min at 2,500 x *g*. For antibody staining, 4 million cells each were aliquoted from the unlabelled tissue condition for single-stain controls as well as an unstained control. From the DiI-labelled tissue condition, 4 million cells were aliquoted for a DiI-only control. Non-control conditions were continued in the presence of 1:1000 RNAsin Plus. Antibody staining was performed in the dark for 1 hour on a rocking platform at 4 °C in buffer containing 0.1% saponin and 1% BSA in Tyrode’s solution as follows: Human and primate DiI-labelled condition: V450 Mouse anti-SOX2 (1:40) or Rabbit anti-PAX6 (1:350), FITC Rat anti-CTIP2/BCL11B (1:500) or Rat anti-CTIP2/BCL11B (1:500) and Alexa Fluor 647 Mouse anti-ZO-1 (1:750). Human and primate unlabelled condition: V450 Mouse anti-Sox2 (1:40) or Rabbit anti-Pax6 (1:350), FITC Rat anti-CTIP2/BCL11B (1:500) or Rat anti-CTIP2/BCL11B (1:500), Alexa Fluor 647 Mouse anti-ZO-1 (1:750) and PE Mouse anti-EOMES (1:33). Subsequently, cells were pelleted for 3 min at 2,500x g and washed twice in buffer containing 0.1% saponin and 0.2% BSA in Tyrode’s solution. Conditions stained with Rabbit anti-Pax6 or Rat anti-CTIP2/BCL11B were subsequently stained with Goat anti-Rabbit Alexa Fluor 405 or Donkey anti-Rat Alexa Fluor 488 in the dark for 1 hour on a rocking platform at 4 °C in buffer containing 0.1% saponin and 1% BSA in Tyrode’s solution and then washed twice in buffer containing 0.1% saponin and 0.2% BSA in TS. All cells were resuspended to 1–3 million cells/100 µL in 0.5–1 mL 0.5% BSA and 1:1,000 RNAsin Plus in TS, passed through the 35 μm filter of their respective test tube and kept on ice for flow cytometry analysis. Antibodies are listed in Supplementary Information Table 2.

### Fluorescence-activated cell sorting (FACS)

Cells were sorted with a 100 µm nozzle with a sample chamber and tube holder at 4 °C. For each experiment, the drop delay was determined, and fluorophore compensation was calculated based on single-stain and unstained conditions. The main cell populations were defined using side scatter (SSC) area and forward scatter (FSC) area. Cell debris and aggregates were excluded using FSC height against FSC-A and SSC width against SSC-H. Cells were sorted using a BD FACSAria II with BD FACSDiva Software (v8.0.2 or v9.0.1). Cell populations were collected in DNA LoBind tubes containing either ATAC-RSB^94^ for subsequent ATAC-seq, or a mix of 95 µL RNAse-free H_2_O, 95 µL 2x Digestion Buffer and 10 µL Proteinase K (Zymo QuickRNA FFPE MiniPrep) for subsequent RNA isolation, and kept on ice until further processing.

For each experiment, cells of each population were sorted into separate tubes from the same cell suspension and using identical gating strategies: 50,000 cells for ATAC-seq, 10,000 or 5,000 cells for RNA-seq, and 5,000 cells for analysis by RT-qPCR. Deep-layer neurons (DL-N) and basal intermediate progenitor cells (bIPs) were isolated from cell suspensions derived from the unlabelled organoid pool. DL-N were defined as SOX2–/BCL11B+/ZO1–/EOMES– and gated in this hierarchy. bIPs were defined as not restricted on BCL11B expression (BCL11B±) and gated for EOMES+/SOX2–/ZO1– in this hierarchy. Apical and basal radial glia were isolated from cell suspensions derived from the DiI-labelled organoid pool. aRG were defined as SOX2+/ZO-1+/DiI+, while bRG were defined as SOX2+/ZO-1–/DiI+ and gated in this hierarchy. BCL11B staining was used as an internal biological control, but not gated, as radial glia target cells were always BCL11B–. SOX2 and PAX6 antibodies were used interchangeably.

### ATAC-seq library preparation

ATAC-seq was performed as previously described with minor alterations to the protocol^94^. Briefly, sorted cells are pelleted at 500 x *g* and 4 °C for 5 min. All supernatant was aspirated; cells were re-suspended in 50 µL ice-cold lysis buffer by pipetting up-and-down three times and incubated on ice for 3 min. Lysis was washed out with 1 mL ice-cold wash buffer, and the tubes were inverted to mix. Cells were again pelleted at 500 x *g* and 4 °C for 10 min. Supernatant was fully aspirated and cells were resuspended in 50 µL transposition mix containing 100 nM transposase. Reactions were incubated at 37 °C and 1000 rpm in a thermoshaker for 2 hours and then quenched by the addition of 40 mM EDTA. Reactions were pelleted at 500 x *g* for 5 min, re-suspended in 200 µL reverse-crosslink solution and incubated O/N at 65 °C and 1000 rpm in a thermoshaker, as previously reported for ATAC-seq on fixed cells^95^. The next day, the original protocol^94^ was continued, and reactions were cleaned up using a Zymo DNA Clean and Concentrator-5 Kit, libraries were amplified with determination of amplification cycles determined by qPCR as described^94^, and unique adapter combinations (i5 and i7) were used; and final PCR reactions were cleaned up using a Zymo DNA Clean and Concentrator-5 Kit. Final libraries were size-selected using SPRIselect Beads at 0.5x and 1.3x ratios to retain fragments of 150–1,000 bp. Successful transposition was established by the presence of a characteristic periodic nucleosomal pattern on a Fragment Analyzer NGS-Kit 1-6000 bp (Agilent); libraries were subsequently quantified using a Qubit fluorometer (Invitrogen), and at least 50 million fragments were sequenced with 100 bp paired-end mode on an S4 v1.5 200 cycles flow cell on the NovaSeq 6000 machine (Illumina).

### RNA-seq library preparation

RNA extraction was performed as per the manufacturer’s instructions using the Zymo QuickRNA FFPE MiniPrep Kit, and columns were eluted with 10 µL DNase/RNase-free H_2_O and stored at −80 °C until library preparation. Transcriptome libraries were prepared using the SmartSeq 2 protocol, modified from^96^. Isolated total RNA from 10,000 or 5000 cells was denatured for 3 min at 72 °C in 4 μL hypotonic buffer (0.2% Triton X-100) in the presence of 2.4 mM dNTPs, 240 nM dT-primer and 4 U RNase Inhibitor (NEB). Reverse transcription was performed at 42 °C for 90 min after filling up to 10 µL with RT buffer mix for a final concentration of 1x Superscript II buffer, 1 M betaine, 5 mM DTT, 6 mM MgCl_2_, 1 µM TSO-primer, 9 U RNase inhibitor and 90 U Superscript II Reverse Transcriptase. The reverse transcriptase was inactivated at 70 °C for 15 min. For subsequent PCR amplification of the cDNA, the optimal PCR cycle number was determined with an aliquot of 1 μL unpurified cDNA in a 10 μL qPCR containing 1x KAPA HiFi HotStart Readymix, 1x SYBR Green I and 0.2 μM UP primer. The determined cycle number was then used to amplify the residual 9 μL cDNA using 1x KAPA HiFi HotStart ReadyMix and 250 nM UP primer under the following cycling conditions: initial denaturation at 98 °C for 3 min, 12–17 cycles [98 °C 20 sec, 67 °C 15 sec, 72 °C 6 min] and final elongation at 72 °C for 5 min. The amplified cDNA was purified using 0.6x Sera-Mag SpeedBeads and resuspended in a buffer consisting of 10 mM Tris, 20 mM EDTA, 18.5% (w/v) PEG 8000 and 2 M sodium chloride solution. cDNA quality and concentration were determined using the Fragment Analyzer NGS 1-6000 bp Kit.

For library preparation, 2 µL amplified cDNA was tagmented in 1x Tagmentation Buffer using 0.8 µL bead-linked transposome (Illumina) at 55 °C for 15 min in a total volume of 4 µL. The reaction was stopped by the addition of 1 µL 0.1% SDS (37 °C, 15 min), beads were bound to a magnet and the supernatant was removed. Beads were then resuspended in 14 µL indexing PCR Mix containing 1x KAPA Hifi HotStart Ready Mix and 700 nM unique dual indexing primers (i5 and i7), and subjected to a PCR (72 °C 3 min, 98 °C 30 sec, 12 cycles [98 °C 10 sec, 63 °C 20 sec, 72 °C 1 min], 72 °C 5 min). Libraries were purified with 0.9x volume Sera-Mag SpeedBeads, followed by a double size selection with 0.6x and 0.9x. Repurification with a final 0.9x bead purification was performed if needed. Sequencing was performed after quantification using the FragmentAnalyzer NGS 1-6000 bp Kit (Agilent) on a NovaSeq S4 v1.5 200 cycles flow cell (Illumina) to a sequencing depth of at least 50 million 100 bp paired-end reads per library.

### Gene expression analysis by RT-qPCR

RNA extraction was performed as per the manufacturer’s instructions using the Zymo QuickRNA FFPE MiniPrep Kit. cDNA was synthesised using random hexamers and Superscript III Reverse Transcriptase. Final products were diluted by the addition of 180 µL H_2_O and stored at −20 °C until further analysis by RT-qPCR. qPCR was performed in technical triplicate using LightCycler 480 SYBR Green I Master on a LightCycler 480 (Roche). Data were normalised by *GAPDH* expression. Primers are listed in Supplementary Information Table 1.

### Cloning

The center 230 bp of candidate regions for *FAM107A* and *CNGA3* were synthesised for the human, human-mutated and chimpanzee sequences with restriction sites (IDT gBlocks Gene Fragments) for KpnI and BmtI. Fragments were inserted into the pGL4.23[luc2/minP] backbone (Promega) using NEB KpnI-HF and NEB BmtI-HF with CutSmart buffer according to the manufacturer’s instructions and confirmed by Sanger sequencing. Sequences are listed in Supplementary Information Table 1. For additional analysis in organoids, the same sequences were cloned by the company GenScript into the pMPRA1 backbone (Addgene #49349)^97^ that harbours a minimal promotor (minP) and the reporter gene mScarlet. For CRISPRi-mediated silencing, sgRNAs were cloned into a BFP-tagged pCRISPRia-v2 vector, a gift from Jonathan Weissman (Addgene #84832)^98^ as described before^99^. The correct sgRNA sequences were confirmed with Sanger sequencing (Eurofins Genomics).

### Luciferase assay for candidate CRE activity

N2A cells were seeded at a density of 10,000 cells/well in a white-walled, clear-bottom 96-well plate one day before lipofection. Lipofection was performed according to the manufacturer’s instructions using Lipofectamine 2000 DNA Transfection Reagent (Invitrogen) with 2.5 µL of Lipofectamine, 50 ng pRL-CMV (Promega) vector for normalisation and 450 ng of pGL4.23[luc2/minP]-construct vector.

Luciferase assays were performed as previously described^100^. Specifically, 24 hours after lipofection, the Dual-Luciferase Reporter Assay System from Promega was used according to the manufacturer’s instructions in concert with the EnVision Multimode plate reader by PerkinElmer to quantify CRE activity of candidate regions. All experiments were performed in triplicate wells/construct and 3 technical measurements/well were acquired for both Firefly (pGL4.23[luc2/minP]-construct vector) and Renilla (pRL-CMV). Raw Firefly measurements were normalised to Renilla activity for each well. The experiment was independently repeated four times.

The arithmetic means of Firefly/Renilla triplicate technical measurements in each well were calculated and subsequently, the values from all three wells/construct averaged for each experiment. Shapiro-Wilk and Levene’s test were used to assess normality and homoscedasticity, respectively, followed by one-way ANOVA and Tukey’s post-hoc. The cut-off for significance was set to *p* < 0.05.

### Enhancer activity assay in human cortical organoids

Organoids were co-electroporated at week 8 with the pMPRA1-CRE plasmids and pCAG-EGFP as described before^23,49^, fixed three days after transfection. The organoids were cryosectioned and immunohistochemistry was performed for the radial glia marker SOX2 and the reporter genes EGFP and mScarlet (see below). CRE activity was quantified as mScarlet intensity in GFP + SOX2+ cells relative to the scrambled control.

### ATAC-seq data analysis

ATAC-seq libraries were mapped and processed using the ENCODE pipeline^101^ with default settings. GorGor6, panTro6 and hg38 were used as the reference genomes for gorilla, chimpanzee and human, respectively. Putative orthologous regulatory regions were identified using the HALPER tool^40,102^ after generating reference-free Cactus multiple sequence alignments of the gorGor6, panTro6 and hg38 genomes^103^. The orthologous regions were merged to generate a common peak set (225,395 peaks) with the genomic coordinates of each corresponding species. Distal chromatin peaks refer to all peaks that do not overlap a transcription start site. DESeq2^104^ was used to calculate differentially accessible regions based on species (gorilla, chimpanzee and human) and cell type (aRG, bRG, bIP and N). Differentially accessible regions were intersected with genome coordinates of previously assembled candidate cis-regulatory elements^45^ and human-accelerated regions^47^. cCRE were linked to their target genes by generating all possible pairs with transcription start sites (TSS). Distal peaks were associated with genes located within the same topologically associating domain (TAD) that had the highest Hi-C score, provided the distance ranged between 5 kb and 2 Mb. These analyses were performed using previously generated published Hi-C data from organoid RGs and IPs^49^. GO term analysis^105,106^ on target genes was performed using the packages ‘enrichr’ (v3.2), ‘org.Hs.eg.db’ (v3.19.1) and ‘clusterProfiler’ (v4.12.6).

### Comparison of chromatin accessibility peaks with published organoid datasets

We compared our human and chimpanzee organoid ATAC-seq peak sets with published organoid ‘d60d120’ peaks from Kanton et al.^29^. Peak calls from aRG, bRG, bIP, and neuronal CeO populations were merged into species-specific non-redundant peak sets. Published human and chimpanzee peaks were lifted to hg38 and panTro6, respectively, and non-canonical contigs were removed. For direct comparison, all peaks were resised to 500 bp centred on the peak midpoint, and overlaps were identified using GenomicRanges::findOverlaps. Concordance was quantified as the fraction of peaks in each dataset overlapping the other dataset and by the Jaccard index. Statistical significance of overlap was assessed by Fisher’s exact tests using ENCODE cCREs as the background universe and by permutation testing (10,000 iterations) based on random sampling of cCRE intervals.

### Transcription factor motif analysis

The JASPAR2020 core vertebrate database was used for motif-based analysis. Transcription factor motif enrichment was calculated using the monaLisa package (v1.10.0)^107^. ‘Motifmatchr’ (v1.26.0) was then used to identify transcription factor motifs within genomic regions (p.cutoff = 0.0005) and center the region around them.

### RNA-seq data analysis

RNA-seq libraries were mapped and de-duplicated using STAR^108^ with default settings. Hg38 was used as the reference genome for human, chimpanzee and gorilla^109^. In line with human-origin tissue data protection, all genes annotated to the X and Y chromosomes based on Ensembl BioMart GRCh38.p14 annotations were excluded from analysis and are not listed in Supplementary Data 2. All downstream analysis was conducted in R v4.4.1. DESeq2^104^ was used to calculate FPKM and differentially expressed genes (adjusted *p* ≤ 0.05, log2FC = 1) based on species (chimpanzee and human) and cell type (aRG, bRG, bIP, and N). GO term analysis^105,106^ was performed using the packages ‘enrichr’ (v3.2), ‘org.Hs.eg.db’ (v3.19.1) and ‘clusterProfiler’ (v4.12.6). The morphoregulatory pathway activity score was adapted from a previous publication^53^ and retained all original code reported therein, merely exchanging the supplied GO-Term genes. In short, pathway activity scores were computed from RNA-seq expression data using gene sets obtained from MSigDB Gene Ontology biological process annotations related to cell morphogenesis using the GO terms ‘GOBP Cell morphogenesis.v2024.1’, ‘GOBP Cell projection morphogenesis.v2024.1’ and ‘GOBP Cell projection organization.v2024.1. Gene expression values (TPM) were averaged across biological replicates and mapped from Ensembl identifiers to HGNC gene symbols. Genes present in the selected pathways were extracted, and for each pathway, gene expression was averaged across samples of the same cell type and normalised by the mean expression of each gene across all cell types to obtain relative expression ratios. Pathway activity was then calculated as the weighted mean of these normalised gene expression values, where gene weights were defined as the inverse of the number of pathways in which each gene appeared, thereby reducing the influence of genes shared across multiple pathways. Statistical significance of pathway activity scores was assessed using permutation testing (1000 iterations) with randomised sample labels. For TPM values, RNA-seq libraries were aligned to GRCh38 using kallisto (v0.46.1)^110^ after quality control using FastQC (v0.11.6). TPM values were obtained in R using ‘Tximport’^111^ using the input ‘lengthScaledTPM’ together with the ‘EnsDb.Hsapiens.v86’ package.

### Analysis of published scRNA-Seq data

Pre-processed single-cell RNA-seq data, including alignment and annotation, were obtained in RDS format from Mendeley Data as provided by the authors of the original study^29^. From this dataset, we used human and chimpanzee brain organoid samples collected at 2, 3, and 4 months of age. Further processing was performed using Seurat v5.3.1^112,113^ and Seurat objects corresponding to each species were integrated prior to downstream analyses.

To assign cell identities using ArchMap^114^ with HNOCA^34^, the RDS objects were converted to H5AD format. After cell type annotation, the corresponding ArchMap object was downloaded and used for label transfer to the original datasets. Radial glial cells were further refined within clusters initially annotated as neural progenitor cells (NPCs). Composite marker scores were calculated using known radial glia markers (*SOX2*, *PAX6*, *VIM*, *FABP7*, *NES*, *HES1*, *HOPX*, *SLC1A3*, *GLI3*) and intermediate progenitor markers (*EOMES*, *ASCL1*, *SSTR2*, *NEUROG2*, *INSM1*, *PPP1R17*), with intermediate progenitor markers used as a control in the scoring procedure.

Following label transfer, datasets were filtered by developmental stage, retaining only cells from 2-month-old organoids. Standard quality control filtering was then applied, including removal of cells expressing fewer than 100 genes, genes detected in fewer than three cells, and predicted doublets. The resulting datasets were used for downstream analyses, including heatmap visualisation and violin plots.

### Gene expression analysis with the gEAR platform

We used the gEAR platform^115^ to explore gene expression patterns of the 465 hbRG DEGs across publicly available multi-omic datasets^55–57^. For UMAPs, Gene expression projection values were calculated using the platform’s projection tool (Principal Component Analysis, z-score-normalised gene expression) and downloaded at single-cell resolution to optimise visualisation. To improve interpretability, we applied a consistent processing strategy to the projection values. Specifically, negative coefficient weights, which are not biologically meaningful in this context, were set to zero. This step was performed uniformly across all datasets. For improved visualisation of gene expression enrichment across cell types, we implemented a grey-to-purple colour scale and assigned distinct colours to individual cell types. Additionally, cell representations were enlarged to improve readability. These adjustments were applied solely at the visualisation level and did not alter the underlying data or introduce analytical bias.

To examine the expression of candidate genes across defined cellular subpopulations, we used the NeMO Analytics platform (gEAR framework^115^). Publicly available scRNA-seq datasets^29,51,52,56^ were queried using the dataset curator tool to generate single-gene expression visualisations, with cells filtered based on annotated cell types and developmental stages.

### Electroporation of organoids

CRISPR/Cas9 gene ablation in human cortical organoids was carried out as described before^23,68^. Briefly, cortical organoids were sliced to 500 µm thickness two days before electroporation using a vibratome. On the day of electroporation (day 63 of hCO culture), ribonucleoproteins (RNP) were assembled freshly and separately per guide RNA (gRNA). Two gRNAs, each targeting either *FAM107A* or *CNGA3*, were used, as well as one gRNA targeting *LacZ* as a control (sequences are listed in Supplementary Information Table 1). Both RNP complexes targeting the same gene were mixed at an equimolar ratio, and 225 ng/µL pCAG-EGFP was added, as well as 0.1% FastGreen in H_2_O. Overexpression of candidate genes in 7-week-old chimpanzee cerebral organoids was carried out using a plasmid encoding the human gene *FAM107A* (450 ng/µL pCAG-*FAM107A*) mixed with 450 ng/µL pCAG-EGFP and 0.1% FastGreen in H_2_O. For both LOF and GOF experiments, organoids were collected in pre-warmed TS, and outwards-facing ventricle-like structures were injected with the prepared mixture, respectively. Injected organoids were transferred into an electroporation chamber in TS, and electroporation was carried out with five pulses applied at 38 V for 50 ms each at intervals of 1 s. Afterwards, electroporated organoids were transferred into their respective medium and cultured on an orbital shaker for 7 days before immunofluorescent analysis.

### CRISPR/Cas9-mediated gene knockout

Guide RNA design and validation for CRISPR/Cas9 gene knockout were performed as previously described^23,68^. First, each gRNA was tested separately in vitro using PCR products spanning the target region in the genes of interest. After digesting the PCR products with the RNP, the fragments were analysed by gel electrophoresis. Second, the two guide RNAs targeting the same gene were nucleofected together with a pCAG-EGFP plasmid into the same iPSC line that was also used for the generation of human cortical organoids, namely CRTDi004-A, with the P3 Primary Cell 4D Nucleofector X Kit S. After 3 days of culture, cells were sorted for GFP by FACS and genomic DNA was extracted. Targeted regions were amplified by PCR, purified after gel electrophoresis and analysed by Sanger sequencing.

### CRISPRi in human cortical organoids

To further investigate the CRE connection to the target genes, 6-week-old human cortical organoids were electroporated with a pCAG-dCas9-KRAB-MeCP2-mCherry plasmid and plasmids encoding BFP and guide RNAs targeting candidate CREs for *FAM107A* and *CNGA3*, respectively. As a control, the pCAG-dCas9 plasmid was co-electroporated with an empty pCAG-HA plasmid. After three days of incubation, organoids were processed for FACS as described above and 5000 PAX6+ mCherry+ BFP+ triple-positive cells were collected. RNA was extracted using the Zymo QuickRNA FFPE MiniPrep kit and gene expression analysis by RT-qPCR for *FAM107A* or *CNGA3* was performed as described above. Per candidate region or control, 6–10 pools consisting of 2 organoids each were processed individually. No statistics were performed due to the small sample size for the effect size.

### Immunohistochemistry and imaging

All tissue was fixed in 4% paraformaldehyde in 120 mM phosphate buffer, pH 7.4, at 4 °C for 30 min (organoids) or O/N (human primary tissue). Tissue was transferred to 15% sucrose for 24 hours (human primary tissue only), transferred to 30% sucrose for at least 24 hours, embedded in 50:50 O.C.T. compound: 30% sucrose in PBS and cut into 20 µm (organoid) or 12 µm (human primary) sections using a cryostat. For immunofluorescence, antigen retrieval was performed in 10 mM citrate buffer pH 6.0 for 1 hour at 70 °C and washed out three times with PBS. Sections were quenched in 0.1 M glycine in PBS at RT for 30 min and again washed in PBS. Sections were permeabilised for 30 min at RT in 0.1% Triton X-100 and 10% horse serum and subsequently incubated with primary antibodies in the same buffer O/N. After 3 washes with PBS, sections were incubated with secondary antibodies and DAPI (all 1:1,000) for 1 hour at RT in 0.1% Triton X-100 and 10% horse serum before washing out with PBS and mounting in Mowiol. Images were acquired with a Zeiss ApoTome2 fluorescence microscope using a 10x or 20x objective and 1.5 µm thick optical sections, a Zeiss LSM 780/FCS microscope using a 20x, 40x (water immersion) or 63 x (oil immersion) objective, or a Zeiss LSM 980/MP microscope using a 20x or 63 x (oil immersion) objective and 0.3 µm thick optical sections. When images were taken as tile scans, the stitching of tiles was performed using the ZEN software. Antibodies are listed in Supplementary Information Table 2.

### Image analysis and protein quantification

Quantification was performed using Fiji/ImageJ^116^, processed using Excel (Microsoft), and plotted using Prism (GraphPad Software). Image quantification was performed either manually or using the Fiji plugin StarDist^117^. Briefly, for zone-specific quantification of CNGA3 expression, images were analysed as maximum intensity projections and first cropped to separate the different cortical layers (VZ, SVZ/CP). Subsequently, all channels were split and CNGA3 intensity was quantified per zone. For quantification of nuclear signal intensity of FAM107A, KI67, HuCD and mScarlet, the zone and channel separation was followed by segmentation using the StarDist 2D plugin in the versatile (fluorescent nuclei) model in the DAPI channel. If necessary, segmentation was corrected manually. Segmentation is followed by signal intensity measurements of the markers of interest. For cortical organoids, only ventricles facing the outside of the organoid were analysed, as ventricles in the organoids’ centre might suffer from reduced oxygen and nutrient supply^31^. Shapiro-Wilk and Levene’s test were used to assess normality and homoscedasticity, respectively, followed by Mann-Whitney-U test or Student’s t-tests or one-way ANOVA with Dunnett post hoc test (MPRA). The cut-off for significance was set to *p* < 0.05.

### Image analysis and cell morphology

Morphological analyses of radial glia for both KO and over expression studies were performed as established earlier^72^. Briefly, bRG were identified as GFP + SOX2 + EOMES– cells in the electroporated subventricular zone. Single cells were isolated by cropping an area of 53.3 μm x 53.3 μm surrounding the cell. If the cell was protruding outside the established borders, a larger selection was made. Morphological analysis was conducted by manually segmenting the cell through a Z-stack using GFP signal as a reference to the cell shape. Morphological parameters were then quantified on the obtained cell mask using previously established pipelines^13^. Each analysed bRG was then assigned to a particular morphotype according to the number and the orientation of processes with respect to the cell body, defining monopolar, bipolar, bifurcated, multipolar or nonpolar cells, as previously established^72^. Morphotype assignment of SOX2 + GFP+ aRG morphotypes in the ventricular zone of electroporated organoids was carried out as previously described^72^. Shapiro-Wilk and Levene’s test were used to assess normality and homoscedasticity, respectively, followed by Mann-Whitney-U, Welch’s t-test, and 2-way ANOVA with Bonferroni post hoc test. The cut-off for significance was set to *p* < 0.05.

### Schematics

Schemes accompanying figures have been generated using Adobe Illustrator and Affinity Designer software.

### Reporting summary

Further information on research design is available in the Nature Portfolio Reporting Summary linked to this article.

## Supplementary information

Supplementary Information

Description of Additional Supplementary Files

Supplementary Data 1

Supplementary Data 2

Supplementary Data 3

Reporting Summary

Transparent Peer Review file

## Source data

Source Data

## Data Availability

Non-human great ape raw and processed RNA-seq and ATAC-seq data generated in this study have been deposited in the Gene Expression Omnibus (GEO) database under accession number GSE331438 (https://www.ncbi.nlm.nih.gov/geo/query/acc.cgi?acc=GSE331438). De-identified human and great ape ATAC-seq data have been deposited at Zenodo (https://doi.org/10.5281/zenodo.14745099). The raw human data are protected and are not available due to data privacy laws and ethical permissions. Processed ATAC-seq and RNA-seq data are available in Supplementary Data 1–3. Source data are provided with this paper.

## Code availability

This study has not generated new code. All code was previously published and is cited accordingly.
