# Supplementary material for: Human-specific morphoregulatory signatures in basal radial glia characterise neocortex evolution
Source: Nat Commun. 2026 Oct 9;17:10651. doi: 10.1038/s41467-026-78474-0 (PMC13652802; doi:10.1038/s41467-026-78474-0)
Supplement: Supplementary file 1 [file 41467_2026_78474_MOESM1_ESM.pdf]

Human-specific morphoregulatory signatures in basal radial glia characterise neocortex evolution

Theresa M. Schütze, Annika Kolodziejczyk, Nora Ditzer, Silvia Vangelisti, Emanuele Capra, Jeong-Eun Lee, Ilaria Chiaradia, Vida Kufrin, Seiya Yamada, Jula Peters, Maximilian Krause, Christina Eugster, Razvan P. Derihaci, Cahit Birdir, Takashi Namba, Alexander A. Wurm, Ulrich Martin, Pauline Wimberger, Katherine R. Long, Madeline Lancaster, Nereo Kalebic, Boyan Bonev and Mareike Albert

Supplementary information

Supplementary Figures 1–16

Supplementary Figure 1. Characterisation of cerebral organoids and foetal tissue.
Supplementary Figure 2. Fluorescence activated cell sorting and validation of cell populations.
Supplementary Figure 3. Characterisation of chromatin accessibility patterns across cell types and species.
Supplementary Figure 4. ATAC-seq of two independent iPSC-line-derived human cortical organoids recapitulate accessibility patterns.
Supplementary Figure 5. Analysis of published human and chimpanzee CeO scATAC-seq shows high concordance between datasets.
Supplementary Figure 6. RNA-seq reveals cell type- and species-specific expression patterns and confirms tissue identity.
Supplementary Figure 7. Expression of human bRG upregulated genes in human COs and gorilla CeOs corroborates human-upregulated expression. a
Supplementary Figure 8. Published scRNA-seq data reveals comparable expression patterns of human bRG upregulated genes across cell lines and organoid stages.
Supplementary Figure 9. Validation of morphoregulatory pathway activity score in the context of the developing cortex.
Supplementary Figure 10. Human bRG upregulated genes show a dual expression pattern in radial glia and neuronal populations across development and species.
Supplementary Figure 11. Morphoregulatory candidate gene expression in macaque foetal telencephalon corroborates human bRG upregulated expression.
Supplementary Figure 12. Clustering of hbRG cCRE target genes by gene expression.
Supplementary Figure 13. FAM107A and CNGA3 show human radial glia–enriched expression and are regulated by distal cis-regulatory elements in hCOs.
Supplementary Figure 14. CRISPR/Cas9-mediated knockout of FAM107A and CNGA3.
Supplementary Figure 15. FAM107A and CNGA3 alter bRG morphotype distribution, with selective effects of FAM107A on morphology and neurogenesis.
Supplementary Figure 16. Overexpression of FAM107A in chimpanzee CeO.

Supplementary Information Table 1. Oligonucleotides.
Supplementary Information Table 2. Reagents and resources.

Supplementary Figure 1

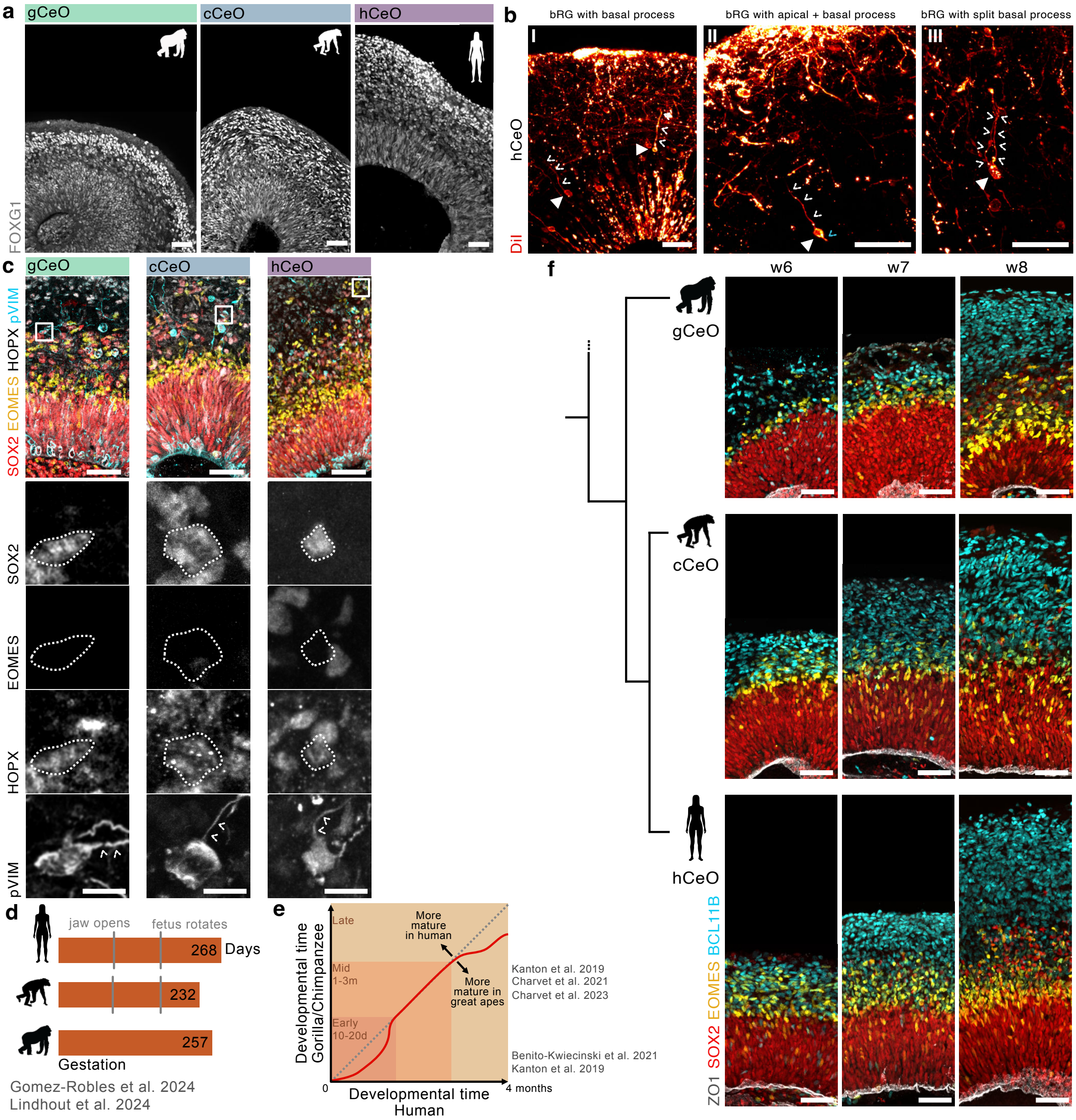

Supplementary Figure 1. Characterisation of cerebral organoids and foetal tissue. a, CeOs of gorilla, chimpanzee and human at w8 express the forebrain marker FOXG1, denoting cortical identity. Scale bars, 50 µm. **b**, Human CeOs at w8 labelled with the lipophilic dye DiI reveal cells with typical bRG morphology, including previously described morphotypes with (I) a single basal process, (II) an apical and basal process, and (III) a bifurcated basal process. Filled arrowheads denote bRG cell bodies, white open arrowheads bRG basal processes and blue open arrowheads bRG apical processes. Scale bars, 50 µm. **c**, Immunohistochemistry for SOX2, EOMES, HOPX and pVIM in gorilla, chimpanzee, and human CeOs demark bRG in the subventricular zone (inset). White open arrowheads denote bRG basal processes. Scale bars, 10 µm. **d**, Comparison of gestation length and timing of *in utero* developmental milestones between gorillas, chimpanzees, and humans indicates that early developmental events are highly correlated in timing[1,2]. **e**, Available data from cerebral organoid models indicate faster progression of the neuroepithelial-to-aRG transition (Early) and faster neuronal

maturation (Late) in chimpanzee and gorilla, whereas temporal dynamics in mid-neurogenic events – the subject of this study – are highly aligned[3-6]. **f**, Immunohistochemistry for ZO1, SOX2, EOMES and BCL11B in gorilla, chimpanzee, and human CeOs at week 6, 7 and 8 shows matched progression of germinal zone development and cell-type specification. Scale bars, 50 μm.

Supplementary Figure 2

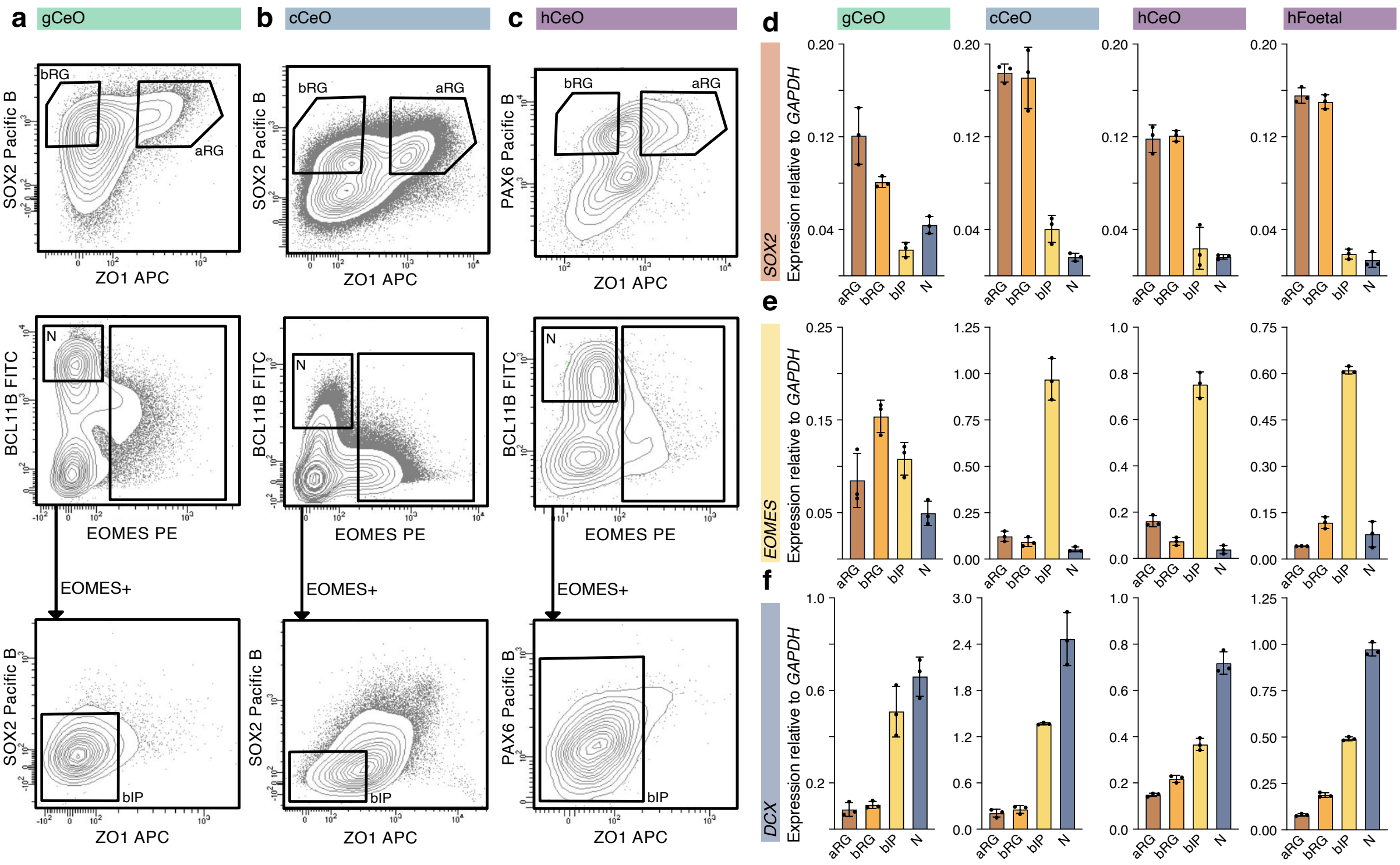

Supplementary Figure 2. Fluorescence activated cell sorting and validation of cell populations. a–c, FACS plots of gorilla, chimpanzee and human CeO. Top: Separation of aRG and bRG based on expression of ZO1. SOX2/PAX6 is expressed in both RG populations. Both cell populations are further sub-gated for DiI to select cells with basal processes. Middle: Gating for neurons was based on BCL11B+/EOMES–. Gating for bIPs was based on EOMES+ (middle) and SOX2–/ZO1– (bottom). **d–f**, RT-qPCR of the canonical radial glia marker gene *SOX2*, the bIP marker gene *EOMES*, and the neuronal gene *DCX*, normalised to the housekeeping gene *GAPDH*, in sorted cell populations of gCeO, cCeO, hCeO, and hFoetal confirms cell identity on RNA level but reveals slight differences relating to *EOMES* expression of gCeO progenitors. Error bars represent standard deviation. Source data are provided as a Source Data file.

Supplementary Figure 3

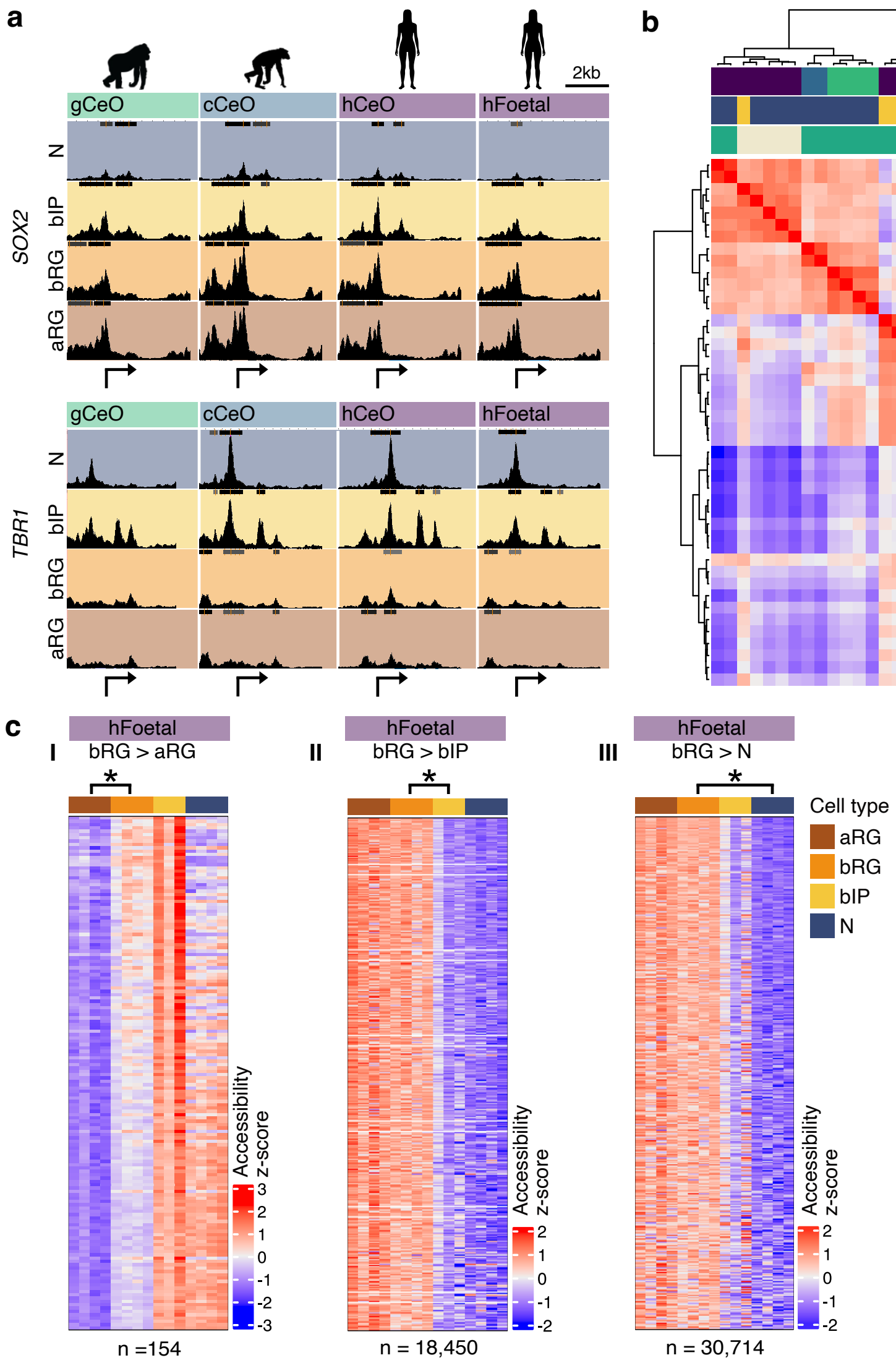

Supplementary Figure 3. Characterisation of chromatin accessibility patterns across cell types and species. a, Cell type-specific chromatin accessibility exemplified at the transcription start sites (arrows) of the radial glia gene *SOX2* (top) and early neuronal gene *TBR1* (bottom) for each species. Shown are merged bigWig tracks, with standardised track height 0–4.5. Called peaks are represented by black bars in tracks. **b**, Correlation heatmap of chromatin accessibility for all called peaks across all tissues and cell types, indicating patterns of cell type- and species-specific accessibility. **c**, Chromatin accessibility patterns across all cell types of human foetal cortex tissue of differentially accessible regions (log2FC $\geq$ 1; p $\leq$ 0.05), comparing (i) aRG vs. bRG, (ii) bIP vs. bRG, and (iii) N vs. bRG, underlining that bRG possess a unique molecular profile of sharing variable accessibility features with other cell types rather than being defined by 'bRG-specific' chromatin accessibility. Accessibility z-scores are shown for all samples.

Supplementary Figure 4

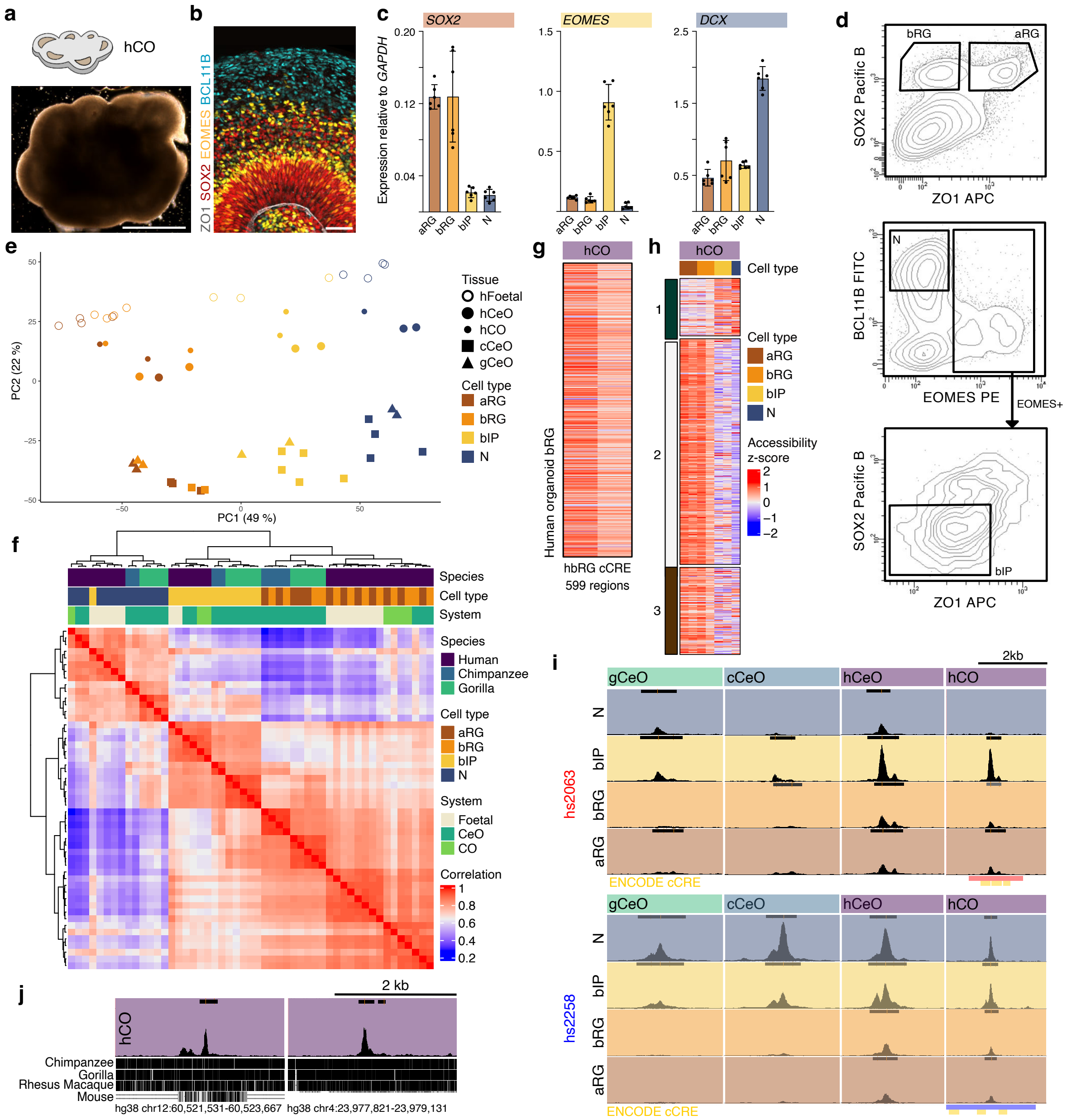

Supplementary Figure 4. ATAC-seq of two independent iPSC-line-derived human cortical organoids recapitulate accessibility patterns. a, Brightfield image of a whole sliced human CO. Scale bar, 1 mm. **b**, Immunohistochemistry for ZO1, SOX2, EOMES, and BCL11B of human CO. Scale bar, 50 µm. **c**, RT-qPCR of the canonical radial glia marker gene *SOX2*, the bIP marker gene *EOMES*, and the neuronal gene *DCX*, normalised to the housekeeping gene *GAPDH*, in sorted cell populations, confirms cell identity on the RNA level. Error bars represent standard deviation. **d**, FACS plots of human CO. Top: Separation of aRG and bRG based on expression of ZO1. SOX2 is expressed in both RG populations. Both cell populations are further sub-gated for DiI to select cells with basal processes. Middle: Gating for neurons was based on BCL11B+/EOMES–. Gating for bIPs was based on EOMES+ (middle) and SOX2–/ZO1– (bottom). **e**, Principal component analysis of the 1,000 most variable regions shows a separation of cell types on PC1 (49%) and species on PC2 (22%) after inclusion of additional human cell lines. **f**, Correlation heatmap of chromatin accessibility for all called

peaks across all tissues and cell types, indicating patterns of cell type- and species-specific accessibility. **g**, Accessibility z-scores of the 599 hbRG cCRE from (Fig. 1b) in the two replicates of human CO bRG. **h**, K-means clustering of the 599 hbRG cCRE reveals conserved three broad patterns of accessibility across human CO samples and cell types. **i**, Cell type-specific chromatin accessibility at two characterised VISTA enhancers, hs2063 (red; top) and hs2258 (blue; bottom), also marked by ENCODE cCRE signatures (yellow). Increased activity in human NPCs compared to gorilla and chimpanzee NPCs can be observed in both regions. hs2063 was previously shown to drive reporter gene expression in the central nervous system of the mouse at embryonic day 11.5, while hs2258 did not show activity at this time point. Shown are merged bigWig tracks, with standardised track height 0–4.5. Called peaks are represented by black bars in tracks. **j**, Two examples of human accelerated regions that are found among the hbRG cCRE. Shown are merged bigWig tracks of two replicates from human CO, with standardised track height 0–3. Called peaks are represented by black bars in tracks. Cactus evolutionary sequence conservation and chromosome coordinates are indicated. Source data are provided as a Source Data file.

Supplementary Figure 5

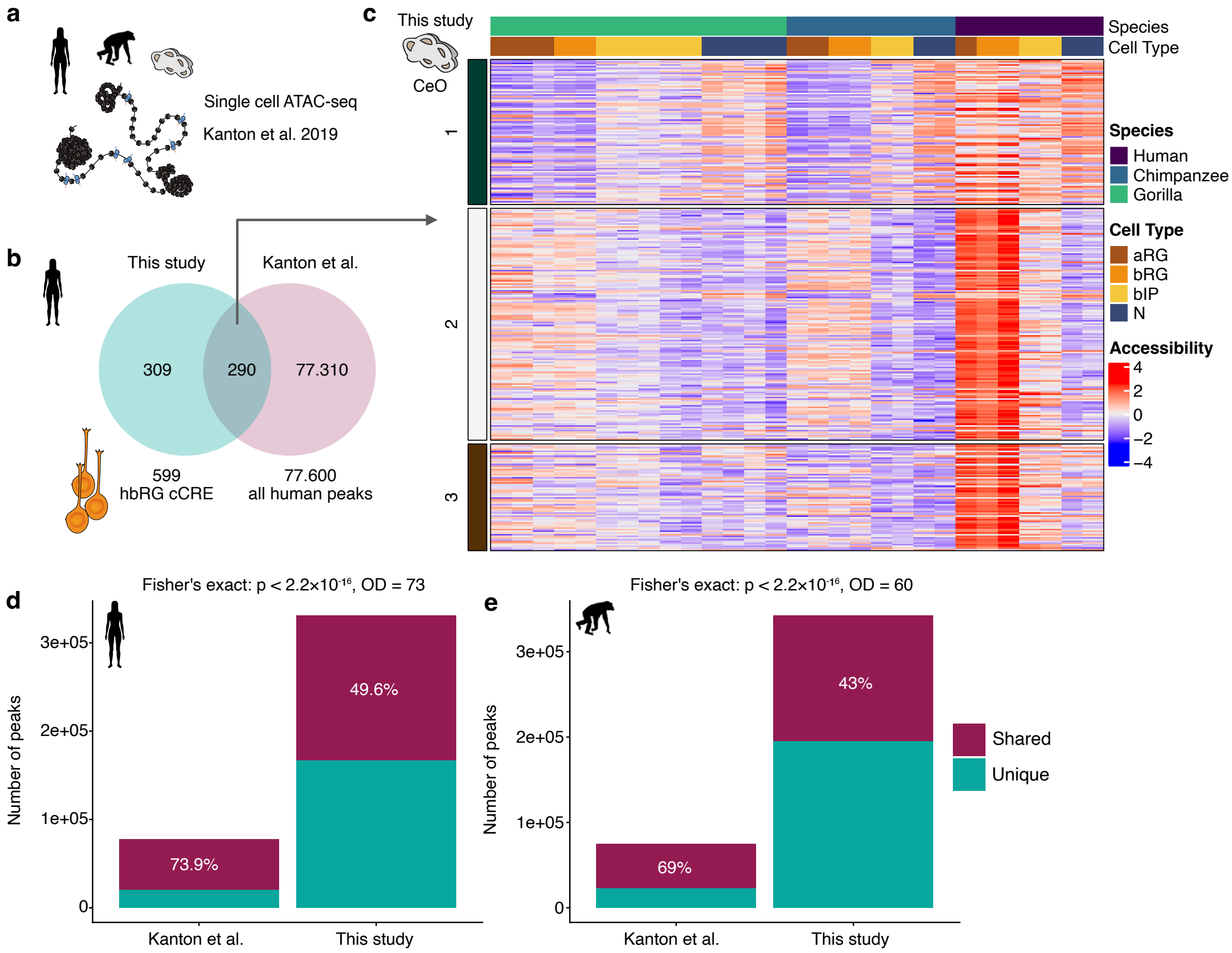

Supplementary Figure 5. Analysis of published human and chimpanzee CeO scATAC-seq shows high concordance between datasets. a, Published scATAC-seq of CeOs derived from six human and three chimp iPSC lines[4] was used to cross-validate our dataset. **b**, Of the 599 hbRG cCREs identified in our dataset, 290 overlapped peaks in human CeOs from Kanton et al. 2019, but only 122 overlapped peaks in chimpanzee cCeOs from Kanton et al. 2019. This corresponds to a ~2.4-fold human-to- chimpanzee peak ratio in the Kanton et al. 2019 dataset, closely matching the ~2.7-fold ratio observed in our dataset (221/599 chimpanzee peaks). **c**, The subset list of 290 hbRG cCREs found in both our and Kanton et al. 2019 dataset showed conserved accessibility patterns in three broad clusters across cell types as described in (Fig 2c). **d**, Across the entire 500 bp centered peak sets, 57,369/77,600 (73.9%) of hCeO Kanton et al. 2019 peaks overlapped our data, while 164,262/330,888 (49.6%) of our peaks overlapped Kanton et al. 2019 peaks (Jaccard similarity = 0.267; Fisher's exact test, ENCODE cCRE background, $p < 2.2\times10^{-16}$, odds ratio = 73). In chimpanzee, 51,625/74,502 (69%) of Kanton et al. 2019 peaks overlapped our data, while 147,535/342,770 (43%) of our peaks overlapped Kanton et al. 2019 peaks (Jaccard similarity = 0. 237, Fisher's exact test, ENCODE cCRE background, $p < 2.2\times10^{-16}$, odds ratio = 60). To further control for the non-uniform distribution of accessible chromatin, we performed 10,000 permutations of peaks across ENCODE cCREs. In both species, the observed overlaps greatly exceeded random expectation (human: 68,839 observed vs 16,517 expected overlaps; chimpanzee: 65,295 observed vs 15,830 expected overlaps; permutation $p < 0.0001$).

Supplementary Figure 6

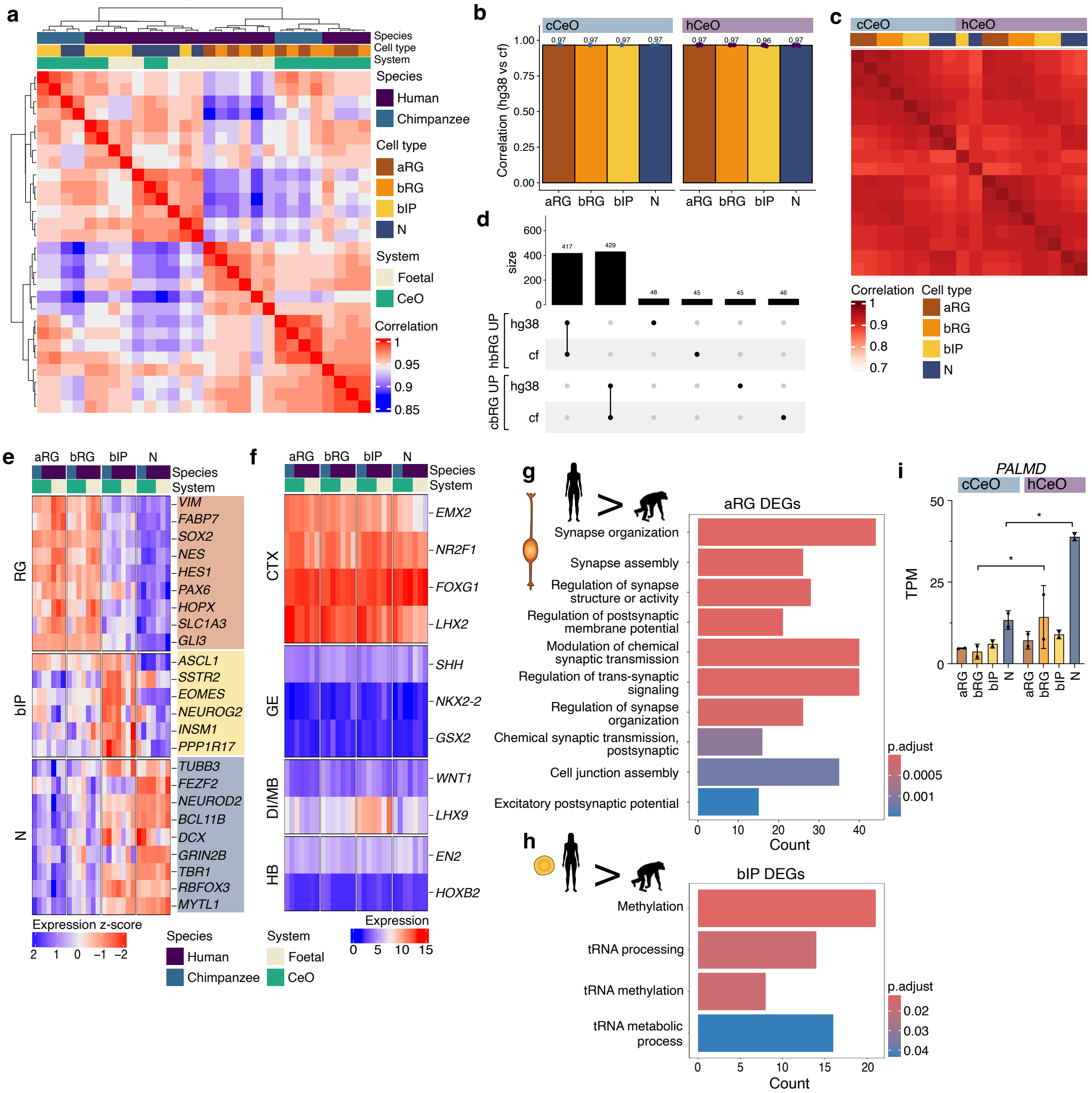

Supplementary Figure 6. RNA-seq reveals cell type- and species-specific expression patterns and confirms tissue identity. a, Correlation heatmap of all gene expression patterns reveals cell type and species-specific expression patterns. **b**, Mean per-sample correlation of cell type–specific expression profiles in human and chimpanzee cerebral organoids (hCeO, cCeO) comparing hg38-based and species-specific genome quantification (CrossFilt; cf). **c**, Heatmap of Pearson correlations between cf- and hg38-quantified rlog expression profiles across shared genes. Each cell shows the correlation between one sample quantified with cf and one sample quantified with hg38; diagonal entries correspond to the same biological sample quantified with the two annotations. **d**, UpSet plot showing overlap of differentially expressed genes across comparison groups. Each set represents the genes identified in one differential expression analysis category listed on the x-axis. Vertical bars indicate the number of genes in each intersection, and connected dots below the bars indicate the set combination contributing to that intersection. **e**, Expression z-score of canonical radial glia, bIP and neuronal marker genes confirms cell type identity. RG, radial glia; bIP, basal intermediate progenitor;

N, neuron. **f**, Absolute expression of brain region-specific genes across chimpanzee and human CeOs and human foetal tissue confirms tissue identity at the transcriptional level. CTX, cortex; GE, ganglionic eminence; DI/MB, diencephalon/midbrain; HB, hindbrain; DEG, differentially expressed gene. **g**, GO term analysis of genes expressed at higher levels in human vs. chimpanzee aRG indicates enrichment in terms related to regulating synapse structure, but not those regulating other cell processes/protrusions seen in human bRG upregulated genes. Top 10 terms displayed with $p \leq 0.05$. **h**, GO terms of genes expressed at higher levels in human vs. chimpanzee bIP categorise as regulating several processes related to transfer RNAs. All significant terms are displayed with $p \leq 0.05$. **i**, Gene expression of *PALMD* determined by RNA-seq. Mean ± SD displayed in TPM. *, DESeq2, logFC ≥ 1.5; $p \leq 0.05$. Source data are provided as a Source Data file.

Supplementary Figure 7

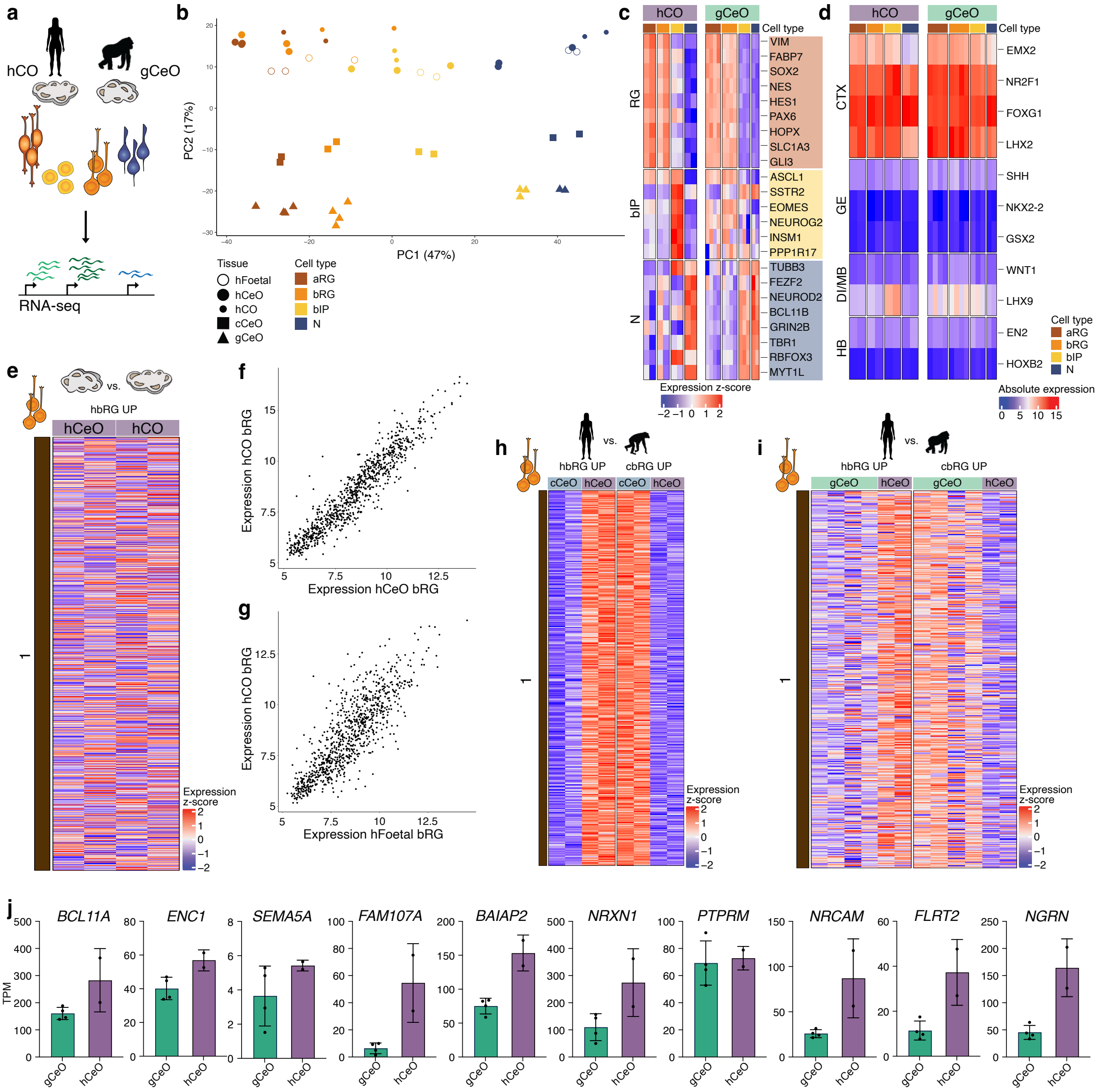

Supplementary Figure 7. Expression of human bRG upregulated genes in human COs and gorilla CeOs corroborates human-upregulated expression. a, RNA-seq analysis of cell types sorted from human COs and gorilla CeOs. **b**, Principal Component analysis of 500 most variable genes shows a separation of cell type on PC1 (47%) and species on PC2 (17%). **c**, Expression z-score of canonical radial glia, bIP and neuronal marker genes confirms cell type identity for both human CO and gorilla CeO. **d**, Absolute expression of brain region-specific genes across human CO and gorilla CeO confirms tissue identity at the transcriptional level. **e**, Expression z-score of the 465 human bRG upregulated genes in hCO and hCeO shows highly comparable patterns. **f**, **g** Expression of the 465 human bRG upregulated genes is highly correlated between organoid protocols (**f**) and human CO and foetal tissue (**g**). **h**, Expression z-score of 465 human bRG upregulated genes in cCeO and hCeO bRG (left) and 474 chimpanzee bRG upregulated genes in cCeO and hCeO bRG (right). **i**, Expression z-score of 465 human bRG upregulated genes in gCeO and hCeO bRG (left) and 474 chimpanzee bRG upregulated genes in gCeO and hCeO bRG (right). **j**, Morphoregulatory candidate genes

upregulated in human compared to chimpanzee bRG (Fig. 3e) show a pattern of generally conserved human bRG upregulation when compared to gorilla CeO bRG. Source data are provided as a Source Data file.

Supplementary Figure 8

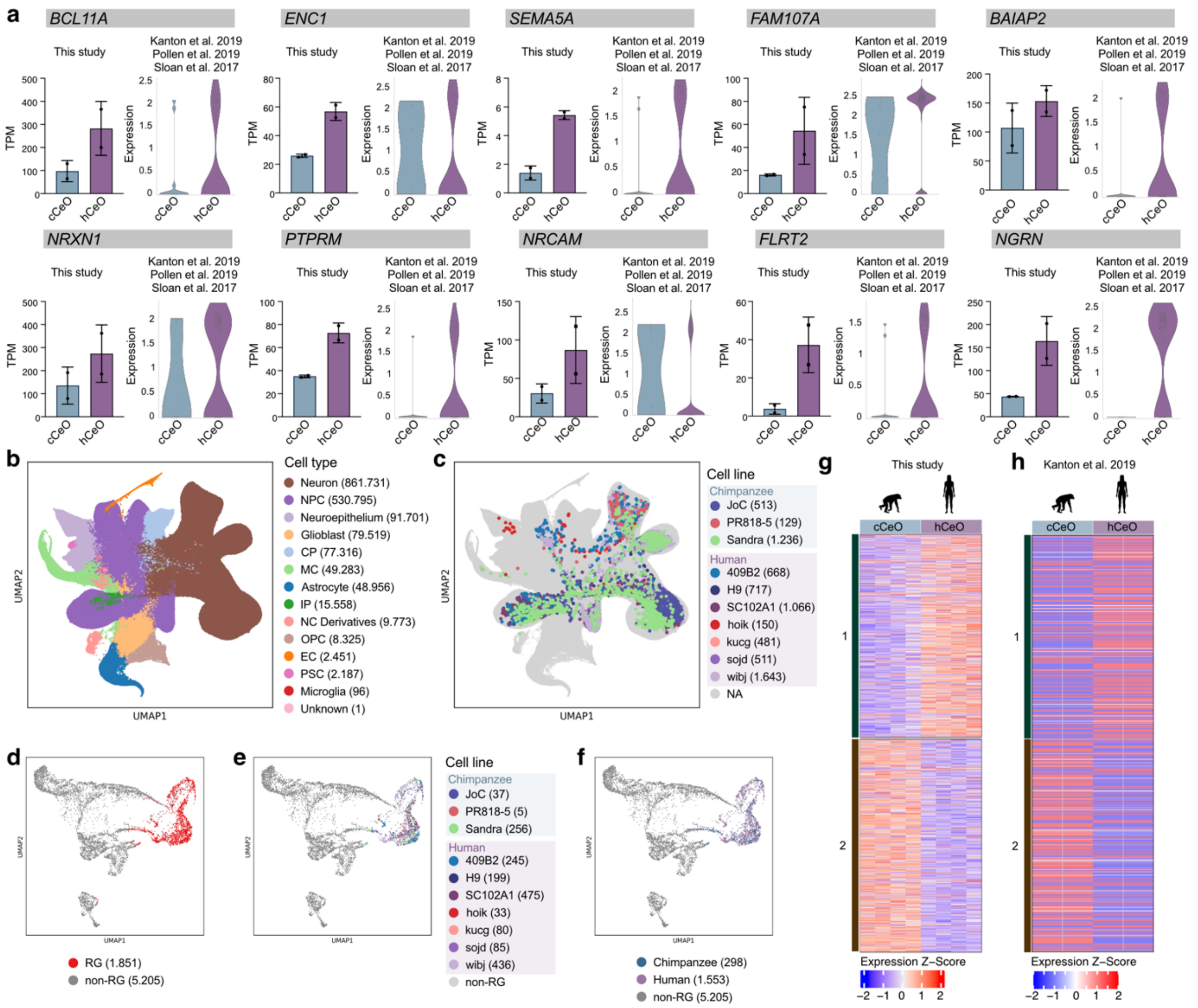

Supplementary Figure 8. Published scRNA-seq data reveals comparable expression patterns of human bRG upregulated genes across cell lines and organoid stages. a, Morphoregulatory candidate genes upregulated in human bRG (Fig. 3e) show a pattern of generally conserved human bRG upregulation in our data and published comparative scRNA-seq datasets[4,7,8]. Data was accessed and violin plots were generated using gEAR[9] **b**, UMAP embedding of the query datasets together with the HNOCA reference dataset[10], showing 14 cell identities predicted using ArchMap: Neuron; NPC, neural progenitor cell; Neuroepithelium; Glioblast; CP, choroid plexus; MC, mesenchymal cell; Astrocyte; IP, intermediate progenitor; NC Derivatives; OPC, oligodendrocyte precursor cell; EC, endothelial cell; PSC, pluripotent stem cell; Microglia; and Unknown. **c**, The same UMAP embedding coloured by cell line (3 chimpanzee and 7 human iPSC) after filtering cells based on organoid age. **d–f**, UMAP embedding of the original datasets[4], highlighting cells identified as radial glia (**d**), based on cell line (**e**) and species (**f**). **g, h,** Expression z-score of grouped-RG differentially expressed genes identified in our chimpanzee and human CeO (**g**; age d56±3) shows comparable expression patterns to Kanton et al. 2019 chimpanzee and human CeO (**h**; age day 60–74). Source data are provided as a Source Data file.

Supplementary Figure 9

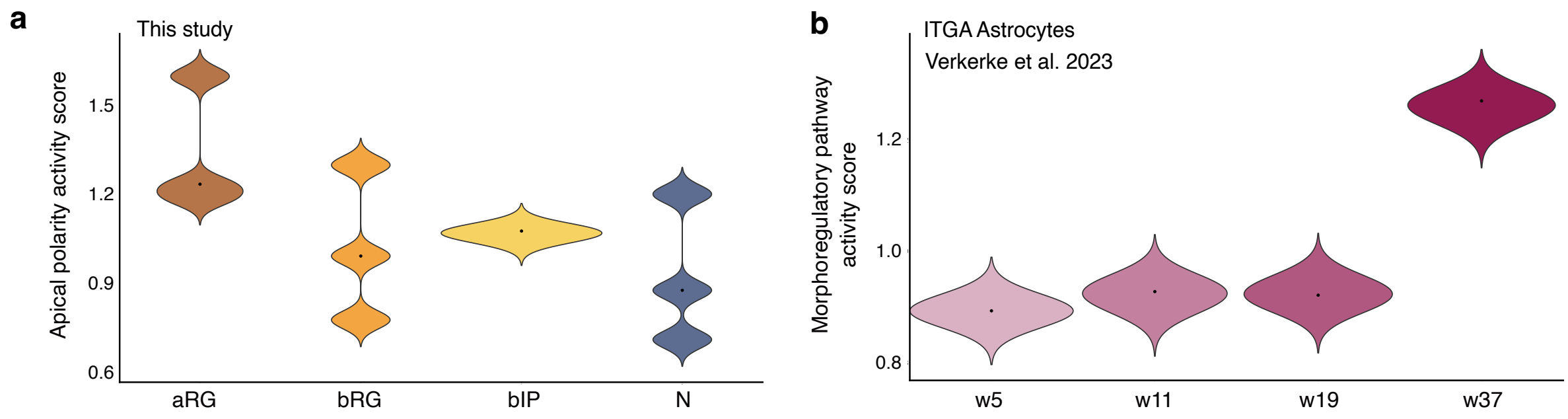

Supplementary Figure 9. Validation of morphoregulatory pathway activity score in the context of the developing cortex. a, Apical polarity pathway activity score, adapted from [11] and calculated based on GO term gene groups 'Apical junction assembly', 'Establishment of epithelial cell apical basal polarity' and 'Regulation of adherens junction organisation', indicates higher activity in human foetal aRG compared to other isolated cell types. **b**, Morphoregulatory pathway activity score increased with cellular maturation and complexity in published human CeO-derived astrocytes[12].

Supplementary Figure 10

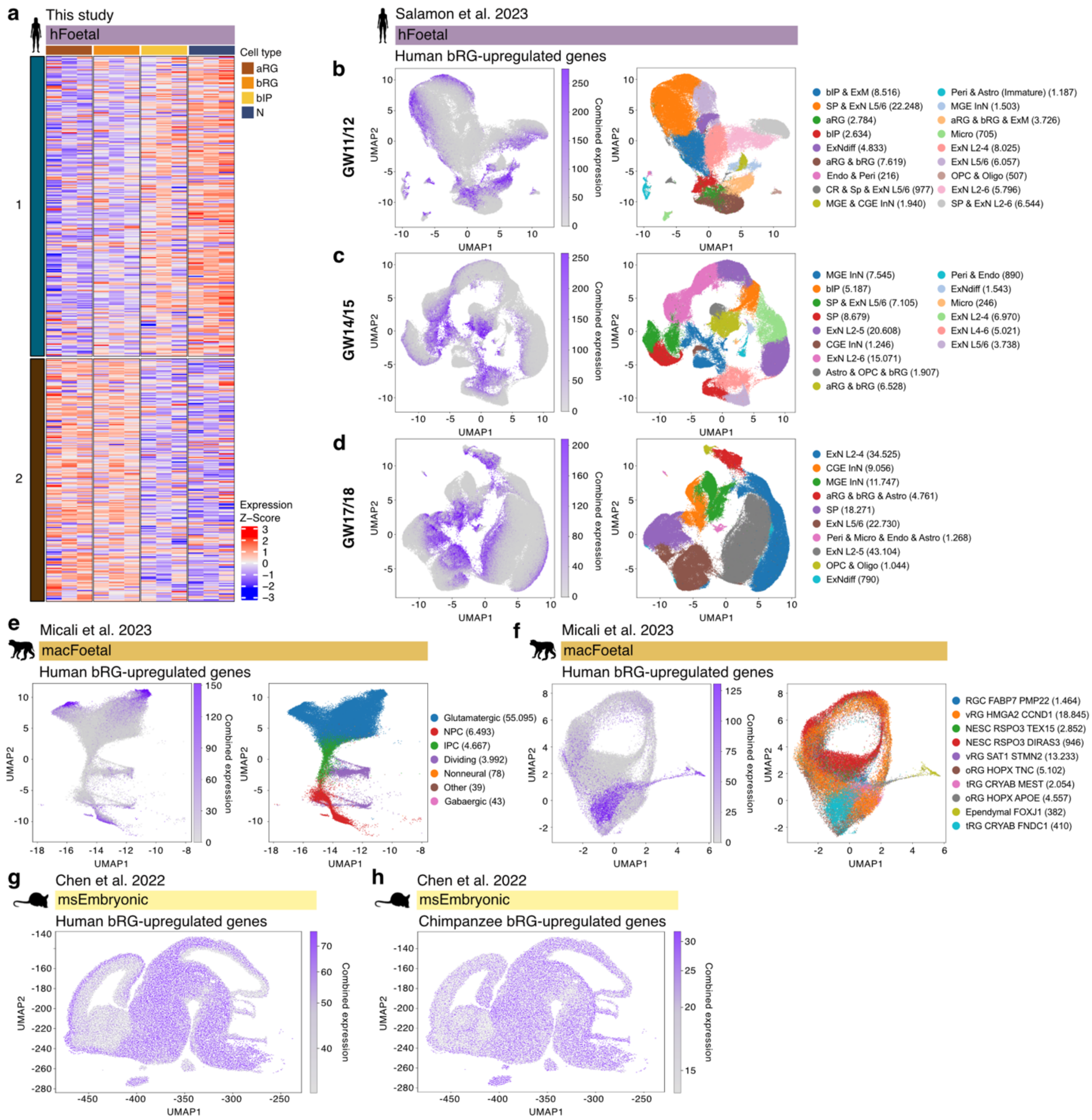

Supplementary Figure 10. Human bRG upregulated genes show a dual expression pattern in radial glia and neuronal populations across development and species. a, Expression z-score of 465 human bRG upregulated genes in human foetal cells reveals segregation in two broad clusters (k-means) with cluster 1 showing relatively higher expression in neurons and cluster 2 showing relatively higher expression in radial glia. **b–d**, UMAP embeddings showing unweighted, z-score gene expression of 465 human bRG upregulated genes (of which 428 genes are detected in the dataset) using the projection tool in gEAR[9] of GW11–12 (**b**), GW14–15 (**c**), and GW17–18 (**d**) snRNA-seq data from developing human cortex[13]. This reveals a similar, consistent pattern across cell types and developmental stages with strongest expression in aRG/bRG clusters as well as in excitatory and inhibitory neurons. Notably, expression is not restricted to these lineages but is also evident in pericytes, microglia, endothelial cells, and astrocytes. SP, Subplate neurons; aRG, apical radial glia; bRG, basal radial glia; CR cells, Cajal-Retzius cells; ExN L2-4, layer 2–4 excitatory neurons; ExN L5-6,

layer 5/6 excitatory neurons; ExN_{diff}, differentiating excitatory neurons; OPC, oligodendrocyte precursor cells; Astro, astrocytes; MGE InN, medial ganglionic eminence-derived interneurons; CGE InN, caudal ganglionic eminence-derived interneurons; Endo, endothelial cells; Peri, pericyte; Micro, microglia. **e, f**, UMAP embeddings of foetal macaque telencephalon[14] showing unweighted, z-score gene expression of 465 human bRG upregulated genes (of which 421 genes are detected in the dataset). Human bRG upregulated genes are primarily expressed in neurons and NPCs (**e**) and within radial glia are enriched in bRG, labelled as oRG and tRG (**f**). RGC, radial glia cell; vRG, ventral radial glia; NESC, neuroepithelial stem cell; oRG, outer radial glia; tRG, truncated radial glia. **g, h**, Mouse E16.5 developing cortex analysed by spatial transcriptomics using DNA nanoball-patterned arrays (MOSTA)[15]. UMAP embeddings showing unweighted, z-score gene expression of 465 human bRG upregulated genes (of which 461 genes are detected in the dataset) reveal enriched expression in the cortical plate (**g**); a pattern not observed for the 474 chimpanzee bRG upregulated genes (of which 471 genes are detected in the dataset) (**h**).

Supplementary Figure 11

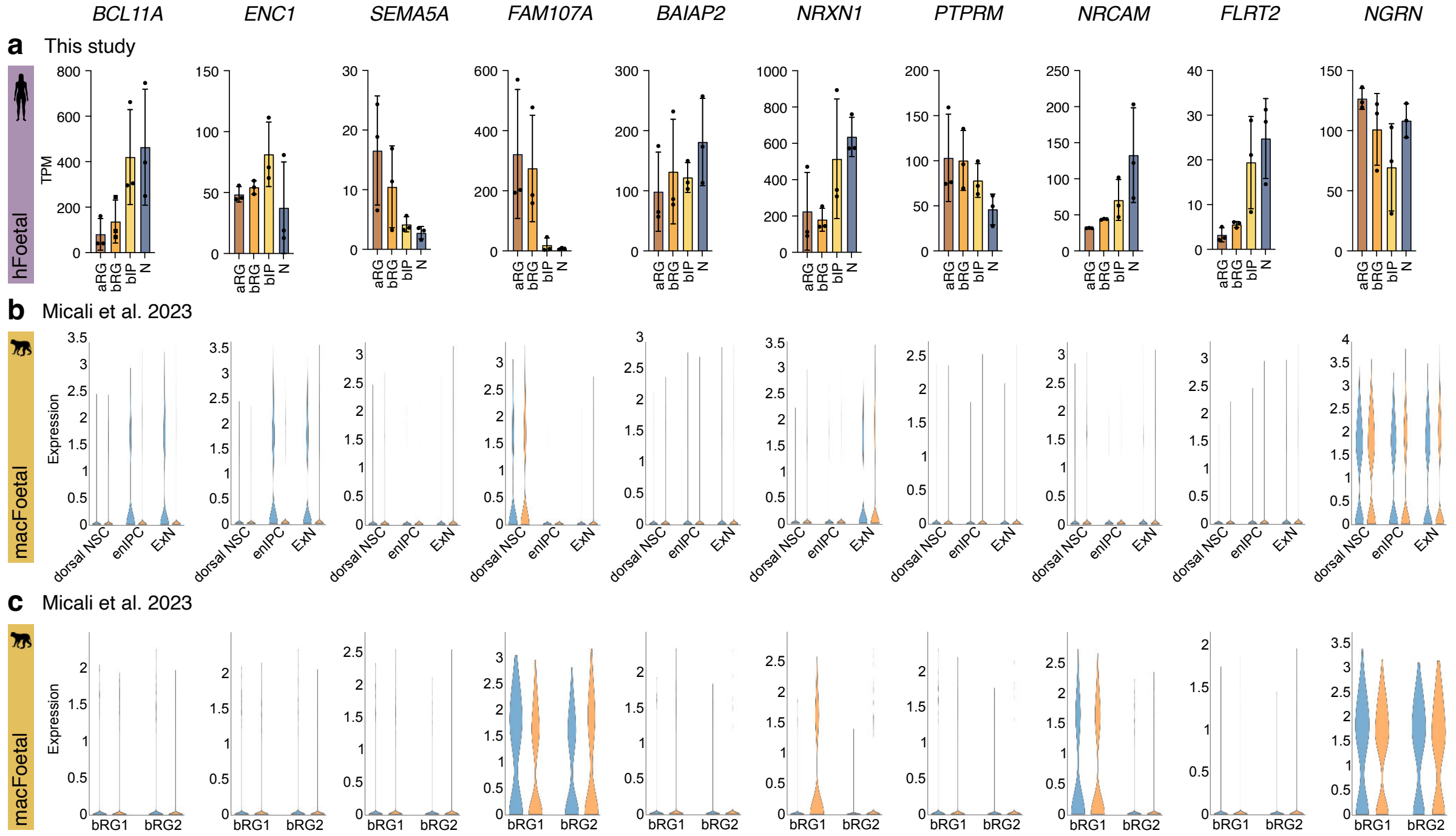

Supplementary Figure 11. Morphoregulatory candidate gene expression in macaque foetal telencephalon corroborates human bRG upregulated expression. a, Gene expression of *BCL11A, ENC1, NRXN1, BAIAP2, NRCAM, FLRT2, NGRN, FAM107A, SEMA5A* and *PTPRM* determined by RNA-seq in human foetal tissue (this study). Mean ± SD displayed in TPM. **b, c**, Expression of the same genes in published scRNA-seq data of macaque foetal telencephalon[14] in dorsal NSC, enIPC and ExN (**b**), and bRG (**c**), accessed and visualized via gEAR[9]. Dorsal NSC, dorsal neural stem cell; enIPC, excitatory neurogenesis intermediate progenitor cells; ExN, excitatory neurogenesis neurons; E, embryonic day. Source data are provided as a Source Data file.

Supplementary Figure 12

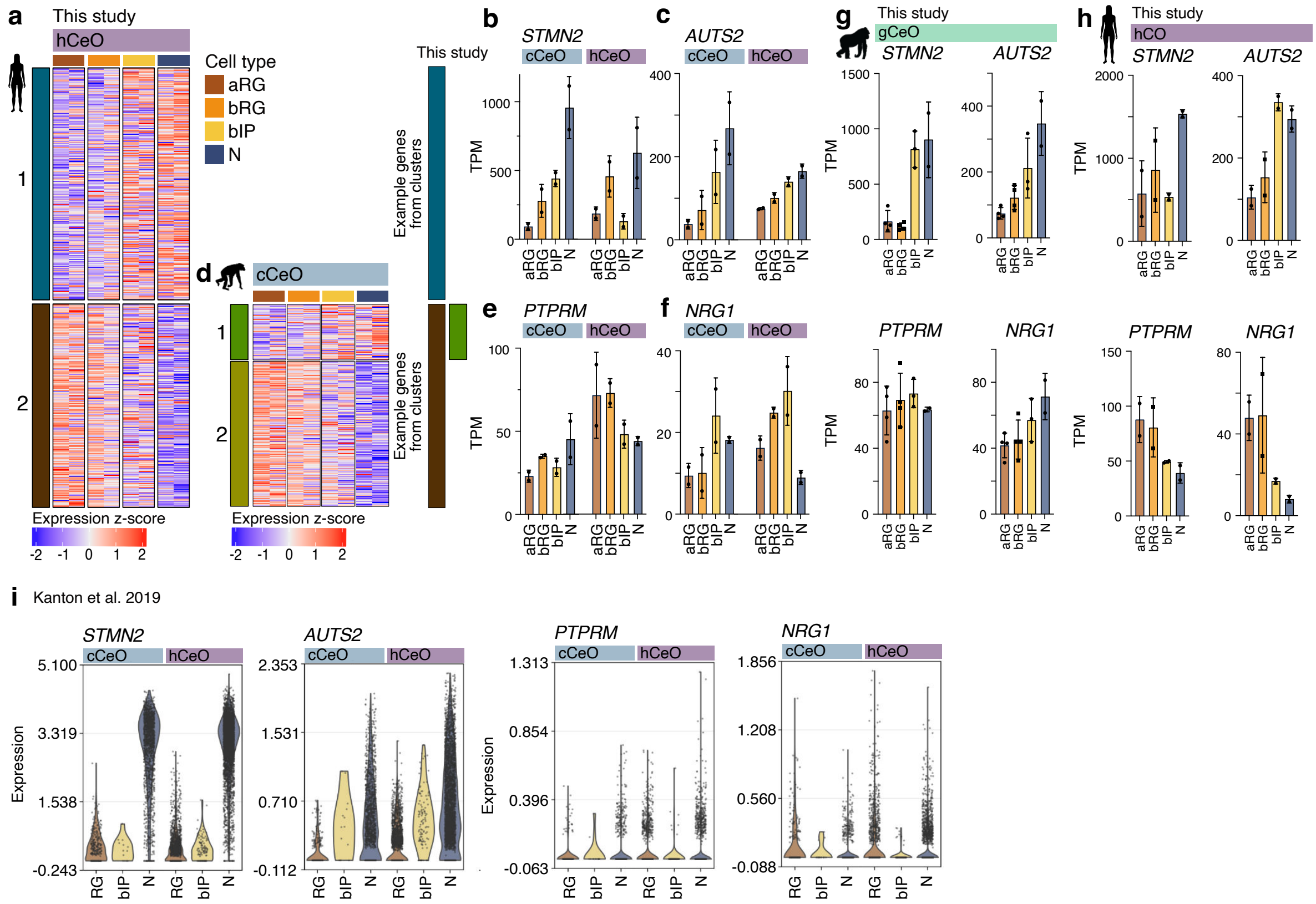

Supplementary Figure 12. Clustering of hbRG cCRE target genes by gene expression. a, RNA expression of hbRG cCRE target genes in hCeO reveals two clusters of expression (k-means) across all cell types. Z-score is shown. **b**, **c**, *STMN2* and *AUTS2* are two examples of genes in cluster 1, showing higher expression in neurons than neural progenitor cells. Mean ± SD displayed in TPM. **d**, RNA expression of cluster 2 hbRG cCRE target genes in cCeO shows broadly conserved expression patterns but reveals a subset of genes more highly expressed in bIP and neurons (chimpanzee cluster 1). Z-score is shown. **e**, **f**, *PTPRM* and *NRG1* are two examples of genes in human cluster 2 and chimpanzee cluster 1 showing ancestral neuronal expression and significantly increased expression in human neural progenitor cell types. **g**, RNA expression of *STMN2*, *AUTS2*, *PTPRM* and *NRG1* in gorilla CeO. **h**, RNA expression of *STMN2*, *AUTS2*, *PTPRM* and *NRG1* in human CO. **i**, Expression patterns of *STMN2*, *AUTS2*, *PTPRM* and *NRG1* in published scRNA-seq of human and chimpanzee CeO[4]. TPM, transcripts per million. Source data are provided as a Source Data file.

Supplementary Figure 13

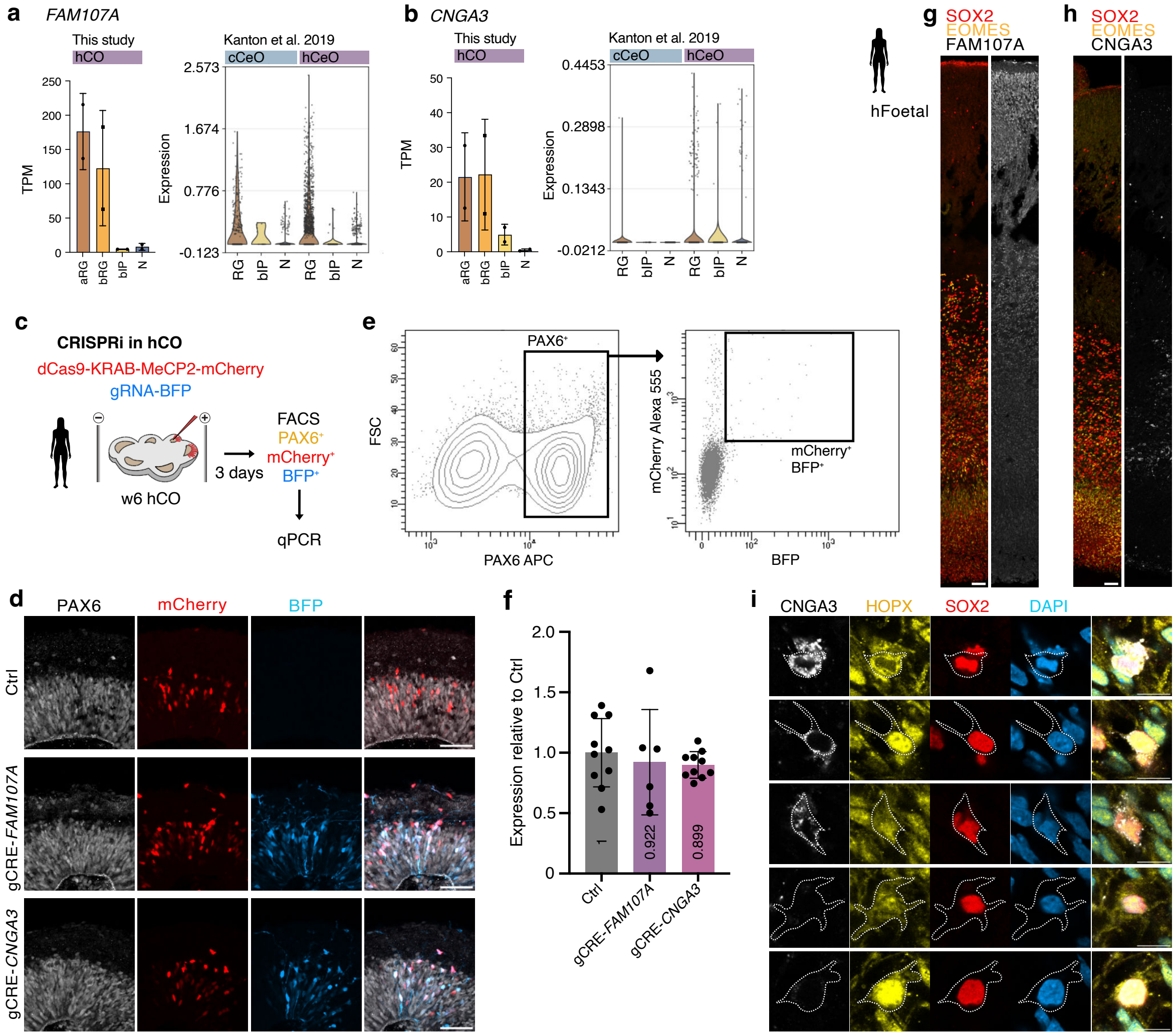

Supplementary Figure 13. *FAM107A* and *CNGA3* show human radial glia–enriched expression and are regulated by distal cis-regulatory elements in hCOs. a, b, Gene expression of *FAM107A* (**a**) and *CNGA3* (**b**) in hCO (this study) and published scRNA-seq[4] of hCeO and cCEO, showing increased expression in human radial glia, as also seen in hCeO of this study (Fig. 4a, b). **c**, dCas9-KRAB-MeCP2 mediated CRISPRi targeting of *FAM107A* and *CNGA3* cCREs was performed in hCOs. Three days after electroporation, PAX6+ mScarlet+ BFP+ cells were sorted for RT-qPCR analysis. **d**, Immunohistochemistry for PAX6 and RFP (detecting mCherry) and native BFP signal in electroporated hCO. **e**, PAX6+ radial glia with dCas9-mScarlet and gRNA-BFP expression were isolated by FACS. **f**, RT-qPCR was used to quantify target gene expression compared to a non-targeting control. Mean ± SD normalised to control are shown for 10 (Ctrl), 6 (*FAM107A*) and 10 (*CNGA3*) individual samples. **g**, Immunohistochemistry for SOX2, EOMES and FAM107A in human foetal cortex at GW16. **h**, Immunohistochemistry for SOX2, EOMES and CNGA3 in human foetal cortex at GW16. **i**, Immunohistochemistry for CNGA3, HOPX, and SOX2 and staining for DAPI in human foetal cortex at GW16. Cells with bRG marker expression in the SVZ do not show a consistent association of CNGA3 levels and morphological complexity. Source data are provided as a Source Data file.

Supplementary Figure 14

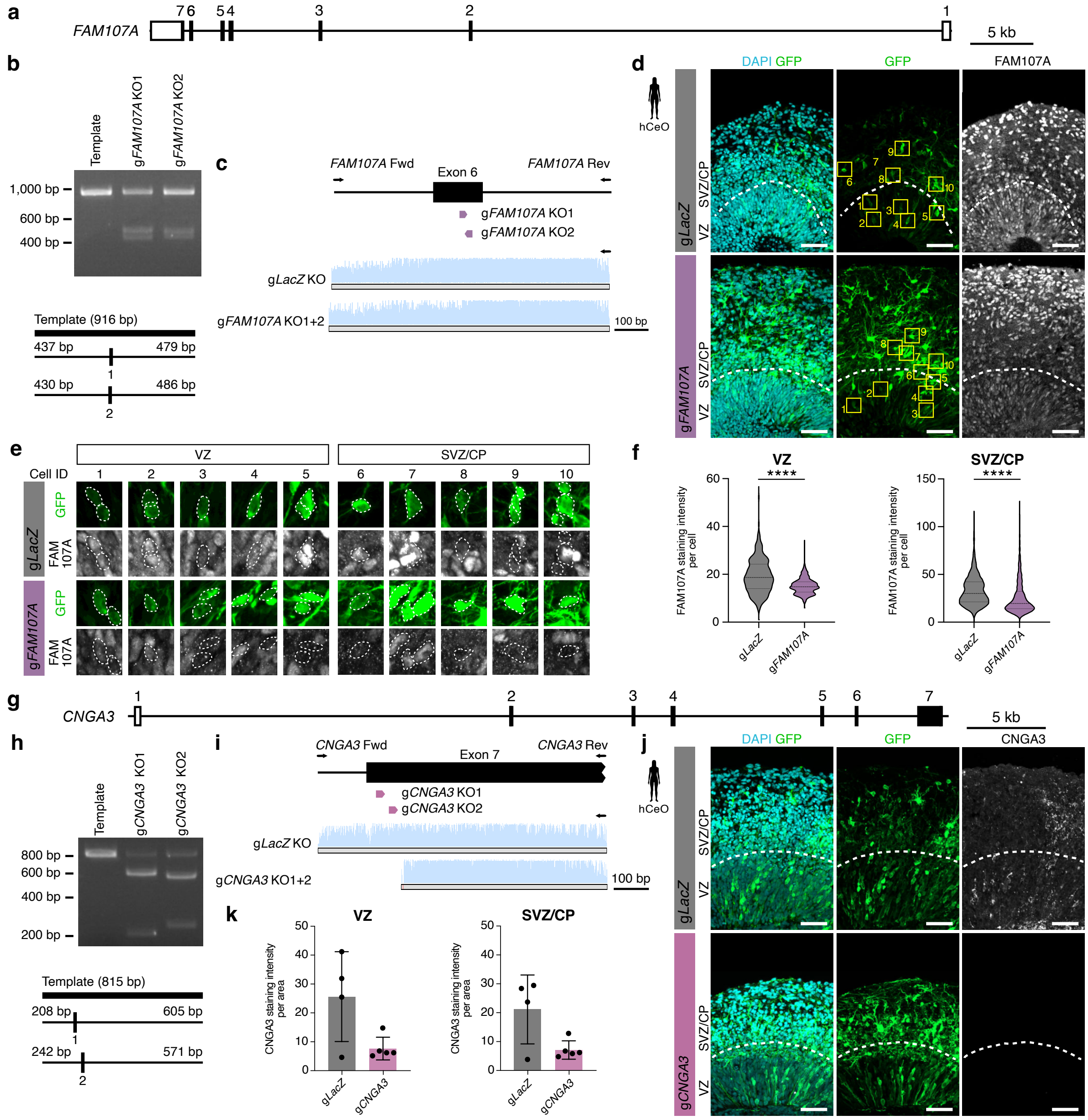

Supplementary Figure 14. CRISPR/Cas9-mediated knockout of *FAM107A* and *CNGA3*. **a**, Schematic illustration of the human *FAM107A* gene locus. **b**, Guide RNA efficiencies were tested *in vitro*. The effects of the g*FAM107A* KO1 and KO2 RNAs to direct Cas9-mediated cutting of PCR templates were analysed by agarose gel electrophoresis. Schemes of the sizes of PCR templates, guide RNA binding sites, and expected sizes of cut fragments are indicated below. **c**, CRISPR/Cas9-mediated targeting of *FAM107A* was confirmed in the CRTDi004-A iPSC line by electroporation of Cas9/gRNA ribonucleoprotein complexes together with a GFP plasmid, followed by FACS of GFP-positive cells, PCR amplification of the target region and Sanger sequencing. The sequencing results are shown for combined g*FAM107A* KO1+2. **d**, Immunohistochemistry for GFP and FAM107A and staining for DAPI in control and KO organoids. **e**, Zoomed in images of the regions highlighted in (d) showing GFP+ electroporated cells in the VZ and SVZ/CP at higher magnification. Dotted lines outline the cell body. **f**, Quantification of FAM107A staining intensity in the ventricular zone (VZ) and subventricular

zone/cortical plate (SVZ/CP) of g*LacZ* control and g*FAM107A* KO organoids. **g**, Schematic illustration of the human *CNGA3* gene locus. **h**, The effects of the g*CNGA3* KO1 and KO2 RNAs to direct Cas9-mediated cutting of PCR templates were analysed by agarose gel electrophoresis. **i**, CRISPR/Cas9-mediated targeting of *CNGA3* was confirmed in iPSC. The sequencing results are shown for combined g*CNGA3* KO1+2. **j**, Immunohistochemistry for GFP and CNGA3 and staining for DAPI in control and KO organoids. **k**, Quantification of CNGA3 staining intensity in the ventricular zone (VZ) and subventricular zone/cortical plate (SVZ/CP) of g*LacZ* control and g*CNGA3* KO organoids. **** $p < 0.0001$, Mann-Whitney-U test. Scale bars, 50 µm. Source data are provided as a Source Data file.

Supplementary Figure 15

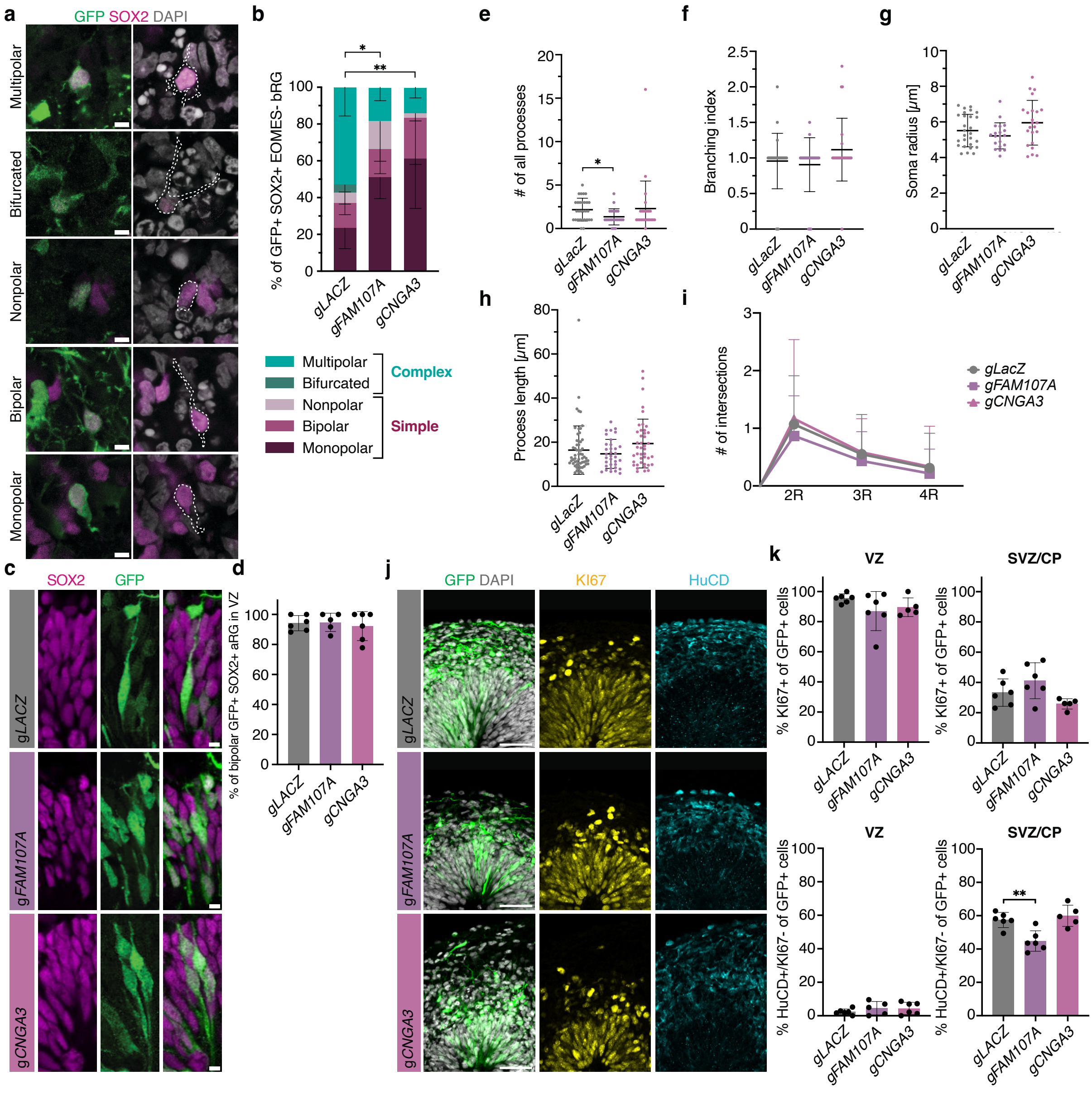

Supplementary Figure 15. FAM107A and CNGA3 alter bRG morphotype distribution, with selective effects of FAM107A on morphology and neurogenesis. a, Representative images of bRG morphotypes, as previously described[16]. Immunofluorescence for GFP and SOX2, and staining for DAPI. Scale bars, 5 µm. **b**, Distribution of bRG morphotypes. Mean of 3 independent hCO differentiation and KO experiments. Total number of cells scored: 41 (g*LacZ*; Control), 28 (g*FAM107A*), and 36 (g*CNGA3*). Error bars represent SD; ** $p < 0.01$, * $p < 0.05$; 2-way ANOVA with Bonferroni post hoc test. Note the absence of bifurcated cells upon *FAM107A* and *CNGA3* knockout. **c**, Representative images of apical radial glia identified as GFP+ SOX2+ cells in the ventricular zone were analysed for morphology. Scale bars, 5 µm. **d**, Quantification of the proportion of bipolar aRG upon targeting with g*LacZ*, g*FAM107A* or g*CNGA3* CRISPR/Cas9. The proportions are not statistically significant; Welch's t-test. **e–i**, Quantifications of morphology parameters of SOX2+/EOMES– bRG in the subventricular zone of g*LacZ*, g*FAM107A* and g*CNGA3* CRISPR/Cas9 targeted hCOs, specifically the number of total processes (**e**), branching index (**f**), soma radius in µm (**g**), process length in µm

(**h**) and Scholl analysis as a measure of arborization (**i**). * $p < 0.05$; non-labelled comparisons are not statistically significant; Mann-Whitney U-test (**e**, **f**, **h**), Welch's t-test (**g**), 2-way ANOVA with Bonferroni post hoc test (**i**). **j**, Immunofluorescence for GFP, KI67 and HuCD, and staining for DAPI of g*LacZ*, g*FAM107A* and g*CNGA3* CRISPR/Cas9 targeted hCOs. Scale bar, 50 µm. **k**, Quantification of the percentage of proliferating KI67+ cells of GFP+ cells in the ventricular zone (VZ) and subventricular zone/cortical plate (SVZ/CP) (top), and of HuCD+/Ki67– neurons (bottom) in g*LacZ*, g*FAM107A* and g*CNGA3* CRISPR/Cas9 targeted hCOs. Note the significant reduction of neurons upon loss of *FAM107A*. ** $p < 0.01$, Student's T-test. Source data are provided as a Source Data file.

Supplementary Figure 16

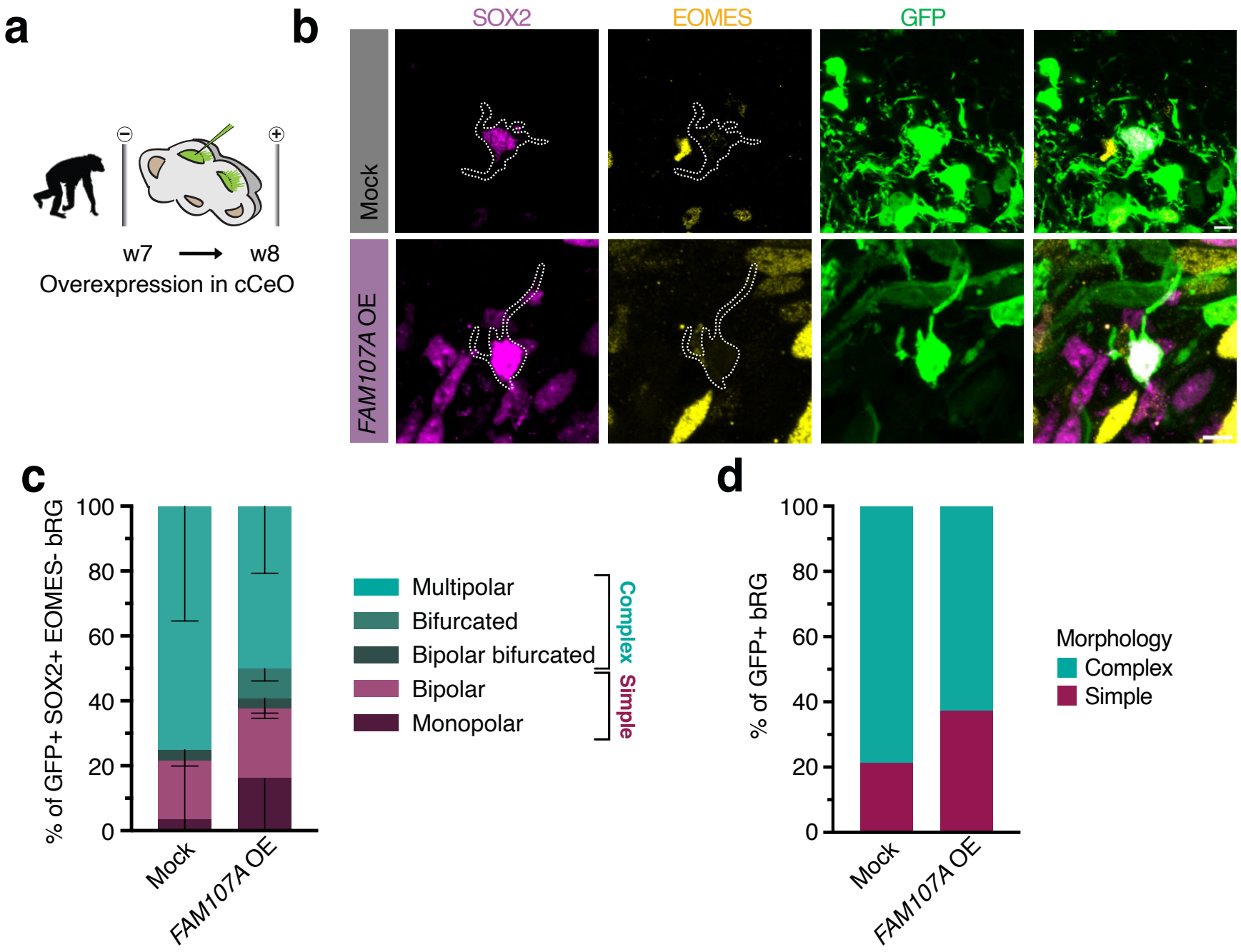

Supplementary Figure 16. Overexpression of FAM107A in chimpanzee CeO.
a, Scheme of the overexpression of human *FAM107A* in chimpanzee CeOs by electroporation of a CAG overexpression plasmid. **b**, Examples of electroporated GFP+ SOX2+ EOMES– bRG in the subventricular zone of electroporated cCeOs revealed by immunofluorescence for GFP, SOX2 and EOMES, along with the relative cell mask. Scale bars, 5 µm. **c**, Quantification of the relative distribution of GFP+ SOX2+ EOMES– bRG according to their morphological complexity[16,17]. Mean of two independent chimpanzee CeO differentiation and overexpression experiments. Total number of cells scored: 24 (Ctrl) and 49 (*FAM107A* OE). Error bars represent SD. **d**, Quantification of the relative distribution of bRG according to either complex or simple morphotype. Source data are provided as a Source Data file.

Supplementary Information Table 1. Oligonucleotides.

Oligo	Species	Sequence	Reference
Gene expression analysis by qPCR			
GAPDH_F	human	5'-AAGGTGAAGGTCGGAGTCAA	[18]
GAPDH_R	human	5'-AATGAAGGGGTCATTGATGG	[18]
qSOX2_1F	human	5'-CCAAGATGCACAACTCGGAG	This paper
qSOX2_1R	human	5'-GCTTAGCCTCGTCGATGAAC	This paper
qEOMES_4F	human	5'-GAGCCCTCAAAGACCCAGAC	[18]
qEOMES_4R	human	5'-<br>TTGCAAAGGGGTTATGATCAATC	[18]
qNGN2_1F	human	5'-TGGTACACGATTGCAAACGG	[18]
qNGN2_1R	human	5'-CTACGGGTCTTCTTGATGCG	[18]
qDCX_1F	human	5'-TGTGTTTATTGCCTGTGGTCC	[18]
qDCX_1R	human	5'-CTGTGGCTGATGGGTTTCC	[18]
qTUBB3_1F	human	5'-AGCGTCTACTACAACGAGGC	[18]
qTUBB3_1R	human	5'-AAGAGATGTCCAAAGGCCCC	[18]
qGorillaEOMES_1F	gorilla	5'-GAGCCCTCAAAGACCCAGAG	This paper
RNA-seq			
dT-primer	N/A	C6-aminolinker-<br>AAGCAGTGGTATCAACGCAGAGT<br>CGAC<br>TTTTTTTTTTTTTTTTTTTTTTTTT<br>TTTTVN, N = random base; V = any<br>base beside thymidine	
TSO-primer	N/A	AAGCAGTGGTATCAACGCAGAGT<br>ACATrGrGrG, where rG stands for<br>ribo-guanosine	
UP-primer	N/A	AAGCAGTGGTATCAACGCAGAGT	
Luciferase Assay and Enhancer Activity Assay constructs			
hCNGA3_wt	human	ATGGTACCAAACTCACAAACAAA<br>GCAAGGGAAAAATGAAGCAACAA<br>AAGCAGGGATTTATTGAAAACGA<br>AAGTACCCTCCACAGGATGTGAG<br>CTGGCTGAGCATAGGGGCTCCAG<br>AGCCCAGTTACAGAATTTTCTGGT<br>GTTTAAACACCCTCTAGAGGTTTC<br>TCATTGGCCGCTTGATGTACACCC<br>CATGCAAATGAAGTAGTGGCCTG<br>CAGTCAGTCTGATTGCTAGCAT	This paper
hCNGA3_mut	human	ATGGTACCAAACTCACAAACAAA<br>GCAAGGGAAAAATGAAGCAACAA<br>AAGCAGGGATTTATTGAAAACGA<br>AAGTACCCTCCACAGGGTGTGAG<br>CTGGCTGAGCATAGGGGCTCCAG<br>AGCCCAGTTACAGAATTTTCTGGT<br>GTTTAAACACCCTCTAGAGGTTTC<br>TCATTGGCCGCTTGATGTACACCC<br>CATGCAAATGAAGTAGTGGCCTG<br>CAGTCAGTCTGATTGCTAGCAT	This paper
cCNGA3	chimpanzee	ATGGTACCAAACTCACAAACAAA<br>GCAAGGGAAAAATGAGGCAACAA<br>AAGCAGGGATTTATTGAAAACGA<br>AAGTACCCTCCACAGG**G**TGTGAG<br>CTGGCTGAGCATAGGGGCTCAAG<br>AGCCCAGTTACAGAATTTTCTGGT<br>GTTTAAACACCCTCTAGAGGTTTC	This paper

		TCATTGGCCACTTGATGTACACCC<br>CATGCAAATGAAGTAGTGGCCTG<br>CAGTCAGTCTGATTGCTAGCAT	
hFAM107A_wt	human	ATGGTACCGGTGCTTCTCTGCTAC<br>TGGAAGAAAGTCGTGTTTCTTTTT<br>CCCTTCTCATTTTCAGAAAAGAAG<br>TGTGAAAGGATCAAAAGAAAGGA<br>TTTGCTTAGATGTTAGTGTGGCCT<br>GATTAGGTCGGAATTAGCCTTTTT<br>CTCTTTTCACTGAGAGAAGCAGAG<br>ACCATCTGCGGAAATTATTTAAAT<br>TCATGCGCCTTCCAGGGAAGCTGT<br>TTTATTTGCGCTAGCAT	This paper
hFAM107A_mut	human	ATGGTACCGGTGCTTCTCTGCTAT<br>TGGAAGAAAGTCGTGTTTCTTTTT<br>CCCTTCTCATTTTCAGAAAAGAAG<br>TGTGAAAGGATCAAAAGAAAGGA<br>TTTGCTTAGATGTTAGTGTGGCCT<br>GATTAGGTCGGAATTAGCCTTTTT<br>CTCTTTTCACTGAGAGAAGCAGAG<br>ACCATCTGCGGAAATTATTTAAAT<br>TCATGCGCCTTCCAGGGAAGCTGT<br>TTTATTTGCGCTAGCAT	This paper
cFAM107A	chimpanzee	ATGGTACCGGTTCTTCTCTGCTAT<br>TGGAAGAAAGTCGTGTTTCTTTTT<br>CCCTTCTCATTTTCAGAAAAGAAG<br>TGTGAAAGGATCAAAAGAAAGGA<br>TTTGCTTAGATGTTAGTGTGGCCT<br>GATTAGGTCGGAATTAGCCTTTTT<br>CTCTTTTCACTGAGAGAAGCAGAG<br>ACCATCTGCGGAAATTATTTAAAT<br>TCATGTGCCTTCCAGGGAGGCTGT<br>TTTATTTGCGCTAGCAT	This paper
Scr286		TGCCAGAACATTTCTCTGGCCTAA<br>CTGGCCAGGACCGGATCAACTAG<br>GACCGGATCAACTTCCATCGACGC<br>GTCTAAGCTTTGCCGTTTAGTTAG<br>ATCTTACAAGAATTTATGAGTTAG<br>GAACGTTCTGCGTGACTTTATTTT<br>GTTCGACTACAATACGCTTAGCCA<br>GACCGAGACAACTAAAAGAAATG<br>TACGCTGAACGAAAATACTTAAG<br>GACTGAAATGACATGGTGGCTGC<br>GAAATACTTTTACGAATCTCCTTA<br>GACTCCGAGTCTGGCCCAGCCATT<br>GCGTGAACCGACATTGCGTGAAC<br>CGAGGTACCAGATGAATTCGAAG<br>ACGCTCCAATGCTCAG	*(28)*
Guide RNAs for CRSIPR/Cas9 knockout (underlined sequence is PAM)			
g*CNAG3* KO1	human	5'-<br>TCAGTGATACCAACAGGCTGTGG	[19]
g*CNGA3* KO2	human	5'-<br>GACGACCACGCAGTTCAAGCTGG	This paper
g*FAM107A* KO1	human	5'-<br>TCTTGATGAGCTGGTTCCGCCGG	[19]
g*FAM107A* KO2	human	5'-<br>GTGTCCTAGAGCACCGCCGGCGG	[19]
g*LacZ*	N/A	5'-<br>TGCGAATACGCCCACGCGATCGG	[20]

PCR primers for gRNA validation			
CNGA3_Fwd	human	5’- TGCATTTCCTGTAGTAATGG	This paper
CNGA3_Rev	human	5’- GTCAAACCACCGGATAACC	This paper
FAM107A_Fwd	human	5’- CTGGTTCAAATCACTCACCC	This paper
FAM107A_Rev	human	5’- GGTGGGGAGAAATAGATGC	This paper

Supplementary Information Table 2. Reagents and resources.

Reagent or Resource	Source	Identifier
Antibodies		
Rat anti-Ctip2/Bcl11b (1:250)	Abcam ab18465	RRID:AB_2064130
Rabbit anti-Tbr2/Eomes (1:300)	Abcam ab23345	RRID:AB_778267
Rabbit anti-Pax6 (1:350)	Abcam ab195045	RRID:AB_2750924
Rabbit anti-Foxg1 (1:200)	Abcam ab18259	RRID:AB_732415
Goat anti-Sox2 (1:200)	R&D Systems AF2018	RRID:AB_355110
Mouse anti-CNGA3 (1:50)	Invitrogen MA3-901	RRID:AB_2081689
Rabbit anti-FAM107A (1:250)	Proteintech 12176-1-AP	RRID:AB_2877832
Rabbit anti-HOPX (1:200)	Atlas Antibodies HPA030180	RRID:AB_10603770
Mouse anti-Vimentin (phospho Ser55) (1:200)	Abcam ab22651	RRID:AB_447222
Chicken anti-GFP (1:2,000)	Abcam ab13970	RRID:AB_300798
Rat anti-RFP (Clone 5f8) (1:500)	ChromoTek 5f8	RRID:AB_2336064
ZO-1 Monoclonal Antibody (ZO1-1A12), Alexa Fluor™ 647	Invitrogen MA3-39100-A647	RRID:AB_2663167
FITC Anti-Ctip2/Bcl11b antibody [25B6]	Abcam ab123449	RRID:AB_10973033
V450 Mouse anti-Sox2	BD Biosciences 561610	RRID:AB_10712763
PE Mouse anti-Sox2	BD Biosciences 562195	RRID:AB_10895118
Alexa Fluor 488 Mouse anti-Sox2	BD Biosciences 561593	RRID:AB_10894382
EOMES Monoclonal Antibody (WD1928), PE	Invitrogen 12-4877-42	RRID:AB_2572615
Donkey anti-Rat IgG, Alexa Fluor 488 conjugated	Invitrogen A-21208	RRID:AB_141709
Goat anti-Rabbit IgG, Alexa Fluor 405 conjugated	Invitrogen A-31556	RRID:AB_221605
Donkey anti-Goat IgG, Alexa Fluor 647 conjugated	Invitrogen A-21447	RRID:AB_2535864
Donkey anti-Rabbit IgG, Alexa Fluor 555 conjugated	Invitrogen A-31572	RRID:AB_162543
Donkey anti-Chicken IgG, Alexa Fluor 488 conjugated	Jackson Immuno Research 703-545-155	RRID:AB_2340375
Donkey anti-Rabbit IgG, Alexa Fluor 647 conjugated	Invitrogen A31573	RRID:AB_2536183
Donkey anti-Mouse IgG, Alexa Fluor 488 conjugated	Invitrogen A21202	RRID:AB_141607
Donkey anti-Mouse IgG, Alexa Fluor 555 conjugated	Invitrogen A31570	RRID:AB_2536180
Donkey anti-Rat IgG, Alexa Fluor Plus 555 conjugated	Invitrogen A48270	RRID:AB_2896336
Donkey anti-Rabbit IgG, Alexa Fluor Plus 488 conjugated	Invitrogen A32790TR	RRID:AB_2866495
Bacterial and virus strains		
Subcloning Efficiency DH5α competent cells	Invitrogen	Cat. # 18265017
Biological samples		
GW13/14 human foetal brain tissue	This study	N/A
Chemicals, peptides, and recombinant proteins		
DiI	Invitrogen	Cat. #D3911
CellTracker CM-DiI Dye	Thermo Scientific	Cat. # C7000
DAPI	Roche	Cat. # 10236276001
Dimethyl Sulfoxide (DMSO)	Sigma Aldrich	Cat. # D2650-100ML
Tyrode's Salts (TS)	Sigma Aldrich	Cat. # T2145
36.5% formaldehyde solution	Sigma Aldrich	Cat. # F8775
Glycine	Sigma Aldrich	Cat. # G8898
Saponin	Sigma Aldrich	Cat. # 47036
RNasin Ribonuclease Inhibitor	Promega	Cat. # N2165
Bovine Serum Albumin (BSA)	Sigma Aldrich	Cat. # A2153
EDTA disodium salt dihydrate	Carl Roth	Cat. # 8043.1

Random Hexamers	Thermo Scientific	Cat. # N8080127
10 mM dNTPs	Thermo Scientific	Cat. # R0191
Triton X-100	Sigma Aldrich	Cat. # T9284
RNaseOUT Recombinant Ribonuclease Inhibitor	Invitrogen	Cat. # 10777019
SuperScript III Reverse Transcriptase, 5x First Strand Buffer, 100 mM DTT	Thermo Scientific	Cat. # 18080093
LightCycler 480 SYBR Green I Master	Roche	Cat. # 06 991 076 702
NP40	Sigma/Roche	Cat. # 11332473001
Tween-20	Sigma/Roche	Cat. # 11332465001
Digitonin	Promega	Cat. # G944A
Nextera DNA Flex Library Prep Kit	Illumina	Cat. # FC-121-1030
Proteinase K	Thermo Scientific	Cat. # EO0491
NEBNext High- Fidelity 2x PCR Master Mix	New England Biolabs	Cat. # M0541S
SYBR Green I	Thermo Scientific	Cat. # S7563
AMPure XP beads	Beckman Coulter	Cat. # A63880
NovaSeq 6000 S4 Reagent Kit v1.5 (200 cycles)	Illumina	Cat. # 20028313
SuperScript II Reverse Transcriptase, 5x First Strand Buffer, 100 mM DTT	Thermo Scientific	Cat. # 18064071
KAPA HiFi HotStart ReadyMix	Roche	Cat. # 9420398001
Illumina DNA Prep, (M) Tagmentation	Illumina	Cat. # 20060059
RNase Inhibitor, Murine	New England Biolabs	Cat. # M0314L
Sera-Mag SpeedBeads	Cytvia	Cat. # GE24152105050250
Heparin sodium salt from porcine intestinal mucosa	Sigma Aldrich	Cat. # H4784
Trypsin-EDTA	Gibco	Cat. # 25300054
Collagenase Type IV	Gibco	Cat. # 17104019
DMEM/F12	Gibco	Cat. # 31330
Neurobasal	Gibco	Cat. # 21103049
OptiMEM	Gibco	Cat. # 31985070
Penicillin/ Streptomycin	Gibco	Cat. # 15140122
Fetal Bovine Serum (FBS)	Sigma	Cat. #F7524
MEM Non-Essential Amino Acids Solution (100X)	Gibco	Cat. # 11140050
GlutaMAX Supplement	Gibco	Cat. # 35050038
B-27 Supplement (50X), serum-free with VitA	Gibco	Cat. # 17504044
B-27 Supplement (50X), minus VitA	Gibco	Cat. # 12587010
N-2 Supplement (100X)	Gibco	Cat. # 17502048
β-Mercaptoethanol	Gibco	Cat. # 21985023
TrypLE Express Enzyme (1x), no phenol red	Gibco	Cat. # 12604021
ReLeSR	Stem Cell Technologies	Cat. # 05873
Rock inhibitor, Y-27632 (2HCl)	Stem Cell Technologies	Cat. # 72308
mTeSR1 Complete Kit	Stem Cell Technologies	Cat. # 85850
StemFlex Medium	Gibco	Cat. # A3349401
Matrigel hESC-Qualified Matrix, LDEV-free	Corning	Cat. # 354277
STEMdiff Cerebral Organoid Kit	Stem Cell Technologies	Cat. # 8570
STEMdiff Cerebral Organoid Maturation Kit	Stem Cell Technologies	Cat. # 8571
Amphotericin B (Fungizone)	Gibco	Cat. # 11520496
KnockOut Serum Replacement	Gibco	Cat. # 10828010
Insulin Solution, Human recombinant	Sigma Aldrich	Cat. # I9278
A83-01	Stem Cell Technologies	Cat. # 72022
Dorsomorphine	Stem Cell Technologies	Cat. # 72102
CHIR-99021	Stem Cell Technologies	Cat. # 72052
SB-431542	Stem Cell Technologies	Cat. # 72232

Recombinant Human/Murine/Rat BDNF	Gibco	Cat. # 450-02
Recombinant Human GDNF	Gibco	Cat. # 450-10
Ascorbic acid	Sigma Aldrich	Cat. # 1043003
Dibutyryl-cAMP	Stem Cell Technologies	Cat. # 73882
UltraPure low melting point agarose	Invitrogen	Cat. # 16520100
Matrigel Growth Factor Reduced (GFR) Basement Membrane Matrix, LDEV-free	Corning	Cat. # 354230
Trypan Blue Solution, 0.4%	Gibco	Cat. # 15250061
SPRIselect Beads	Beckman Coulter	Cat. # B23317
KpnI-HF	New England Biolabs	Cat. # R3142S
BmtI-HF	New England Biolabs	Cat. # R3658S
T4 DNA Ligase	New England Biolabs	Cat. # M0202
Quick CIP Phosphatase	New England Biolabs	Cat. # M0525
Horse Serum, heat inactivated	Gibco	Cat. # 26050088
Mowiol 4-88	Sigma Aldrich	Cat. # 81381
Critical commercial assays		
MACS Neural Tissue Dissociation Kit (P)	Miltenyi Biotec	Cat. # 130-092-628
Quick-RNA FFPE MiniPrep Kit	Zymo Research	Cat. # R1008
DNA Clean and Concentrator-5 Kit	Zymo Research	Cat. # D4014
Lipofectamine 2000	Invitrogen	Cat. # 11668027
QIAprep Spin Miniprep Kit	Qiagen	Cat. # 27106
Dual-Luciferase Reporter Assay System	Promega	Cat. # E1910
P3 Primary Cell 4DNucleofector™ X Kit S	Lonza	Cat. # V4XP-3032
QIAquick Gel Extraction Kit	Qiagen	Cat. # 28706
Experimental models: Cell lines		
Human: UCSFi001-A / WTC11	[21]	RRID:CVCL_Y803
Human: CRTDi004-A	[22]	RRID:CVCL_YR23
Human: HPSI0114i-kolf_2-C1	Wellcome Trust Sanger Institute, HipSci	RRID:CVCL_9S58
Gorilla: GC1 iPSC	[23]	N/A
Chimpanzee: SandraA iPSC	[24]	N/A
Mouse: Neuro-2A		N/A
Oligonucleotides		
Oligonucleotides, see Table S1	This paper	N/A
Software and algorithms		
Fiji/ImageJ	Fiji/ImageJ	https://imagej.nih.gov/ij/
ZEN black (11.0.1.190) or ZEN blue (v2012)	Zeiss	N/A
Prism (8.4.3)	GraphPad software	N/A
Geneious Prime (2019.2.1)	Biomatters Ltd.	N/A
Adobe Illustrator (27.2)	Adobe	N/A
Affinity Photo + Designer (1.10.5.1342)	Serif Ltd.	N/A
FACSDiva (8.0.2 and 9.0.1)	BD Biosciences	N/A
FastQC (v0.11.6)	Babraham Bioinformatics	https://www.bioinformatics.babraham.ac.uk/projects/fastqc/
STAR	[25]	
ENCODE ATAC-seq pipeline	[26]	
kallisto	[27]	
HALPER	[28]	
DESeq2	[29]	
MonaLisa	[30]	

Other		
Phylogenetic pictures	N/A	https://www.phylopic.org/
Light-Cycler 480 Multiwell Plate 96, white	Roche	Cat. # 04729692001
DNA LoBind microcentrifuge tubes, 1.5 mL	Eppendorf	Cat. # 0030108051
Falcon 5 mL Round Bottom Polystyrene Test Tube, with Snap Cap, Sterile	Corning	Cat. # 352058
Falcon 5 mL Round Bottom Polystyrene Test Tube, with Cell Strainer Snap Cap	Corning	Cat. # 352235
96-well, white wall, clear bottom plates	Greiner	Cat. # 655095
Nunc Cell-Culture Treated Multidishes, 6-well	Thermo Scientific	Cat. # 140675
96-well Clear Round Bottom Ultra-Low Attachment Microplate, with lid, sterile	Corning	Cat. # 7007
Nunc IVF Dishes, 54 mm diameter	Thermo Scientific	Cat. # 150270
6-well Clear Flat Bottom Ultra-Low Attachment Well Plates, Sterile	Corning	Cat. # 3471
pGL4.23[luc2/minP]	Promega	GenBank: DQ904455.1
pRL-CMV	Promega	GenBank: AF025843.2
pCAGS-EGFP	[31]	Addgene # 11160
pCAG-*FAM107A*	This paper	N/A
pMPRA1 plasmid backbone	Melnikov A, Zhang X, Rogov P, Wang L, Mikkelsen TS (2014) Massively parallel reporter assays in cultured mammalian cells. J Vis Exp 90:51719.	Addgene # 49349
pCAG-dCas9-KRAB-MeCP2-mCherry	(*67*)	
pCRISPRia-v2 (CRISPRi/a V2 library parental plasmid)	Horlbeck et al Elife. 2016 Sep 23;5. pii: e19760. doi: 10.7554/eLife.19760.	Addgene # 84832

Supplementary References

1 Gomez-Robles, A., Nicolaou, C., Smaers, J. B. & Sherwood, C. C. The evolution of human altriciality and brain development in comparative context. *Nat Ecol Evol* **8**, 133-146 (2024). https://doi.org/10.1038/s41559-023-02253-z

2 Lindhout, F. W., Krienen, F. M., Pollard, K. S. & Lancaster, M. A. A molecular and cellular perspective on human brain evolution and tempo. *Nature* **630**, 596-608 (2024). https://doi.org/10.1038/s41586-024-07521-x

3 Benito-Kwiecinski, S. *et al.* An early cell shape transition drives evolutionary expansion of the human forebrain. *Cell* **184**, 2084-2102.e2019 (2021). https://doi.org/10.1016/j.cell.2021.02.050

4 Kanton, S. *et al.* Organoid single-cell genomic atlas uncovers human-specific features of brain development. *Nature* **574**, 418-422 (2019). https://doi.org/10.1038/s41586-019-1654-9

5 Charvet, C. J. Cutting across structural and transcriptomic scales translates time across the lifespan in humans and chimpanzees. *Proc Biol Sci* **288**, 20202987 (2021). https://doi.org/10.1098/rspb.2020.2987

6 Charvet, C. J., Ofori, K., Falcone, C. & Rigby Dames, B. A. Transcription, structure, and organoids translate time across the lifespan of humans and great apes. *PNAS Nexus* **2**, pgad230 (2023). https://doi.org/10.1093/pnasnexus/pgad230

7 Pollen, A. A. *et al.* Establishing Cerebral Organoids as Models of Human-Specific Brain Evolution. *Cell* **176**, 743-756 e717 (2019). https://doi.org/10.1016/j.cell.2019.01.017

8 Sloan, S. A. *et al.* Human Astrocyte Maturation Captured in 3D Cerebral Cortical Spheroids Derived from Pluripotent Stem Cells. *Neuron* **95**, 779-790 e776 (2017). https://doi.org/10.1016/j.neuron.2017.07.035

9 Orvis, J. *et al.* gEAR: Gene Expression Analysis Resource portal for community-driven, multi-omic data exploration. *Nat Methods* **18**, 843-844 (2021). https://doi.org/10.1038/s41592-021-01200-9

10 He, Z. *et al.* An integrated transcriptomic cell atlas of human neural organoids. *Nature* **635**, 690-698 (2024). https://doi.org/10.1038/s41586-024-08172-8

11 Xiao, Z., Dai, Z. & Locasale, J. W. Metabolic landscape of the tumor microenvironment at single cell resolution. *Nat Commun* **10**, 3763 (2019). https://doi.org/10.1038/s41467-019-11738-0

12 Verkerke, M. *et al.* Transcriptomic and morphological maturation of human astrocytes in cerebral organoids. *Glia* **72**, 362-374 (2024). https://doi.org/10.1002/glia.24479

13 Salamon, I. *et al.* Celf4 controls mRNA translation underlying synaptic development in the prenatal mammalian neocortex. *Nat Commun* **14**, 6025 (2023). https://doi.org/10.1038/s41467-023-41730-8

14 Micali, N. *et al.* Molecular programs of regional specification and neural stem cell fate progression in macaque telencephalon. *Science* **382**, eadf3786 (2023). https://doi.org/10.1126/science.adf3786

15 Chen, A. *et al.* Spatiotemporal transcriptomic atlas of mouse organogenesis using DNA nanoball-patterned arrays. *Cell* **185**, 1777-1792 e1721 (2022). https://doi.org/10.1016/j.cell.2022.04.003

16 Kalebic, N. *et al.* Neocortical Expansion Due to Increased Proliferation of Basal Progenitors Is Linked to Changes in Their Morphology. *Cell Stem Cell* **24**, 535-550 e539 (2019). https://doi.org/10.1016/j.stem.2019.02.017

17 Ossola, C. *et al.* Adducins regulate morphology and fate of neural progenitors during neocortical neurogenesis. *Cell Rep* **44**, 116276 (2025). https://doi.org/10.1016/j.celrep.2025.116276

18 Florio, M. *et al.* Human-specific gene ARHGAP11B promotes basal progenitor amplification and neocortex expansion. *Science* **347**, 1465-1470 (2015). https://doi.org/10.1126/science.aaa1975

19 Goncalves, E. *et al.* Minimal genome-wide human CRISPR-Cas9 library. *Genome Biol* **22**, 40 (2021). https://doi.org/10.1186/s13059-021-02268-4

20 Kalebic, N. *et al.* CRISPR/Cas9-induced disruption of gene expression in mouse embryonic brain and single neural stem cells in vivo. *EMBO Rep* **17**, 338-348 (2016). https://doi.org/10.15252/embr.201541715

21 Kreitzer, F. R. *et al.* A robust method to derive functional neural crest cells from human pluripotent stem cells. *Am J Stem Cells* **2**, 119-131 (2013).

22 Völkner, M. *et al.* HBEGF-TNF induce a complex outer retinal pathology with photoreceptor cell extrusion in human organoids. *Nat Commun* **13**, 6183 (2022). https://doi.org/10.1038/s41467-022-33848-y

23 Wunderlich, S. *et al.* Primate iPS cells as tools for evolutionary analyses. *Stem Cell Res* **12**, 622-629 (2014). https://doi.org/10.1016/j.scr.2014.02.001

24 Mora-Bermudez, F. *et al.* Differences and similarities between human and chimpanzee neural progenitors during cerebral cortex development. *Elife* **5** (2016). https://doi.org/10.7554/eLife.18683

25 Dobin, A. *et al.* STAR: ultrafast universal RNA-seq aligner. *Bioinformatics* **29**, 15-21 (2013). https://doi.org/10.1093/bioinformatics/bts635

26 Lee, J. *et al.* kundajelab/atac_dnase_pipelines: 0.3.3. (2016). https://doi.org/10.5281/zenodo.211733

27 Bray, N. L., Pimentel, H., Melsted, P. & Pachter, L. Near-optimal probabilistic RNA-seq quantification. *Nat Biotechnol* **34**, 525-527 (2016). https://doi.org/10.1038/nbt.3519
28 Zhang, X., Kaplow, I. M., Wirthlin, M., Park, T. Y. & Pfenning, A. R. HALPER facilitates the identification of regulatory element orthologs across species. *Bioinformatics* **36**, 4339-4340 (2020). https://doi.org/10.1093/bioinformatics/btaa493
29 Love, M. I., Huber, W. & Anders, S. Moderated estimation of fold change and dispersion for RNA-seq data with DESeq2. *Genome Biol* **15**, 550 (2014). https://doi.org/10.1186/s13059-014-0550-8
30 Machlab, D. *et al.* monaLisa: an R/Bioconductor package for identifying regulatory motifs. *Bioinformatics* **38**, 2624-2625 (2022). https://doi.org/10.1093/bioinformatics/btac102
31 Matsuda, T. & Cepko, C. L. Electroporation and RNA interference in the rodent retina in vivo and in vitro. *Proc Natl Acad Sci U S A* **101**, 16-22 (2004). https://doi.org/10.1073/pnas.2235688100
