# Supplementary material for: Human-specific morphoregulatory signatures in basal radial glia characterise neocortex evolution
Source: Nat Commun. 2026 Oct 9;17:10651. doi: 10.1038/s41467-026-78474-0 (PMC13652802; doi:10.1038/s41467-026-78474-0)
Supplement: Supplementary file 2 [file 41467_2026_78474_MOESM2_ESM.pdf]

Description of Additional Supplementary Files

File Name: Supplementary Data 1

Description: Candidate cis-regulatory elements with human-increased chromatin accessibility in bRG.

Human-increased chromatin accessibility in bRG candidate cis-regulatory elements (hbRG cCRE) is identified by overlapping regions found to be significantly more accessible ($\log2FC \geq 1.5$; $p \leq 0.05$) in human bRG than in both chimpanzee and gorilla. Regions are benchmarked against peaks in human fetal bRG. Regions significantly more accessible in all cell types of each species are removed.

File Name: Supplementary Data 2

Description: Differentially expressed genes.

a, Differentially expressed genes human cerebral organoid aRG vs chimpanzee cerebral organoid aRG. $\log FC \geq 1$, $p.adjust \leq 0.05$. Genes significantly upregulated in all cell types of each species removed. Differential expression was assessed using the two-sided Wald test implemented in DESeq2. P values were adjusted for multiple comparisons using the Benjamini–Hochberg method. **b**, Differentially expressed genes human cerebral organoid bRG vs chimpanzee cerebral organoid bRG. $\log FC \geq 1$, $p.adjust \leq 0.05$. Genes significantly upregulated in all cell types of each species removed. Differential expression was assessed using the two-sided Wald test implemented in DESeq2. P values were adjusted for multiple comparisons using the Benjamini–Hochberg method. **c**, Differentially expressed genes human cerebral organoid bIP vs chimpanzee cerebral organoid bIP. $\log FC \geq 1$, $p.adjust \leq 0.05$. Genes significantly upregulated in all cell types of each species removed. Differential expression was assessed using the two-sided Wald test implemented in DESeq2. P values were adjusted

for multiple comparisons using the Benjamini–Hochberg method. **d**, Differentially expressed genes human cerebral organoid N vs chimpanzee cerebral organoid N. logFC $\geq$ 1, p.adjust $\leq$ 0.05. Genes significantly upregulated in all cell types of each species removed. Differential expression was assessed using the two-sided Wald test implemented in DESeq2. P values were adjusted for multiple comparisons using the Benjamini–Hochberg method. **e**, Genes significantly upregulated in all human cerebral organoid cell types vs chimpanzee. logFC $\geq$ 1, p.adjust $\leq$ 0.05. Differential expression was assessed using the two-sided Wald test implemented in DESeq2. P values were adjusted for multiple comparisons using the Benjamini–Hochberg method. **f**, Genes significantly upregulated in all chimpanzee cerebral organoid cell types vs human. logFC $\geq$ 1, p.adjust $\leq$ 0.05. Differential expression was assessed using the two-sided Wald test implemented in DESeq2. P values were adjusted for multiple comparisons using the Benjamini–Hochberg method. **g**, Human CeO, chimpanzee CeO, gorilla CeO, human CO DESeq2 regularized logarithm (rlog) transformed expression of hbRG v cbRG upregulated genes (see also Data S2b). **h**, Human CeO, chimpanzee CeO, gorilla CeO, human CO DESeq2 regularized logarithm (rlog) transformed expression of cbRG v hbRG upregulated genes.

File Name: Supplementary Data 3

Description: RNA-seq data.

Gene expression (TPM) for all cell types of cCeO, hCeO and hFetal tissue from 2-3 replicates.
