# Supplementary material for: Human-specific morphoregulatory signatures in basal radial glia characterise neocortex evolution
Source: Nat Commun. 2026 Oct 9;17:10651. doi: 10.1038/s41467-026-78474-0 (PMC13652802; doi:10.1038/s41467-026-78474-0)
Supplement: Supplementary file 3 [file 41467_2026_78474_MOESM6_ESM.pdf]

nature portfolio

Corresponding author(s): Mareike Albert

Last updated by author(s): Apr 17, 2026

Reporting Summary

Nature Portfolio wishes to improve the reproducibility of the work that we publish. This form provides structure for consistency and transparency in reporting. For further information on Nature Portfolio policies, see our Editorial Policies and the Editorial Policy Checklist.

Statistics

For all statistical analyses, confirm that the following items are present in the figure legend, table legend, main text, or Methods section.

n/a	Confirmed	
☐	☒	The exact sample size (*n*) for each experimental group/condition, given as a discrete number and unit of measurement
☐	☒	A statement on whether measurements were taken from distinct samples or whether the same sample was measured repeatedly
☐	☒	The statistical test(s) used AND whether they are one- or two-sided<br>*Only common tests should be described solely by name; describe more complex techniques in the Methods section.*
☐	☒	A description of all covariates tested
☐	☒	A description of any assumptions or corrections, such as tests of normality and adjustment for multiple comparisons
☐	☒	A full description of the statistical parameters including central tendency (e.g. means) or other basic estimates (e.g. regression coefficient) AND variation (e.g. standard deviation) or associated estimates of uncertainty (e.g. confidence intervals)
☐	☒	For null hypothesis testing, the test statistic (e.g. *F*, *t*, *r*) with confidence intervals, effect sizes, degrees of freedom and *P* value noted<br>*Give P values as exact values whenever suitable.*
☒	☐	For Bayesian analysis, information on the choice of priors and Markov chain Monte Carlo settings
☒	☐	For hierarchical and complex designs, identification of the appropriate level for tests and full reporting of outcomes
☐	☒	Estimates of effect sizes (e.g. Cohen's *d*, Pearson's *r*), indicating how they were calculated

Our web collection on statistics for biologists contains articles on many of the points above.

Software and code

Policy information about availability of computer code

Data collection	Pre-processed single-cell RNA-seq data, including alignment and annotation, were obtained in RDS format from Mendeley Data as provided by the authors of the original study (Kanton et al 2019). From this dataset, we used human and chimpanzee brain organoid samples collected at 2, 3, and 4 months of age.<br>ATAC-seq peak sets from organoid 'd60d120' peaks, Kanton et al. 2019 Supplementary Table 10.<br>We used the gEAR platform (Orvis et al 2021) to explore gene expression patterns of the 465 hbRG DEGs across publicly available multi-omic datasets (Micali et al. 2023, Chen et al. 2022, Salamon et al. 2023). For UMAPs, Gene expression projection values were calculated using the platform's projection tool (Principal Component Analysis, z-score-normalised gene expression) and downloaded at single-cell resolution to optimise visualisation.<br>To examine the expression of candidate genes across defined cellular subpopulations, we used the NeMO Analytics platform (gEAR framework). Publicly available scRNA-seq datasets (Kanton et al. 2019, Pollen et al. 2019, Sloan et al. 2017, Micali et al 2023) were queried using the dataset curator tool to generate single-gene expression visualisations, with cells filtered based on annotated cell types and developmental stages.
Data analysis	R (v4.4.1), DESeq2, monaLisa (v1.10.0), Motifmatchr (v1.26.0), GenomicRanges, 'enrichr' (v3.2), 'org.Hs.eg.db' (v3.19.1) and 'clusterProfiler' (v4.12.6), HALPER (Zhang et al. 2020; Kaplow et al. 2023), Seurat (v5.3.1), EnsDb.Hsapiens.v86, kallisto (v0.64.1), FastQC (v0.11.6), Tximport, ENCODE ATAC pipeline (Lee et al. 2016), Fiji/ImageJ (Schindelin et al. 2012), StarDist (Schmidt et al.), Prism (Graphpad Software), BD FACSDiva Software (v8.0.2 or v9.0.1)

For manuscripts utilizing custom algorithms or software that are central to the research but not yet described in published literature, software must be made available to editors and reviewers. We strongly encourage code deposition in a community repository (e.g. GitHub). See the Nature Portfolio guidelines for submitting code & software for further information.

Data

Policy information about availability of data

All manuscripts must include a data availability statement. This statement should provide the following information, where applicable:

- Accession codes, unique identifiers, or web links for publicly available datasets
- A description of any restrictions on data availability
- For clinical datasets or third party data, please ensure that the statement adheres to our policy

Great ape raw and processed RNA-seq and ATAC-seq data generated in this study have been deposited in the Gene Expression Omnibus (GEO) database under accession number GSE331438 (https://www.ncbi.nlm.nih.gov/geo/query/acc.cgi?acc=GSE331438). De-identified human and great ape ATAC-seq data have been deposited at Zenodo (DOI 10.5281/zenodo.14745099). The raw human data are protected and are not available due to data privacy laws and ethical permissions. Processed ATAC-seq and RNA-seq data are available in Supplementary Data Tables 1–3. Source Data are provided with this paper.

Research involving human participants, their data, or biological material

Policy information about studies with human participants or human data. See also policy information about sex, gender (identity/presentation), and sexual orientation and race, ethnicity and racism.

Reporting on sex and gender	Due to the protection of data privacy, the sex of the human foetuses supplying the tissue cannot be disclosed; however, sex is unlikely to impact this study's outcomes significantly.
Reporting on race, ethnicity, or other socially relevant groupings	n/a
Population characteristics	n/a
Recruitment	n/a
Ethics oversight	Human foetal brain tissue was obtained from two sources. Firstly, the Department of Gynecology and Obstetrics, University Clinic Carl Gustav Carus of the Technische Universität Dresden, following elective pregnancy termination and informed written maternal consent and with approval of the local University Hospital Ethical Review Committee (IRB00001473; IORG0001076; ethical approval number EK 355092018). Second, human foetal material was provided by the Joint MRC/ Wellcome Trust (grant# MR/X008304/1 and 226202/Z/22/Z) Human Developmental Biology Resource (http://hdbr.org).

Note that full information on the approval of the study protocol must also be provided in the manuscript.

Field-specific reporting

Please select the one below that is the best fit for your research. If you are not sure, read the appropriate sections before making your selection.

[x] Life sciences [ ] Behavioural & social sciences [ ] Ecological, evolutionary & environmental sciences

For a reference copy of the document with all sections, see nature.com/documents/nr-reporting-summary-flat.pdf

Life sciences study design

All studies must disclose on these points even when the disclosure is negative.

Sample size	For sequencing experiments, sample size was determined by cell-line and clinical sample availability. For all other experiments we adhered to field-specific standards of a minimum of 3 biological replicates per condition.
Data exclusions	In line with human-origin tissue data protection, all genes annotated to the X and Y chromosomes based on Ensembl BioMart GRCh38.p14 annotations were excluded from analysis.
Replication	Attempts of replication were successful. Findings from RNA-seq and ATAC-seq analysis were supported by analysis of published datasets from mouse, macaque, chimpanzee and human.
Randomization	Organoids were randomly assigned from each differentiation batch for experiments.
Blinding	Investigators were blinded to group allocation during both data collection and analysis for all KO, OE and image quantification experiments.

Reporting for specific materials, systems and methods

We require information from authors about some types of materials, experimental systems and methods used in many studies. Here, indicate whether each material, system or method listed is relevant to your study. If you are not sure if a list item applies to your research, read the appropriate section before selecting a response.

Materials & experimental systems

n/a	Involved in the study
☐	☒ Antibodies
☐	☒ Eukaryotic cell lines
☒	☐ Palaeontology and archaeology
☒	☐ Animals and other organisms
☒	☐ Clinical data
☒	☐ Dual use research of concern
☒	☐ Plants

Methods

n/a	Involved in the study
☒	☐ ChIP-seq
☐	☒ Flow cytometry
☒	☐ MRI-based neuroimaging

Antibodies

Antibodies used	The details of all antibodies are listed in Supplementary Information Table 2.
Validation	For all antibodies, RRDI identifiers are listed in Supplementary Information Table 2 that link to company websites and previous publications.

Eukaryotic cell lines

Policy information about cell lines and Sex and Gender in Research

Cell line source(s)	WTC11 (XY; UCSFi001-A; Gladstone Institutes, distributed by Coriell Institutes), HPSI0114i-kolf_2-C1 (XY; Wellcome Trust Sanger Institute, HipSci), CRTDi004-A (XY), SandraA (XX), GC1 (XX).
Authentication	The cell lines were not authenticated.
Mycoplasma contamination	All cell lines tested negative for mycoplasma contamination
Commonly misidentified lines (See ICLAC register)	None of the cell lines are listed as commonly misidentified.

Plants

Seed stocks	n/a
Novel plant genotypes	n/a
Authentication	n/a

Flow Cytometry

Plots

Confirm that:

☒ The axis labels state the marker and fluorochrome used (e.g. CD4-FITC).

☒ The axis scales are clearly visible. Include numbers along axes only for bottom left plot of group (a 'group' is an analysis of identical markers).

☒ All plots are contour plots with outliers or pseudocolor plots.

☐ A numerical value for number of cells or percentage (with statistics) is provided.

Methodology

Sample preparation	Cerebral organoids or human foetal tissue was DiI labelled or natively incubated for 4 hours and up to overnight. Tissue was subsequently dissociated into single cells, fixed, permeabilized and stained in suspension as detailed in the manuscript.
Instrument	FACSAria II or FACSAria III
Software	BD FACSDiva Software (v8.0.2 or v9.0.1)

Cell population abundance

n/a

Gating strategy

The main cell populations were defined using side scatter (SSC) area and forward scatter (FSC) area. Cell debris and aggregates are excluded using FSC height against FSC-A and SSC width against SSC-H. For each experiment, cells of each population were sorted into separate tubes from the same cell suspension and using identical gating strategies: 50,000 cells for ATAC-seq, 10,000 or 5,000 cells for RNA-seq, and 5,000 cells for analysis by RT-qPCR. Deep-layer neurons (DL-N) and basal intermediate progenitor cells (bIPs) were isolated from cell suspensions derived from the unlabelled organoid pool. DL-N were defined as SOX2–/BCL11B+/ZO1–/EOMES– and gated in this hierarchy. bIPs were defined as not restricted on BCL11B expression (BCL11B±) and gated for EOMES+/SOX2–/ZO1– in this hierarchy. Apical and basal radial glia were isolated from cell suspensions derived from the DiI-labelled organoid pool. aRG were defined as SOX2+/ZO-1+/DiI+, while bRG were defined as SOX2+/ZO-1–/DiI+ and gated in this hierarchy. BCL11B staining was used as an internal biological control, but not gated, as radial glia target cells were always BCL11B–. SOX2 and PAX6 antibodies were used interchangeably.

☒ Tick this box to confirm that a figure exemplifying the gating strategy is provided in the Supplementary Information.
