# Supplementary material for: Human-specific morphoregulatory signatures in basal radial glia characterise neocortex evolution
Source: Nat Commun. 2026 Oct 9;17:10651. doi: 10.1038/s41467-026-78474-0 (PMC13652802; doi:10.1038/s41467-026-78474-0)
Supplement: Supplementary file 4 [file 41467_2026_78474_MOESM7_ESM.pdf]

nature portfolio

Peer Review File

Human-specific morphoregulatory signatures in basal radial glia characterise neocortex evolution

Corresponding Author: Professor Mareike Albert

This file contains all reviewer reports in order by version, followed by all author rebuttals in order by version.

Version 0:

Reviewer comments:

Reviewer #1

(Remarks to the Author)
In the article "Human -specific morphoregulatory signatures in basal radial glia characterize neocortex evolution", Schutz et al. profiled chromatin accessibility and gene expression analyses to identify cis-regulatory elements that have a potential gain in activity in humans when compared to chimpanzees and gorillas. They identified and functionally evaluated two genes—FAM107A and CNGA3—that increase the overall morphological complexity of human basal radial glia. They then discuss how these changes might underlie the observed cortical expansion in the human lineage. Although the topic is interesting, the relationship between morphological complexity of basal radial glia and cortical expansion is not novel, as previous studies have already claimed that and have functionally evaluated the role of some genes involved in this relationship between morphology and cell division potential. Nevertheless, the two genes the authors have identified likely contribute to the increased morphological complexity of huma basal radial glia. Furthermore, the idea of basal radial glia co-opting gene signatures associated with morphology of neurons is interesting. However, there are several problems, both technical and on study design, that hinder my overall enthusiasm for this study.

1) Only one iPSC line was used per species, which substantially limits the value of all the data obtained. There are several iPSC lines available for each of the species and the authors should have used at least 3 lines per species. Otherwise, it is impossible to know if they are studying differences between species, or between cell lines.

2) All three species (human, chimpanzee, and gorilla) were used for the chromatin accessibility experiments, but gorilla is suddenly dropped from the gene expression analyses. Is there any reason for this? To ensure that the differences in gene expression between human and chimpanzee are human-specific, the authors would need to have the expression of those genes in gorilla. Otherwise, it is impossible to know whether any upregulated gene in human is truly upregulated or is instead downregulated in chimpanzee. I understand that the chromatin accessibility data helps in teasing out those two hypotheses, but it would be more conclusive to have the gene expression data.

3) In the gene expression analysis, all reads were mapped against the current human genome annotation. This creates a bias for better mappability of human samples. There are several available methods to create an orthologous annotation for these species to avoid these biases, which will lead to several false positive difference in gene expression. This is especially important as there is virtually no validation of these results. I was also surprised by the PC plot in Figure 1f, in which PC2 clearly separates human from chimpanzee and gorilla. Since humans and chimpanzees are evolutionarily closer, wouldn't the authors expect they will be closer in chromatin accessibility than any of them to gorilla? Is this due to differences in the quality of genome annotation between these species?

4) The focus on genes that are potentially associated with regulating the morphology of basal radial glia seems forced, especially in the chromatin accessibility data. From all the terms, the authors select one—"Regulation of neuron projection development"—and immediately jump to the conclusion the "Human bRG may thus employ both ancestral neuronal CREs as well as increased activity of ancestral radial glia CREs to express genes classically associated with controlling morphoregulatory processes in neurons." This is a big jump, especially when there are so many other CREs and functional terms that are not related at all with this. Although the functional enrichment analysis of gene expression seems to better point to cell morphology terms (though there are several other interesting processes), this data lacks the analysis of gorilla as mentioned above. More importantly, the authors mention that they calculated a "morphoregulatory pathway activity score" but there is nothing detailed in the methods section. It would be important to first validate and benchmark this new score

against independent datasets to show that is captures what is intended.

5) The authors highlight two genes—FAM107A and CNGA3—and show that when these genes are knocked out, there is a decrease in morphological complexity of basal radial glia. Since this section of the study is novel, I expected to see whether these organoids were smaller, thus corroborating their hypothesis. Similarly, if they overexpress these genes, will the basal radial glial cells have increased morphology and the organoids be larger? The authors should also discuss what are their thoughts about the cellular mechanism(s) that are affected by the expression of these genes.

Minor concerns:
1) The authors should use the current gene names throughout the study. For example, BCL11B instead of CTIP2, and EOMES instead of TBR2.
2) Chimpanzee should be used instead of chimp.
3) Some figure panels are wrongly cited throughout the text.
4) In several figures and panels, all species should be shown. For example, if Figure 1b, representative human and chimpanzee organoids should be shown as well. In Extended Data Fig. 1, the QC of gorilla organoids should also be shown.

Reviewer #2

(Remarks to the Author)
The authors performed bulk ATAC and RNA sequencing of neurons and neural progenitor cells [apical radial glia (aRG), basal radial glia (bRG), and basal intermediate progenitors (bIPs)] separately sorted from gorilla, chimpanzee and human cerebral organoids, in addition to GW13/14 human fetal tissue. Their goal was to identify cis-regulatory elements (CREs) with potential human-specific activity gains.
Through this comparative analysis, the authors identified 599 human candidate CREs with increased chromatin accessibility in human bRG cells (hbRG cCREs) that are in a physical contact with 589 unique targets potentially involved in neuronal morphoregulatory processes. These CREs were grouped into three major clusters based on shared accessibility with other neural cell types. Using the transcriptional profile of human fetal bRG as a reference, the authors further identified 939 differentially expressed genes between chimpanzee and human bRG, showing that genes upregulated in human bRG were predominantly enriched in pathways related to the morphoregulatory dynamics of cell projections.
Although many hbRG cCRE target genes with high expression in bRG are conserved in chimpanzees, several were expressed more strongly in chimpanzee neurons than in progenitors. This pattern suggests that human bRG may have gained activity in morphoregulatory CREs, potentially co-opting neuronal gene-regulatory programs to expand and diversify their morphology. Consistent with this idea, the authors highlighted two candidates (FAM107A and GNCA3) and tested their function by CRISPR/Cas9-mediated knockout in human cortical organoids. Both genes proved functionally required for the morphological complexity of human bRG.

Overall, a major strength of the study lies in a unique dataset and new biological insights with relevance for human evolutionary and developmental neuroscience. By uncovering novel regulators of bRG morphology, it sheds light on early, cell type–specific molecular changes that may underlie the evolutionary expansion of the human cortex. I recommend that the authors be invited to address the concerns outlined below through additional experiments and revisions to the manuscript.

Major points:
1. It appears that gorilla organoids were generated using a different protocol than the one used for chimpanzee and human organoids. Biological and technical differences between organoids and across species make it very difficult to interpret combined datasets without introducing bias. That is why it is essential to generate all organoids using the standardized and optimized protocol. Only then can any observed differences be confidently interpreted as “species-specific”, rather than the result of different experimental conditions. Another important issue is the limited number of cell lines used, specifically, only one line per species for chimpanzee and gorilla. To ensure robust and reliable findings, it is important to use at least two iPSC lines per species and/or the authors should provide independent evidence using previously published human and non-human primate organoid datasets.

2. The description of the cell sorting method lacks clarity. It seems that some cell types (DL-N and bIPs) were isolated from one set of organoids (referred to as "unlabeled tissue" on line 616), while others (aRG and bRG) were obtained from a separate set of 5-10 organoids ("labeled tissue," as noted on line 614). However, this distinction is not explicitly stated in the Methods section “Fluorescence activated cell sorting”. As currently written, it could also be interpreted that all four cell types were derived from the same set of organoids and sorted into three tubes each (3 tubes × 4 cell types = 12 tubes). This clarification is important, as it directly affects the interpretability and comparability of downstream analyses. If the first scenario is correct, it raises the question of whether DL-N and bIPs can be reliably compared to aRG and bRG, given that they originate from different organoids. Ideally, all sorted cells should be derived from the same set of organoids and pooled into a homogeneous suspension before being split into two aliquots (one for bulk ATAC-seq and the other for bulk RNA-seq) to ensure consistency and facilitate meaningful comparisons across assays.

3. The authors selected an 8-week (or 54 days) time point for in vitro organoid analysis and compared this stage specifically to human prenatal development at GW13/14. However, the rationale for choosing this specific time point remains unclear and the manuscript does not adequately account for interspecies differences in developmental timing. Given that neocortical development in humans is more protracted than in great apes, it cannot be assumed that organoids from chimpanzees,

gorillas, and humans cultured for the same duration are developmentally comparable. The authors should carefully determine which in vitro developmental stage in each great ape species best corresponds to human cortical development in vivo, and compare the developmental maturation of organoids across species. They should clarify the rationale behind their time point selection and provide supporting evidence, such as transcriptomic age estimation or stage-specific marker expression, to justify developmental comparisons across species. Additionally, while the use of iPSC-derived organoids from multiple primates is a strength, the Discussion should more systematically address the limitations of organoids as developmental models, including key differences from in vivo development and how interspecies differences in developmental timing may affect interpretation.

4. The rationale for selecting FAM107A and CNGA3 genes from the 599 candidate targets is unclear. The authors should explicitly state the filtering criteria (e.g., fold change threshold, evolutionary specificity, prior functional relevance) in the Methods or Results section. Since the data presented in Fig. 4i–4n and Extended Fig. 6 are not fully convincing, it would be beneficial to validate whether the FAM107A and CNGA3 knockouts are functionally effective at the RNA and/or protein level, using RT-PCR, RNA-scope, or immunocytochemistry, particularly since the CNGA3 antibody appears to be working in Fig. 4j. Moreover, morphological analyses intended to support strong conclusions should be based on 3D reconstructions (quantitative morphometric analyses, such as total neurite length, number and order of branches, Sholl analysis, basal process thickness/terminal counts); without them, the interpretations remain largely speculative. It is also important that the control and both knockout organoids, along with zoomed-in images of GFP^{+} cells, be presented side by side for proper comparison.

Minor points:
1. The authors only present a brightfield image of a whole gorilla cerebral organoid at week 8 (Fig. 1b). From the figure, it appears that the authors are working with organoids cultured as whole spheres (unsliced) rather than sliced organoids. Please clarify this point. In addition, could the authors provide corresponding images of chimpanzee and human organoids for comparison?
2. Please provide FACS plots for organoids from all three species, not just the GW13/14 human fetal cortex sample. This is important for comparative analysis and to assess consistency across models.
3. The authors should maintain consistency by using official gene symbols rather than aliases, for example, using EOMES instead of TBR2, or BCL11b instead of CTIP2.
4. Staining with only the dye DiI and the markers EOMES and SOX2 do not provide sufficiently convincing evidence for the presence of basal radial glia (bRG) in the cerebral organoids. To support this claim, additional immunostaining is needed using bRG-specific markers (such as HOPX), as well as proliferation markers (such as pVIM), given that bRG divide within the outer subventricular zone, a characteristic feature of bRG in vivo. This analysis should be performed across all three organoid types from each species. In addition, the authors should provide channel-split images in Fig. 4i–4n to confirm that these bRG cells are indeed EOMES- but SOX2+, as claimed.
5. Please clarify whether the RT-qPCR used to confirm the identity of the sorted cell populations was performed on RNA extracted from the same sorted cells that were used for ATAC-seq or RNA-seq. If not, please explain the rationale for using a separate sorted tube/ sample.
6. As stated in line 126, the authors "leveraged cell type-specific Hi-C data of cortical organoids to link our candidate regions to their target genes." While this is a valid approach, in the context of identifying human-specific regulatory elements, it would be more appropriate to incorporate or at least consider publicly available Hi-C datasets from human fetal brains and in vitro neural progenitor cells (NPCs) of both human and chimpanzee origin (e.g., Keough et al., 2023; Pal et al., 2025; Won et al., 2019). Without direct comparative analyses using both human and chimpanzee Hi-C data, identification of hbRG cCRE activity should be made with caution. The current approach does not fully exclude the possibility that similar regulatory interactions may also be present in non-human primates.
7. Since the study primarily relies on organoid/fetal brain Hi-C data to pair bRG-specific cCREs with target genes, the authors should strengthen the evidence chain by integrating “accessibility–expression” correlation analyses across samples and cell types (bulk, single-cell, or sorted populations). This would reduce false positives from purely proximity-based assignments and improve confidence in distal enhancer–target links.
8. What was the rationale for excluding gorilla organoids from the RNA-seq analyses in Fig. 3? It is unclear whether some of the 939 identified DEGs are also expressed in bRG sorted from gorilla CeOs. The authors may have overstated their conclusion in line 148 by referring to a ”human bRG-specific phenotype”. It would also be beneficial to include gorilla datasets alongside chimpanzee data in all other analyses. In addition to the previous comment, a log2FC > 1 threshold is relatively low. Please consider using a more stringent criteria, such as log2FC > 1.5 or higher.
9. The study focuses on human-increased bRG cCRE targets, but whether FAM107A and CNGA3 have conserved or divergent functions in non-human primates has not been tested. Parallel perturbations in chimpanzees or gorilla organoids would strengthen evolutionary specificity claims.
10. Currently, only reporter assays are performed. To establish causality (“enhancer variant → target gene expression → morphological phenotype”), the authors should consider CRISPRi/CRISPRa on CREs. Parallel perturbations in aRG, bIP, and different developmental stages would demonstrate effect specificity to bRG and critical time windows. It is also important to confirm that perturbations do not alter bRG identity markers (SOX2, TBR2, ZO1), to rule out fate-switch artifacts.

Reviewer #3

(Remarks to the Author)
It is already known that bRG plays a significant role in the evolutionary expansion of brain volume and the complex folding of the cerebral cortex due to its explosive neuron-producing capacity. The attempt to clarify species-specific differences between humans and great apes, particularly in bRG, is a meaningful endeavor. Given the difficulty of analyzing great ape fetuses, the use of organoids with the selected species combination is somewhat justifiable. The study found that a

characteristic feature of the bRG expression profile in humans is the high expression level of a gene set involved in cell morphology regulation. Furthermore, knocking out two representative genes from this set in human organoids simplified the cell morphology, suggesting that human bRG has a more complex cellular morphology than that of other species.

While this manuscript has several minor points for improvement, its core argument on human-specificity of bRG is well-understood, and the work holds some value within the research community. However, a significant weakness is that the paper's evaluation of human-specific bRG features is limited to cell morphology. While a previous study Kalebic et al Cell Stem Cell 2019 has suggested a link between cell morphology and the stemness and neurogenic capacity of bRG, this research should have directly demonstrated that the observed morphological complexity leads to increased proliferation and neuron production. It's also disappointing that the majority of the gene expression comparative analysis relies on inter-species comparisons of organoid samples, even though the authors did acquire and analyze human fetal data. They should have at least confirmed the human-specific gene expression they found by comparing human and non-human primate fetal samples, as data for primates like macaques are publicly available.

Overall, a major strength of this study is its clear and simple structure. However, the depth of the individual analyses is lacking, and the fundamental finding—the human-specific regulation of bRG cell morphology—has limited importance in a broader context. For these reasons, the manuscript is not a good fit for a journal with a wide readership, such as Nature Communications. The following are more specific peer review comments.

Major concerns;
1) (Fig1d) Is the DiI labeling method, where it's placed on the organoid surface, even valid in the organoido lacking meninges and other tissues/cell types contained in the in vivo fetal brain?
2) (Fig3c) Instead of just bulk RNA-seq, scRNA-seq data should also be used to examine the expression dynamics of these genes in more detail. Specifically, the authors should describe the cell type specificity, expression dynamics along the cell differentiation axis, and detailed expression patterns by distinguishing between different cell types and developmental stages. The datasets of some previous studies (https://pubmed.ncbi.nlm.nih.gov/37824652/ etc) will provide additional value on the authors' study.
3) (Fig3f) The concept of a "morphoregulatory pathway score" is questionable. More detailed explanation of the gene set is necessary. What GO terms are selected to define them?
4) (Fig3) If a gene expression network analysis were conducted, could common upstream transcription factors for FAM107A and CNGA3 be found?
5) (Fig4d) The reporter assay results in N2A cells do show that species differences in the CRE sequence correlate with differences in expression induction activity. But can this be said for bRG as well? Given that there's an experimental system for overexpression in human and chimpanzee organoids, shouldn't this experiment be conducted in organoids, with a stronger focus on bRG expression?
6) (Fig4j-k) Immunohistochemical data for FAM107A is required as well, in addition to CNGA3.
7) (Fig4j-k) Immunohistochemical staining for CNGA3 and FAM107A should be performed in fetal brains as well. It's impossible to determine whether the images shown are truly of bRG and not aRG, by showing the exact location of the imaged cells within SVZ-like domain in the organoids.
8) (Fig4j-k) Is there a correlation between the expression levels of CNGA3 (and/or FAM107A) and cell morphology in fetal brains and brain organoids? Maybe it is useful to co-stain DiI (if it's a valid method for labeling bRG in organoids) and CNGA3 protein to demonstrate the correlation of CNGA3 level and morphological complexity.
9) (Fig4m-n) The LOF analysis of FAM107A and CNGA3 should include not only the observation of cell morphology but also assays to examine markers of cell proliferation and the number of neurons produced.
10) (Fig4m-n) In addition to LOF analysis, a GOF analysis in chimpanzee organoids should be included.
11) (Fig4m-n) The analysis should distinguish and evaluate not only bRG but also aRG cell types. Considering that these genes are also expressed at high levels in aRG, it can be speculated that they have some function. Do aRG also have morphological diversity that is regulated by these genes?
12) (Fig4m-n) The current data provides no evidence to suggest that a selective analysis of bRG was performed. Shouldn't DiI labeling be done here as well?

Version 1:

Reviewer comments:

Reviewer #1

(Remarks to the Author)
The authors have addressed all of my main concerns.

Reviewer #2

(Remarks to the Author)
The authors have addressed all of my concerns as well as those raised by the other reviewers. Their responses are thorough and the revision has been carried out with great care.

Reviewer #3

(Remarks to the Author)
General Comments:
The authors have significantly expanded the dataset in this revised manuscript, and it is evident that they have made a sincere and comprehensive effort to address all the concerns raised in the initial review. The increased volume of data has substantially strengthened the foundation of their claims.
In particular, the ATAC-seq and gene expression analyses have been greatly enriched. The original data presented here for the first time are intrinsically valuable to the research community. Furthermore, the integrated comparative analysis with other public datasets has further enhanced the significance of their original findings. Regarding the omics data, the data representation is excellent and exceptionally clear to the reader.

Specific Comments:
1. Functional Analysis of FAM107A and CNGA3 (Figure 5 and Extended Data Figure 15)
The authors have performed an impressive set of additional experiments quantifying bRG cell proliferation and neuronal differentiation in response to my previous request. While I highly commend the effort to include these analyses, I must candidly state that the results are somewhat disappointing in light of the authors' primary hypothesis.
The authors conclude that FAM107A knockout leads to simplified cell morphology and a concomitant decrease in the proportion of differentiated neurons (Lines 406-413), suggesting a link between morphological complexity and developmental consequences. However, an objective assessment of the data in Extended Data Fig. 15j and k conveys the opposite impression: the loss of FAM107A appears to have a negligible impact on the core stem cell programs, such as bRG proliferation or differentiation.
From the initial submission, my view has been that the theme of "morphological control in bRG" holds universal scientific value only if such control is shown to be essential for bRG stemness. Despite the massive addition of data, this fundamental point remains unproven. In fact, the results in Extended Data Fig. 15j and k seem to support the inverse conclusion—that bRG morphological complexity may not be as critical to the stem cell program as the authors claim.

2. Validation of FAM107A Knockout (Extended Data Figure 14)
There are also concerns regarding the technical validation of the FAM107A knockout. In the provided images, the intensity of the immunostaining signal shows almost no detectable decrease, which raises doubts as to whether the gene function was effectively abolished.
While it is helpful that the authors illustrated the exon structure and guide RNA (gRNA) targets in Extended Data Figure 14, they must provide evidence showing the actual impact at the protein level. Specifically, will the designed gRNA result in a complete null? Additionally, the authors should clarify the position of the antibody epitope relative to the gRNA target site to ensure that the observed signal is not from a truncated or partially functional protein.

Version 2:

Reviewer comments:

Reviewer #3

(Remarks to the Author)
I believe the authors have fully addressed all of my concerns, which has significantly enhanced the credibility and robustness of the manuscript. Thanks to their extensive efforts, this paper now makes a highly valuable contribution to the field.

Open Access This Peer Review File is licensed under a Creative Commons Attribution 4.0 International License, which permits use, sharing, adaptation, distribution and reproduction in any medium or format, as long as you give appropriate credit to the original author(s) and the source, provide a link to the Creative Commons license, and indicate if changes were made.
In cases where reviewers are anonymous, credit should be given to 'Anonymous Referee' and the source.
The images or other third party material in this Peer Review File are included in the article's Creative Commons license, unless indicated otherwise in a credit line to the material. If material is not included in the article's Creative Commons license and your intended use is not permitted by statutory regulation or exceeds the permitted use, you will need to obtain permission directly from the copyright holder.
To view a copy of this license, visit https://creativecommons.org/licenses/by/4.0/

REVIEWER COMMENTS

We thank all reviewers for their constructive remarks on the manuscript, which have helped us to further strengthen our data and support our conclusions. In particular, we provide RNA-seq and ATAC-seq data from two additional human iPSC lines and RNA-seq data from gorilla CeOs. In addition, we have now cross-validated our findings by comparing them with published single-cell data sets from multiple species, including mouse, macaque and human primary tissues, as well as chimpanzee and human cerebral organoids. In the revised manuscript, we present several new functional experiments in cerebral organoids. The detailed response to all comments can be found below.

Reviewer #1 (Remarks to the Author):

In the article "Human-specific morphoregulatory signatures in basal radial glia characterize neocortex evolution", Schutz et al. profiled chromatin accessibility and gene expression analyses to identify cis-regulatory elements that have a potential gain in activity in humans when compared to chimpanzees and gorillas. They identified and functionally evaluated two genes—FAM107A and CNGA3—that increase the overall morphological complexity of human basal radial glia. They then discuss how these changes might underlie the observed cortical expansion in the human lineage. Although the topic is interesting, the relationship between morphological complexity of basal radial glia and cortical expansion is not novel, as previous studies have already claimed that and have functionally evaluated the role of some genes involved in this relationship between morphology and cell division potential. Nevertheless, the two genes the authors have identified likely contribute to the increased morphological complexity of huma basal radial glia. Furthermore, the idea of basal radial glia co-opting gene signatures associated with morphology of neurons is interesting. However, there are several problems, both technical and on study design, that hinder my overall enthusiasm for this study.

We thank the reviewer for their feedback on our study and for highlighting the interest in the genomic basis of interspecies differences in neocortex development, particularly the genes regulating morphology and the concept of co-opting gene signatures. Our study extends previous work by focusing on great ape species for which our knowledge of neurodevelopment remains very limited. This reviewer raises several points on techniques and study design, which we address in the following.

1) Only one iPSC line was used per species, which substantially limits the value of all the data obtained. There are several iPSC lines available for each of the species and the authors should have used at least 3 lines per species. Otherwise, it is impossible to know if they are studying differences between species, or between cell lines.

In this study, we generated cell-type-specific transcriptomic and chromatin accessibility profiles from cerebral organoids derived from three great ape species (Fig. 1b,c), each of which reproducibly gives rise to basal radial glia (bRG), a cell type repeatedly implicated in neocortical evolution. We agree that the inclusion of multiple independent iPSC lines per species would further strengthen the generalizability of the findings. However, the availability of suitable great ape iPSC lines that robustly generate late-stage cerebral organoids remains limited. In particular, the gorilla line used here is, to our knowledge, currently the only line available in Europe that consistently produces organoids of sufficient quality for the analysis of bRG morphology. For chimpanzee, we tested an additional published iPSC line (JoC; Kanton et al., 2019), for which we managed to generate embryoid bodies and early-stage organoids consistent with previous reports (Benito-Kwiecinski et al., 2021), but did not reliably progress to later stages required for bRG analyses.

To strengthen our conclusions on inter-species comparisons, we have performed extensive analyses on several published datasets: 1) scRNA-seq and scATAC-seq from chimpanzee and human CeO (Kanton et al. 2019; Pollen et al. 2019; Sloan et al. 2017), 2) snRNA-seq from human foetal cortex across different time points (Salamon et al., 2023), 3) scRNA-seq from macaque foetal cortex (Micali et al., 2023) and 4) scRNA-seq from mouse embryonic cortex (Chen et al., 2022). In summary, the analyses indicate that human bRG-upregulated genes are embedded within conserved neuronal gene expression programs across species, but are more strongly or selectively activated in human bRG (see new Extended Data Fig. 5, 8, 10, 11 and 12).

To further corroborate the human data, our study generated RNA- and ATAC-seq datasets from foetal tissue to ensure *in vivo* relevance. In addition, to highlight the robustness of the findings, we have added new data in the revised manuscript (new Extended Data Fig. 4) from two additional human iPSC lines (CRTDi004-A; HPSI0114i-kolf_2) generated with a directed cortical organoid protocol (Qian et al. 2020). Hierarchical clustering of cell-type-specific transcriptomic data from foetal tissue, cerebral organoids (CeO), and the newly generated cortical organoids (CO) reveals that RG, bIP, and neurons cluster independently of tissue source (new Extended Data Fig. S4f). The previously identified 599 candidate hbRG cCREs are also marked by open chromatin in the new hCO data (new Extended Data Fig. 4g).

Additional supporting evidence is summarised in response to Reviewer 2, point 1. Together, these new data underscore that the results are not dependent on the specific organoid protocol or iPSC line used, but hold up across biological replicates and protocol variations.

2) All three species (human, chimpanzee, and gorilla) were used for the chromatin accessibility experiments, but gorilla is suddenly dropped from the gene expression analyses. Is there any reason for this? To ensure that the differences in gene expression between human and chimpanzee are human-specific, the authors would need to have the expression of those genes in gorilla. Otherwise, it is impossible to know whether any upregulated gene in human is truly

upregulated or is instead downregulated in chimpanzee. I understand that the chromatin accessibility data helps in teasing out those two hypotheses, but it would be more conclusive to have the gene expression data.

We agree with the reviewer and have generated RNA-seq data for the four cell types from gorilla CeO (new Extended Data Fig. 7; manuscript lines 221-236). Genes upregulated in human compared to chimpanzee bRG showed broadly lower expression in gorilla compared to human bRG, suggesting human up- rather than chimpanzee downregulation.

Moreover, we have mined scRNA-seq data from macaque foetal cortex (Micali et al., 2023). In this data set, the human bRG upregulated morphoregulatory candidate genes *BCL11A*, *ENC1*, *BAIAP2*, and *PTPRM* showed low or absent expression in bRG (Extended Data Fig. 11b, c), supporting human-specific upregulation.

3) In the gene expression analysis, all reads were mapped against the current human genome annotation. This creates a bias for better mappability of human samples. There are several available methods to create an orthologous annotation for these species to avoid these biases, which will lead to several false positive difference in gene expression. This is especially important as there is virtually no validation of these results. I was also surprised by the PC plot in Figure 1f, in which PC2 clearly separates human from chimpanzee and gorilla. Since humans and chimpanzees are evolutionarily closer, wouldn't the authors expect they will be closer in chromatin accessibility than any of them to gorilla? Is this due to differences in the quality of genome annotation between these species?

This is an important issue. First, we want to clarify that we used this approach (mapping to hg38 for all samples) only for the RNA-seq data in order to avoid issues with reference gene annotations. The ATAC-seq data were mapped to each species' own genome. Nevertheless, the reviewer is correct to caution that this approach could result in potential bias. We point to the recent preprint by Barr et al. 2025, which focuses precisely on this issue. Therefore, we decided to rigorously address mapping concerns for RNA-seq data and compared two independent strategies, inspired by the manuscript above: 1) uniform mapping to hg38 (also called single-reference in Barr et al. 2025), ranking 2nd best in the unbiased comparisons – our current approach. 2) Species-specific genome mapping using annotation transfer with the Comparative Annotation Toolkit (CAT), combined with CrossFilt reciprocal filtering. The results were highly concordant between approaches (see Extended Data Fig. 6b–d; manuscript lines 191-202).

In the revised manuscript, we validated our transcriptomic data by comparing it with multiple published scRNA-seq datasets (see response to point 1).

We also thank the reviewer for raising the point about the PCA in Fig. 1g. As ATAC-seq reads were mapped to each species' own genome, this pattern is unlikely to reflect mapping bias.

While phylogeny suggests greater similarity between humans and chimpanzees, the separation along PC2 indicates that the dominant axis of variation in this dataset distinguishes humans from the two non-human great ape species. As chimpanzee and gorilla are more similar to each other in both total brain size and cortical neuron number (Roth and Dicke, 2005; Herculano-Houzel and Kaas, 2011) than either is to human.

4) The focus on genes that are potentially associated with regulating the morphology of basal radial glia seems forced, especially in the chromatin accessibility data. From all the terms, the authors select one—"Regulation of neuron projection development"—and immediately jump to the conclusion the "Human bRG may thus employ both ancestral neuronal CREs as well as increased activity of ancestral radial glia CREs to express genes classically associated with controlling morphoregulatory processes in neurons." This is a big jump, especially when there are so many other CREs and functional terms that are not related at all with this. Although the functional enrichment analysis of gene expression seems to better point to cell morphology terms (though there are several other interesting processes), this data lacks the analysis of gorilla as mentioned above. More importantly, the authors mention that they calculated a "morphoregulatory pathway activity score" but there is nothing detailed in the methods section. It would be important to first validate and benchmark this new score against independent datasets to show that is captures what is intended.

In the revised manuscript, we have extended our discussion of the GO terms linked to chromatin accessibility data to provide a broader perspective (see manuscript lines 175–188). Additionally, we now provide RNA-seq data of gorilla CeO (see response to point 2). Moreover, we provide a more detailed description of the "morphoregulatory pathway activity score" (see manuscript lines 238–246) that we adapted from previous literature (He et al. 2024; Xiao et al., 2019). Importantly, we now validate the score in a cortical context (see new Extended Data Fig. 9), yielding the expected results (see manuscript lines 246–252).

5) The authors highlight two genes—FAM107A and CNGA3—and show that when these genes are knocked out, there is a decrease in morphological complexity of basal radial glia. Since this section of the study is novel, I expected to see whether these organoids were smaller, thus corroborating their hypothesis. Similarly, if they overexpress these genes, will the basal radial glial cells have increased morphology and the organoids be larger? The authors should also discuss what are their thoughts about the cellular mechanism(s) that are affected by the expression of these genes.

The functional analysis of the two genes was performed by electroporating individual cells in ventricle-like structures of organoids, resulting in an overall targeting of <5% of total cells in the organoid. Therefore, differences in total organoid size were not expected. However, we quantified neurons after *FAM107A* knockout in hCO and observed a significant decrease in the subventricular zone/cortical plate (new Extended Data Fig. 15j, k).

The suggestion of overexpression of the candidate genes is an interesting one, and we performed electroporation of a plasmid expressing human *FAM107A* in chimpanzee CeO (see new Extended Data Fig. 16). The phenotype was similar to the KO in human CeO, suggesting that *FAM107A* expression levels are tightly regulated by gene regulatory networks in the human developing neocortex to achieve appropriate expression levels. This is in line with previous work on the morphoregulatory protein ZNF804A, which showed that both lower and higher expression levels affected cell morphology in the same direction (Dong et al., 2021) (see manuscript lines 415–420).

In the discussion, we include a section on the cellular mechanism of the two genes (lines 445–449) and discuss how increased morphological complexity may contribute to increased neuronal output (lines 451–460).

Minor concerns:
1) The authors should use the current gene names throughout the study. For example, BCL11B instead of CTIP2, and EOMES instead of TBR2.

This was changed as requested in both the figures and text.

2) Chimpanzee should be used instead of chimp.

We now consistently use “chimpanzee”.

3) Some figure panels are wrongly cited throughout the text.

We have cross-checked all figure citations.

4) In several figures and panels, all species should be shown. For example, if Figure 1b, representative human and chimpanzee organoids should be shown as well.

We now show gorilla, chimpanzee and human data as requested (see new Fig. 1b and revised/new Fig. 4 and Extended Data Fig. 1, 2, 6 and 12).

In Extended Data Fig. 1, the QC of gorilla organoids should also be shown.

The QC is now shown for organoids from all species, including gorilla (see new Extended Data Fig. 1 and 2).

Reviewer #2 (Remarks to the Author):

The authors performed bulk ATAC and RNA sequencing of neurons and neural progenitor cells [apical radial glia (aRG), basal radial glia (bRG), and basal intermediate progenitors

(bIPs)] separately sorted from gorilla, chimpanzee and human cerebral organoids, in addition to GW13/14 human fetal tissue. Their goal was to identify cis-regulatory elements (CREs) with potential human-specific activity gains.
Through this comparative analysis, the authors identified 599 human candidate CREs with increased chromatin accessibility in human bRG cells (hbRG cCREs) that are in a physical contact with 589 unique targets potentially involved in neuronal morphoregulatory processes. These CREs were grouped into three major clusters based on shared accessibility with other neural cell types. Using the transcriptional profile of human fetal bRG as a reference, the authors further identified 939 differentially expressed genes between chimpanzee and human bRG, showing that genes upregulated in human bRG were predominantly enriched in pathways related to the morphoregulatory dynamics of cell projections.
Although many hbRG cCRE target genes with high expression in bRG are conserved in chimpanzees, several were expressed more strongly in chimpanzee neurons than in progenitors. This pattern suggests that human bRG may have gained activity in morphoregulatory CREs, potentially co-opting neuronal gene-regulatory programs to expand and diversify their morphology. Consistent with this idea, the authors highlighted two candidates (FAM107A and GNCA3) and tested their function by CRISPR/Cas9-mediated knockout in human cortical organoids. Both genes proved functionally required for the morphological complexity of human bRG.

Overall, a major strength of the study lies in a unique dataset and new biological insights with relevance for human evolutionary and developmental neuroscience. By uncovering novel regulators of bRG morphology, it sheds light on early, cell type–specific molecular changes that may underlie the evolutionary expansion of the human cortex. I recommend that the authors be invited to address the concerns outlined below through additional experiments and revisions to the manuscript.

We thank the reviewer for their encouraging feedback. This reviewer particularly highlights the unique dataset and the resulting new biological findings from our study. We appreciate the emphasis on the cell-type-specific molecular changes we uncovered, linked to the regulation of bRG morphology. In the following, we address the specific points raised by the reviewer.

Major points:
1. It appears that gorilla organoids were generated using a different protocol than the one used for chimpanzee and human organoids. Biological and technical differences between organoids and across species make it very difficult to interpret combined datasets without introducing bias. That is why it is essential to generate all organoids using the standardized and optimized protocol. Only then can any observed differences be confidently interpreted as “species-specific”, rather than the result of different experimental conditions. Another important issue is the limited number of cell lines used, specifically, only one line per species for chimpanzee and gorilla. To ensure robust and reliable findings, it is important to use at least two iPSC lines

per species and/or the authors should provide independent evidence using previously published human and non-human primate organoid datasets.

This is an important point. Cerebral organoids for gorilla, chimpanzee, and human were all generated using the same protocol (Lancaster et al., 2013; Lancaster et al., 2014). We agree that this is essential to unravel biological rather than technical differences. We have rephrased the methods section to state this more clearly and avoid confusion.

In addition, for human, we have isolated all four cell types from primary foetal tissue, cerebral organoids and now also cortical organoids (see new Extended Data Fig. 4) from different iPSC lines (3 in total). As discussed in response to reviewer 1, the 599 identified hbRG cCREs exhibit highly concordant patterns, independent of tissue source (compare Fig. 2 and Extended Data Fig. 3 and 4). This underscores the robustness of the findings.

Additionally, we have mined published datasets from human and chimpanzee cerebral organoids. For open chromatin, we observed high concordance between our dataset and that of Kanton et al. 2019 at multiple levels. First, across the full peak sets, a substantial fraction of peaks overlapped between datasets (~74% of Kanton et al. hCeO peaks overlapped our data; Jaccard similarity = 0.267; ~70% of Kanton et al. cCeO peaks overlapped our data; Jaccard similarity = 0.237). Second, focusing specifically on the 599 hbRG cCREs identified in our study, 290 overlapped peaks in the independent human dataset, whereas only 122 overlapped peaks in the chimpanzee dataset. This corresponds to an approximately 2.4-fold human-to-chimpanzee peak ratio in the independent dataset, closely matching the ~2.7-fold ratio observed in our own data. To ensure that these overlaps were not driven by the non-uniform genomic distribution of accessible chromatin, we performed both Fisher's exact tests restricted to ENCODE candidate cis-regulatory elements (cCREs) and permutation-based analyses. In both species, overlaps were highly significant (Fisher's exact test: $p < 2.2 \times 10^{-16}$; odds ratio = 73 for human and 60 for chimpanzee), and greatly exceeded random expectation based on 10,000 permutations (human: 68,839 observed vs. 16,517 expected overlaps; chimpanzee: 65,295 observed vs. 15,830 expected overlaps; permutation $p < 0.0001$). Importantly, beyond global peak overlap, the subset of hbRG cCRE candidates showed consistent accessibility patterns across cell types in both datasets, recapitulating the major clusters identified in our analysis (see new Extended Data Fig. 5).

Moreover, we performed RNA expression analyses. In our dataset, we defined a pan-radial glia (pan-RG) population by combining aRG and bRG and identified 1,462 differentially expressed genes between human and chimpanzee. Of these, 1,331 genes could be mapped to the Kanton et al. dataset, which showed broadly consistent species-specific expression patterns in radial glia populations. Importantly, this concordance extends to functionally relevant gene sets. Many morphoregulatory genes upregulated in hbRG in our dataset (Fig. 3e) also showed differential expression in independent datasets, including chimpanzee bRG from published scRNA-seq studies (n = 186 human and chimpanzee CeO bRG cells; Extended Data Fig. 8a)

and gorilla bRG (Extended Data Fig. 7i, j), which we have now added. This supports the robustness of hbRG-associated transcriptional programs across datasets and species.

Together, these results demonstrate that both chromatin accessibility and transcriptional differences between human and chimpanzee are highly reproducible across independent datasets generated from different cell lines, experimental systems, and developmental stages. We have incorporated these analyses into the revised manuscript (Extended Data Fig. 4, 5, 7 and 8 and Methods).

2. The description of the cell sorting method lacks clarity. It seems that some cell types (DL-N and bIPs) were isolated from one set of organoids (referred to as "unlabeled tissue" on line 616), while others (aRG and bRG) were obtained from a separate set of 5-10 organoids ("labeled tissue," as noted on line 614). However, this distinction is not explicitly stated in the Methods section “Fluorescence activated cell sorting”. As currently written, it could also be interpreted that all four cell types were derived from the same set of organoids and sorted into three tubes each (3 tubes × 4 cell types = 12 tubes). This clarification is important, as it directly affects the interpretability and comparability of downstream analyses. If the first scenario is correct, it raises the question of whether DL-N and bIPs can be reliably compared to aRG and bRG, given that they originate from different organoids. Ideally, all sorted cells should be derived from the same set of organoids and pooled into a homogeneous suspension before being split into two aliquots (one for bulk ATAC-seq and the other for bulk RNA-seq) to ensure consistency and facilitate meaningful comparisons across assays.

Neurons and bIPs, as well as aRG and bRG, were indeed isolated from two separate organoid pools (unlabelled and DiI-labelled, respectively), as DiI-labelling has to be performed on intact tissue to reveal basal contacts. Importantly, however, these pools were randomly sampled from the same differentiation batch and were processed in parallel under identical culture conditions. The separation into labelled and unlabelled pools was necessary to enable DiI-based identification of radial glia subtypes, but does not reflect independent biological samples.

We agree that a detailed description of the approach is important and have now clarified this explicitly in the Methods section. Because all cell populations originate from the same batch and were handled simultaneously, we do not expect batch effects or labelling-related biases to confound comparisons between cell types. Furthermore, we observe consistent patterns across independent experiments, cell lines and published data sets (see responses to reviewer 1, point 1 and this reviewer, point 1), supporting the robustness of the comparisons.

3. The authors selected an 8-week (or 54 days) time point for in vitro organoid analysis and compared this stage specifically to human prenatal development at GW13/14. However, the rationale for choosing this specific time point remains unclear and the manuscript does not adequately account for interspecies differences in developmental timing. Given that neocortical development in humans is more protracted than in great apes, it cannot be assumed that

organoids from chimpanzees, gorillas, and humans cultured for the same duration are developmentally comparable. The authors should carefully determine which in vitro developmental stage in each great ape species best corresponds to human cortical development in vivo, and compare the developmental maturation of organoids across species. They should clarify the rationale behind their time point selection and provide supporting evidence, such as transcriptomic age estimation or stage-specific marker expression, to justify developmental comparisons across species. Additionally, while the use of iPSC-derived organoids from multiple primates is a strength, the Discussion should more systematically address the limitations of organoids as developmental models, including key differences from in vivo development and how interspecies differences in developmental timing may affect interpretation.

We agree that the time point of analysis and interspecies differences in timing are key to our study. We selected week 8 of CeO development and gestational weeks (GW) 13–14 of human foetal cortex, as all four cell types of interest are present at these stages (Fig. 1c). We now dedicate a paragraph in the results section to explain our reasoning (see manuscript lines 107–122). First, we refer to life history data for gorillas, chimpanzees and humans (see new Extended Data Fig. 1d), which indicate that gestation length and developmental milestones are fairly comparable across the species. Second, we summarise published transcriptome data from cerebral organoids across the three species (see new Extended Data Fig. 1e), indicating that the mid-neurogenic stage (1–3 months of organoid development) is well aligned between gorillas/chimpanzees and humans (unlike early neurodevelopment and later neuronal maturation). Third, we provide immunohistochemical data for our gorilla, chimpanzee and human CeOs at 6, 7 and 8 weeks of organoid development (see new Extended Data Fig. 1f), which are also well aligned. In sum, this suggests that 8 weeks of organoid development is a valid time point for comparing bRG transcriptome and open chromatin data across the three species.

Finally, we have added a paragraph to the discussion section on the strengths and limitations of organoid systems (see manuscript lines 462–477).

4. The rationale for selecting FAM107A and CNGA3 genes from the 599 candidate targets is unclear. The authors should explicitly state the filtering criteria (e.g., fold change threshold, evolutionary specificity, prior functional relevance) in the Methods or Results section. Since the data presented in Fig. 4i–4n and Extended Fig. 6 are not fully convincing, it would be beneficial to validate whether the FAM107A and CNGA3 knockouts are functionally effective at the RNA and/or protein level, using RT-PCR, RNA-scope, or immunocytochemistry, particularly since the CNGA3 antibody appears to be working in Fig. 4j. Moreover, morphological analyses intended to support strong conclusions should be based on 3D reconstructions (quantitative morphometric analyses, such as total neurite length, number and order of branches, Sholl analysis, basal process thickness/terminal counts); without them, the interpretations remain largely speculative. It is also important that the control and both

knockout organoids, along with zoomed-in images of GFP^{+} cells, be presented side by side for proper comparison.

We now provide specific information on the criteria used to arrive at the candidate genes in the results section (see manuscript lines 315–320).

The knockout strategy for *FAM107A* and *CNGA3* was validated using three complementary approaches. First, the gRNA cutting efficiency was evaluated *in vitro* using a PCR template (Extended Data Fig. 14b, g). Second, the deletion in cells was confirmed in the iPSC line that was used to generate human organoids (Extended Data Fig. 14c, h). Third, in the revised manuscript, knockout was confirmed by immunohistochemistry in electroporated cells of CRISPR/Cas9-targeted human organoids (see new Extended Data Fig. 14d, e, i, j), showing reduced protein levels of FAM107A and CNGA3.

More detailed morphological analysis of SOX2+ EOMES– bRG in the subventricular zone of hCOs is now provided in the revised manuscript, including quantification of the number of primary and all processes, branching index, soma size, process length, and number of intersections in Sholl analysis (see new Fig. 5d, e and Extended Data Fig. 15e–i). Knockout of *FAM107A* significantly reduced both the number of primary processes and the total number of processes, without affecting process length, branching, or other parameters, while *CNGA3* knockout did not significantly alter any of the measured process parameters. Representative images are now shown for control and KO organoids (see new Fig. 5d).

Minor points:

1. The authors only present a brightfield image of a whole gorilla cerebral organoid at week 8 (Fig. 1b). From the figure, it appears that the authors are working with organoids cultured as whole spheres (unsliced) rather than sliced organoids. Please clarify this point. In addition, could the authors provide corresponding images of chimpanzee and human organoids for comparison?

We have clarified in the main text and methods that the cerebral organoids are cultured as whole spheres. In addition to the human cerebral organoids (CeO), we added human sliced cortical organoids (CO) in the revised manuscript (new Extended Data Fig. 4) for further support. Additionally, as requested, we have added images of cerebral organoids from all three species (new Fig. 1b).

2. Please provide FACS plots for organoids from all three species, not just the GW13/14 human fetal cortex sample. This is important for comparative analysis and to assess consistency across models.

We have added FACS plots for organoids from all three species (see Extended Data Fig. 2a–c) to facilitate comparison of the sorts. Moreover, we have added RT-qPCR data of marker

genes for cerebral organoids from all three species and all four cell types to validate the sorted populations (see new Extended Data Fig. 2d–f).

3. The authors should maintain consistency by using official gene symbols rather than aliases, for example, using EOMES instead of TBR2, or BCL11b instead of CTIP2.

The official gene symbols have now consistently been used throughout the manuscript.

4. Staining with only the dye DiI and the markers EOMES and SOX2 do not provide sufficiently convincing evidence for the presence of basal radial glia (bRG) in the cerebral organoids. To support this claim, additional immunostaining is needed using bRG-specific markers (such as HOPX), as well as proliferation markers (such as pVIM), given that bRG divide within the outer subventricular zone, a characteristic feature of bRG in vivo. This analysis should be performed across all three organoid types from each species. In addition, the authors should provide channel-split images in Fig. 4i–4n to confirm that these bRG cells are indeed EOMES- but SOX2+, as claimed.

As requested by the reviewer, we have performed staining for HOPX and pVIM to support the morphology-based (DiI) characterisation of bRG, which had revealed the presence of previously described bRG morphotypes with a single basal process, an apical and basal process and a bifurcated basal process (see Fig. 1d and Extended Data Fig. 1b) (Betizeau et al. 2013; Kalebic et al. 2019; Ossola et al. 2025). We now show images of SOX2+ EOMES– HOPX+ bRG with a pVIM+ basal process in the subventricular zone of CeO from all three species (see new Extended Data Fig. 1c), further supporting that CeOs can be used for functional investigations of bRG. Additionally, we now provide split images for the CRISPR/Cas9-mediated KO of *FAM107A* and *CNGA3* (see new Fig. 5d), which shows examples of electroporated GFP+ SOX2+ EMOES– bRG in the subventricular zone of human organoids.

5. Please clarify whether the RT-qPCR used to confirm the identity of the sorted cell populations was performed on RNA extracted from the same sorted cells that were used for ATAC-seq or RNA-seq. If not, please explain the rationale for using a separate sorted tube/ sample.

The same cell suspension was used to sort samples for ATAC-seq, RNA-seq and RT-qPCR, resulting in matched samples. Using this strategy, RT-qPCR analysis of marker genes served as a quality control step for the isolation of specific cell populations by FACS before proceeding to transcriptome and chromatin analysis. This has been further clarified in the revised methods (see manuscript lines 634–636).

6. As stated in line 126, the authors "leveraged cell type-specific Hi-C data of cortical organoids to link our candidate regions to their target genes." While this is a valid approach, in the context of identifying human-specific regulatory elements, it would be more appropriate to incorporate

or at least consider publicly available Hi-C datasets from human fetal brains and in vitro neural progenitor cells (NPCs) of both human and chimpanzee origin (e.g., Keough et al., 2023; Pal et al., 2025; Won et al., 2019). Without direct comparative analyses using both human and chimpanzee Hi-C data, identification of hbRG cCRE activity should be made with caution. The current approach does not fully exclude the possibility that similar regulatory interactions may also be present in non-human primates.

We would like to clarify that our use of Hi-C data is not intended to identify human-specific chromatin interactions, but rather to assign putative target genes to cCREs that show increased accessibility in human compared to non-human primates. We agree that many regulatory interactions are likely conserved across species, and our conclusions therefore focus on species differences in cCRE activity within largely shared regulatory landscapes, rather than on the presence or absence of specific chromatin contacts. The use of Hi-C is described in the manuscript (see Results lines 175–177, Figure legends lines 1277–1279 and Methods lines 761–766). The possibility of human-specific rewiring in the context of morphoregulatory gene regulatory networks is an interesting avenue for future investigation.

7. Since the study primarily relies on organoid/fetal brain Hi-C data to pair bRG-specific cCREs with target genes, the authors should strengthen the evidence chain by integrating “accessibility–expression” correlation analyses across samples and cell types (bulk, single-cell, or sorted populations). This would reduce false positives from purely proximity-based assignments and improve confidence in distal enhancer–target links.

To provide orthogonal support for distal cCRE–target gene assignments inferred from human organoid Hi-C data, we assessed accessibility–expression correlations across matched cell populations. We implemented two complementary approaches. First, we computed Spearman correlations for individual cCRE–gene pairs (“pairwise analysis”), comparing accessibility of each distal element with expression of its linked gene across matched groups. Second, we performed an aggregated analysis in which, for each cCRE, accessibility was correlated with the mean expression of all linked genes, capturing coordinated regulatory effects at the level of gene sets.

In human samples, Hi-C-linked pairs showed a modest but consistent increase in accessibility–expression concordance compared to random gene assignments (pairwise: median $\rho = 0.024$ vs 0.012, Fig. 1A below). The aggregated analysis showed a substantially stronger shift (median $\rho = 0.085$ vs 0.009; fraction of positive correlations 0.572 vs 0.506, Fig. 1B below), consistent with averaging reducing noise and capturing shared regulatory programs across linked genes. Notably, when restricting the analysis to a curated set of 599 bRG-associated cCREs and their Hi-C-assigned target genes, accessibility–expression concordance was further increased (median $\rho = 0.079$ vs 0.010; fraction positive 0.574 vs 0.515, Fig. 1C below), indicating stronger functional coupling within this biologically relevant subset. Given the large number of tested pairs in the genome-wide analysis, statistical significance was not used as a

primary metric; instead, we focused on effect size, which indicated a reproducible but moderate enrichment of concordant behaviour for linked pairs.

Together, these analyses provide supporting, population-level evidence for the biological relevance of the inferred enhancer–target links, while also indicating that effect sizes per individual pair remain modest. While these analyses provide orthogonal support for the inferred enhancer–target links, the limited number of matched samples (and corresponding low number of independent observations for correlation analysis) constrains the resolution of these tests. We therefore present these results in the response to reviewers.

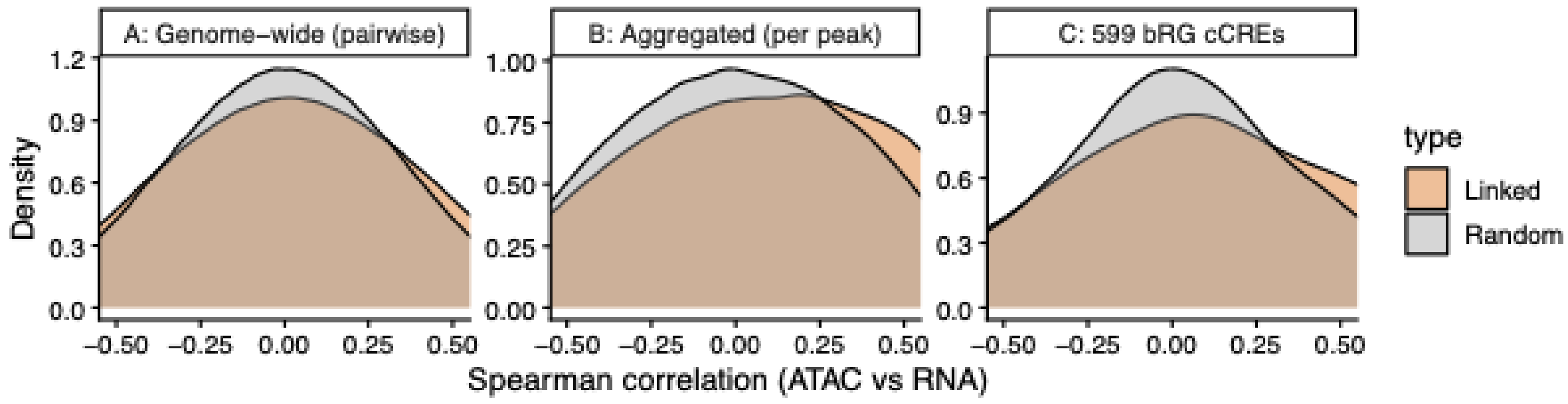

Figure 1. Accessibility–expression correlation analysis supports Hi-C-derived enhancer–target links. A, Genome-wide pairwise correlations between distal cCRE accessibility and expression of linked genes show a modest shift towards higher correlation compared to random gene assignments. **B**, Aggregating expression across all genes linked to each cCRE results in a stronger shift, consistent with reduced noise and coordinated regulation. **C**, Restricting the analysis to our 599 hbRG-cCREs and their Hi-C-assigned target genes reveals a more pronounced increase in correlation, indicating stronger functional coupling in this biologically relevant subset.

8. What was the rationale for excluding gorilla organoids from the RNA-seq analyses in Fig. 3? It is unclear whether some of the 939 identified DEGs are also expressed in bRG sorted from gorilla CeOs. The authors may have overstated their conclusion in line 148 by referring to a ”human bRG-specific phenotype”. It would also be beneficial to include gorilla datasets alongside chimpanzee data in all other analyses. In addition to the previous comment, a log2FC > 1 threshold is relatively low. Please consider using a more stringent criteria, such as log2FC > 1.5 or higher.

This is an important point. We have now added RNA-seq data from gorilla CeOs to the revised manuscript (see new Fig. 4a, b and Extended Data Fig. 7 and 12g) to strengthen the findings of human-specific gene expression (see manuscript lines 224–230). Genes upregulated in human compared to chimpanzee bRG showed generally lower expression in gorilla compared to human bRG (Extended Data Fig. 7i, j), suggesting human up- rather than chimpanzee downregulation. This was further supported by analysis of published scRNA-seq data from foetal macaque (responses to Reviewers 1 and 2, point 1).

We have considered different thresholds for the RNA-seq analysis. We reasoned that the expected differences between so closely related species would be small. Indeed, if we use a log2FC > 1.5, the list of differentially expressed genes reduces by roughly half, and no

significant GO terms remain. Therefore, we have kept the log2FC > 1 threshold. Ultimately, novel traits, such as increased morphology of bRG, may arise from many small changes in gene regulatory networks, rather than from a few mutations of large effect (Pollen et al. 2023).

9. The study focuses on human-increased bRG cCRE targets, but whether FAM107A and CNGA3 have conserved or divergent functions in non-human primates has not been tested. Parallel perturbations in chimpanzees or gorilla organoids would strengthen evolutionary specificity claims.

We thank the reviewer for this suggestion. To address this point, we have overexpressed *FAM107A* in chimpanzee CeO (see new Extended Data Fig. 16). As discussed in response to reviewer 1 (point 5), the phenotype of the overexpression was similar to the knockout in human CeO, suggesting that *FAM107A* expression levels are tightly regulated by gene regulatory networks in the human developing neocortex to achieve appropriate expression levels. This is in line with previous work on the morphoregulatory protein ZNF804A, which showed that both lower and higher levels affected cell morphology in the same direction (Dong et al., 2021) (see manuscript lines 415–420).

10. Currently, only reporter assays are performed. To establish causality ("enhancer variant → target gene expression → morphological phenotype"), the authors should consider CRISPRi/CRISPRa on CREs. Parallel perturbations in aRG, bIP, and different developmental stages would demonstrate effect specificity to bRG and critical time windows. It is also important to confirm that perturbations do not alter bRG identity markers (SOX2, TBR2, ZO1), to rule out fate-switch artifacts.

We have addressed this point using a dCas9-KRAB-MeCP2 CRISPRi approach (see new Extended Data Fig. 13c–f). We have designed gRNAs targeting the *FAM107A* and *GNCA3* cCREs. The constructs were electroporated into human organoids, and three days later, we sorted the cells for gene expression analysis by RT-qPCR. We expected the effect to be small, given the high redundancy of mammalian developmentally important enhancers (Kvon et al. 2021), and therefore analysed 6–10 samples per genotype. We found that the CRISPRi approach led to a small, albeit statistically insignificant, reduction in *FAM107A* expression in PAX6+ radial glia, suggesting that the cCRE may have a small contribution to *FAM107A* expression in human cortical development, but that additionally active enhancers likely contribute to *FAM107A* expression. Likewise, dCas9-KRAB CRISPRi targeting of the *CNGA3* cCRE led to a small, statistically insignificant, reduction in gene expression (see manuscript lines 357–365).

Reviewer #3 (Remarks to the Author):

It is already known that bRG plays a significant role in the evolutionary expansion of brain volume and the complex folding of the cerebral cortex due to its explosive neuron-producing

capacity. The attempt to clarify species-specific differences between humans and great apes, particularly in bRG, is a meaningful endeavor. Given the difficulty of analyzing great ape fetuses, the use of organoids with the selected species combination is somewhat justifiable. The study found that a characteristic feature of the bRG expression profile in humans is the high expression level of a gene set involved in cell morphology regulation. Furthermore, knocking out two representative genes from this set in human organoids simplified the cell morphology, suggesting that human bRG has a more complex cellular morphology than that of other species.

While this manuscript has several minor points for improvement, its core argument on human-specificity of bRG is well-understood, and the work holds some value within the research community. However, a significant weakness is that the paper's evaluation of human-specific bRG features is limited to cell morphology. While a previous study Kalebic et al Cell Stem Cell 2019 has suggested a link between cell morphology and the stemness and neurogenic capacity of bRG, this research should have directly demonstrated that the observed morphological complexity leads to increased proliferation and neuron production. It's also disappointing that the majority of the gene expression comparative analysis relies on inter-species comparisons of organoid samples, even though the authors did acquire and analyze human fetal data. They should have at least confirmed the human-specific gene expression they found by comparing human and non-human primate fetal samples, as data for primates like macaques are publicly available.

We thank the reviewer for their evaluation of our study and for recognising the value of investigating interspecies differences in great ape cortical development.

We agree that organoid models have important limitations compared to foetal tissue (see revised Discussion, lines 462–477). At the same time, great apes are highly protected species for which foetal material is not available for reserach. Great ape cerebral organoids therefore provide a unique opportunity to gain insights into the corticogenesis of our closest living relatives.

We thank the reviewer for pointing us to publicly available data on foetal macaques, to which we have compared our expression data in the revised manuscript to strengthen human-specific aspects. Specifically, we have analysed scRNA-seq from macaque foetal cortex (Micali et al., 2023) to support our conclusions (see new Extended Data Fig. 10 and 11). The results obtained from this analysis are described in the revised manuscript (see lines 277–284).

Overall, a major strength of this study is its clear and simple structure. However, the depth of the individual analyses is lacking, and the fundamental finding—the human-specific regulation of bRG cell morphology—has limited importance in a broader context. For these reasons, the manuscript is not a good fit for a journal with a wide readership, such as Nature Communications. The following are more specific peer review comments.

In response to this reviewer's comments, we have added substantial additional data and analyses to our revised manuscript. We hope that the new figures strengthen this reviewer's assessment of our findings. The specific points are detailed below.

Major concerns;

1) (Fig1d) Is the DiI labeling method, where it's placed on the organoid surface, even valid in the organoido lacking meninges and other tissues/cell types contained in the in vivo fetal brain?

For mouse embryonic and human foetal brain tissue, DiI is applied after removal of the meninges, allowing radial glia with basal processes to be labelled and isolated (Florio et al. 2015; Albert et al. 2017). The presence of bRG has now been reported in many different human neural organoid protocols (reviewed in Don and Kalebic, 2022). Here, we show that bRG (and aRG) basal processes can extend to the basal surface of CeOs, as highlighted by application of DiI to the basal surface of organoids (see Fig. 1d and Extended Data Fig. 1b).

2) (Fig3c) Instead of just bulk RNA-seq, scRNA-seq data should also be used to examine the expression dynamics of these genes in more detail. Specifically, the authors should describe the cell type specificity, expression dynamics along the cell differentiation axis, and detailed expression patterns by distinguishing between different cell types and developmental stages. The datasets of some previous studies (https://pubmed.ncbi.nlm.nih.gov/37824652/ etc) will provide additional value on the authors' study.

We thank the reviewers for their suggestion and have mined several published scRNA-seq datasets from different time points and species (mouse, macaque, chimpanzee and human), including the one suggested by this reviewer (Micali et al., 2023). For details, see also the responses to Reviewer 1 (points 1 and 2) and to Reviewer 2 (point 1).

Specifically, in the macaque foetal cortex, the human bRG upregulated gene set was primarily expressed in neural progenitor cells and excitatory neurons (Extended Data Fig. 10e), with enrichment in bRG-like populations (Extended Data Fig. 10f), confirming the pattern we have described in our dataset (Fig. 3 and Extended Data Fig. 10a).

Moreover, the morphoregulatory genes *BCL11A*, *ENC1*, *BAIAP2* and *PTPRM* showed strong expression in human CeO and foetal bRG (Extended Data Fig. 7j, 8a and 11a) but low or absent expression in gorilla and chimpanzee CeO (Extended Data Fig. 7j and 8a) as well as in macaque bRG (Extended Data Fig. 11b, c), supporting human-specific upregulation.

Mining published human snRNA-seq data across different stages confirmed consistent expression of the human bRG upregulated gene set in radial glia as well as excitatory and inhibitory neurons (Extended Data Fig. 10b–d). The results from the analysis of the published dataset are described in lines 264–286 in the revised manuscript.

3) (Fig3f) The concept of a "morphoregulatory pathway score" is questionable. More detailed explanation of the gene set is necessary. What GO terms are selected to define them?

Thank you for raising this point. We realise that the information provided was very limited and have expanded this in the revised manuscript (see manuscript lines 238–252). Moreover, we performed additional analysis to validate the pathway score (see new Extended Data Fig. 9).

Specifically, to validate the score in a cortical context, we applied the same framework to GO terms associated with apical polarity ('Apical junction assembly', 'Establishment of epithelial cell apical basal polarity' and 'Regulation of adherens junction organization'), which resulted in the expected high pathway activity in aRG compared to bRG, bIPs and neurons. Furthermore, in a published dataset of astrocyte maturation in human organoids (Verkerke et al., 2024), the morphoregulatory pathway activity score increased with cellular maturation and complexity.

4) (Fig3) If a gene expression network analysis were conducted, could common upstream transcription factors for FAM107A and CNGA3 be found?

To investigate whether *FAM107A* and *CNGA3* may be regulated by common upstream transcription factors, we performed motif enrichment analysis on their associated distal cis-regulatory elements overlapping peaks in human foetal bRG. Using a curated set of vertebrate transcription factor motifs (JASPAR2020) and comparing each set of gene-linked distal elements to a background of all distal elements overlapping across cell types in our dataset, we did not detect any transcription factor motifs that were significantly enriched for either gene after multiple testing correction. Consistent with this, no shared enriched motifs were identified between the two gene-associated regulatory element sets, suggesting that their regulation is unlikely to be driven by a common upstream transcription factor acting through a strongly enriched motif.

5) (Fig4d) The reporter assay results in N2A cells do show that species differences in the CRE sequence correlate with differences in expression induction activity. But can this be said for bRG as well? Given that there's an experimental system for overexpression in human and chimpanzee organoids, shouldn't this experiment be conducted in organoids, with a stronger focus on bRG expression?

In response to the reviewer's suggestion, we cloned the candidate regions into a plasmid containing a minimal promoter upstream of a fluorescent reporter and electroporated the constructs into hCOs (new Fig. 4i–k). The human sequences of the *FAM107A* and *CNGA3*-linked cCREs drove significant reporter gene expression in SOX2+ radial glia in hCOs. In comparison, the corresponding chimpanzee sequences showed a small, statistically insignificant, reduction in reporter gene expression. Mutation of the central human-specific

transcription factor binding site in the *CNGA3* cCRE led to a significant reduction in reporter gene expression. These results confirm that the two human hbRG-linked cCREs have the potential to enhance gene expression in human radial glia in hCO (see manuscript lines 335–345).

6) (Fig4j-k) Immunohistochemical data for FAM107A is required as well, in addition to CNGA3.

As requested by the reviewer, we performed immunohistochemical analysis of FAM107A in gorilla, chimpanzee and human CeO (see new Fig. 4l–m). In line with RNA expression levels, FAM107 protein appeared expressed at higher levels in human compared to gorilla and chimpanzee CeOs (see manuscript lines 356–357).

7) (Fig4j-k) Immunohistochemical staining for CNGA3 and FAM107A should be performed in fetal brains as well. It's impossible to determine whether the images shown are truly of bRG and not aRG, by showing the exact location of the imaged cells within SVZ-like domain in the organoids.

In addition to immunohistochemical analysis of FAM107A and CNGA3 in organoids, we also performed staining of human foetal cortex (see new Extended Data Fig. 13g). An image of the entire cortical wall is shown, including co-staining for SOX2 and EOMES, showing SOX2+ cells in the outer subventricular zone expressing FAM107A as well we sparse expression of CNGA3, as seen in the organoids.

8) (Fig4j-k) Is there a correlation between the expression levels of CNGA3 (and/or FAM107A) and cell morphology in fetal brains and brain organoids? Maybe it is useful to co-stain DiI (if it's a valid method for labeling bRG in organoids) and CNGA3 protein to demonstrate the correlation of CNGA3 level and morphological complexity.

To address this, we have also performed immunohistochemical analysis of CNGA3 in human foetal cortex (see new Extended Data Fig. 13h). Interestingly, CNGA3 is expressed in some, but not all, radial glia. There was, however, no direct correlation between CNGA3 expression and the morphology of SOX2+ HOPX+ cells in the subventricular zone.

9) (Fig4m-n) The LOF analysis of FAM107A and CNGA3 should include not only the observation of cell morphology but also assays to examine markers of cell proliferation and the number of neurons produced.

As suggested by the reviewer, we have performed immunohistochemical analysis of the proliferation marker KI67 and the early neuronal marker HuCD in human organoids electroporated with CRISPR/Cas9 ribonuclein complexes targeting *FAM107A* and *GNCA3*. The results are described in manuscript lines 406–413 and new Extended Data Figure 15j, k.

In line with previous work in the macaque foetal cortex (Betizeau et al. 2013) and in mouse embryonic cortex (Kalebic et al. 2019), we found that knockout of *FAM107A* in hCO led to a significant decrease in the number of HuCD+ KI67– neurons in the subventricular zone/cortical plate-like region of hCO, suggesting that FAM107A contributes to the morphological complexity of human bRG and its developmental consequences.

10) (Fig4m-n) In addition to LOF analysis, a GOF analysis in chimpanzee organoids should be included.

As also discussed in response to reviewer 1 (point 5) and reviewer 2 (point 9), we overexpressed human *FAM107A* in chimpanzee CeO (see new Extended Data Fig. 16). The phenotype was similar to the KO in human CeO, leading to a (slightly) reduced morphological complexity. This suggests that *FAM107A* expression levels are tightly regulated in the human developing neocortex. In line with this, previous work on the morphoregulatory protein ZNF804A has shown that both lower and higher expression levels affected cell morphology in the same direction (Dong et al., 2021) (see manuscript lines 415–420).

11) (Fig4m-n) The analysis should distinguish and evaluate not only bRG but also aRG cell types. Considering that these genes are also expressed at high levels in aRG, it can be speculated that they have some function. Do aRG also have morphological diversity that is regulated by these genes?

Indeed, this is an interesting question. While both *FAM107A* and *CNGA3* are also expressed in aRG, knockout did not affect aRG bipolar morphology (see new Extended Data Fig. 15c, d), indicating bRG-specific effects on morphology. This has been added to the revised manuscript (see lines 395–397).

12) (Fig4m-n) The current data provides no evidence to suggest that a selective analysis of bRG was performed. Shouldn't DiI labeling be done here as well?

In the morphology analysis, we have included SOX+ EOMES– cells in the subventricular zone (see new Fig. 5b, d), in line with previous literature suggesting that the human subventricular zone contains proliferative progenitors with radial glia properties (Hansen et al. 2010; Fietz et al. 2010), characterised by the expression of SOX2, along with the absence of EOMES (Pollen et al, 2015; Qian et al, 2016, 2020). Sparse DiI labelling would be needed to delineate the processes of individual cells, and that would therefore provide only a minimal overlap with electroporated cells. Instead, for the KO analysis, we have used GFP to track processes.

POINT-BY-POINT RESPONSE TO REVIEWERS

Reviewer #1 (Remarks to the Author):

The authors have addressed all of my main concerns.

Reviewer #2 (Remarks to the Author):

The authors have addressed all of my concerns as well as those raised by the other reviewers. Their responses are thorough and the revision has been carried out with great care.

We are pleased to read that reviewers 1 and 2 are fully satisfied with the revised manuscript, and we thank them again for their input in further improving our study.

Reviewer #3 (Remarks to the Author):

General Comments:
The authors have significantly expanded the dataset in this revised manuscript, and it is evident that they have made a sincere and comprehensive effort to address all the concerns raised in the initial review. The increased volume of data has substantially strengthened the foundation of their claims.
In particular, the ATAC-seq and gene expression analyses have been greatly enriched. The original data presented here for the first time are intrinsically valuable to the research community. Furthermore, the integrated comparative analysis with other public datasets has further enhanced the significance of their original findings. Regarding the omics data, the data representation is excellent and exceptionally clear to the reader.

We appreciate the feedback from Reviewer 3, who acknowledges the significant expansion of the data analysis and additional experiments in the revised manuscript. We would like to thank the reviewer for their suggestions to consolidate our manuscript. In the following, we address the remaining two open points.

Specific Comments:
1. Functional Analysis of FAM107A and CNGA3 (Figure 5 and Extended Data Figure 15)
The authors have performed an impressive set of additional experiments quantifying bRG cell proliferation and neuronal differentiation in response to my previous request. While I highly commend the effort to include these analyses, I must candidly state that the results are somewhat disappointing in light of the authors' primary hypothesis.
The authors conclude that FAM107A knockout leads to simplified cell morphology and a concomitant decrease in the proportion of differentiated neurons (Lines 406-413), suggesting a link between morphological complexity and developmental consequences. However, an objective assessment of the data in Extended Data Fig. 15j and k conveys the opposite

impression: the loss of FAM107A appears to have a negligible impact on the core stem cell programs, such as bRG proliferation or differentiation.

From the initial submission, my view has been that the theme of "morphological control in bRG" holds universal scientific value only if such control is shown to be essential for bRG stemness. Despite the massive addition of data, this fundamental point remains unproven. In fact, the results in Extended Data Fig. 15j and k seem to support the inverse conclusion—that bRG morphological complexity may not be as critical to the stem cell program as the authors claim.

The concept of morphological control in bRG is supported by several previous studies, as discussed in the manuscript (lines 451–460), including by Betizeau, Dehay, *Neuron* 2013 and Kalebic, Huttner, *Cell Stem Cell* 2019. Our unbiased transcriptomic and epigenomic approach identified human-specific morphoregulatory signatures as a characteristic of neocortex evolution, extending this concept to the comparison of three great ape species with an evolutionary distance of only 6-10 million years (compared with mouse and ferret, which are separated from us by more than 80-90 million years of evolution). Most of the gene expression differences described in our study are likely to originate from noncoding changes in *cis*-regulatory elements, reflected in differential chromatin accessibility at several hundred putative enhancer regions. Many adaptive sequence changes in noncoding regions are likely to have small effect sizes, making them hard to detect experimentally (Fair, Pollen, *Curr Opin Neurobiol* 2023; Song, Walsh, *Nat Neuroscience* 2026). A specific trait may involve a small number of genomic changes with large effects, or many genomic changes with small effects (Song, Walsh, *Nat Neuroscience* 2026). For instance, previous studies by us and others have identified a limited number of human-specific genes with large effects that regulate bRG proliferation and neocortex size (see the introduction, lines 74–75, and the discussion, lines 489–490). On the other hand, here, we identified several hundred genomic regions with interspecies differences in the activity of *cis*-regulatory elements, influencing gene expression programs linked to cell morphology. For two genes (*FAM107A* and *CNGA3*), we show that their individual knockouts result in significant changes in bRG morphology. Given that, in addition to *FAM107A* and *CNGA3*, we detected several additional genes linked to the regulation of cell growth and cell projections (see Fig. 3e), one might expect these genes to act in concert to shape bRG morphology, stemness, proliferation, and neuronal output. As discussed in our manuscript (lines 406–413), knockout of *FAM107A* in human cortical organoids led to a significant decrease in the number of neurons, whereas there was no measurable effect on proliferative progenitors. Importantly, knockout and overexpression equally affected bRG morphology, highlighting the importance of appropriate gene expression levels and underscoring the importance of gene regulatory elements. Over the course of human neocortex development, during which neurogenesis lasts several months, even small effects on the core stemness network and neuronal output can add up to meaningful differences in neuronal number and neocortex size. We have expanded the discussion section to include differences in effect size (lines 450–452 and 484–493).

2. Validation of FAM107A Knockout (Extended Data Figure 14)

There are also concerns regarding the technical validation of the FAM107A knockout. In the provided images, the intensity of the immunostaining signal shows almost no detectable decrease, which raises doubts as to whether the gene function was effectively abolished.

While it is helpful that the authors illustrated the exon structure and guide RNA (gRNA) targets in Extended Data Figure 14, they must provide evidence showing the actual impact at the protein level. Specifically, will the designed gRNA result in a complete null? Additionally, the authors should clarify the position of the antibody epitope relative to the gRNA target site to ensure that the observed signal is not from a truncated or partially functional protein.

The reduction in FAM107A protein levels after CRISPR/Cas9-targeting in electroporated human organoids is shown in Extended Data Figure 14. We acknowledge that, due to electroporation-based targeting at the individual-cell level, it was not easy to appreciate reduced protein levels. Therefore, in addition to the quantification, which shows a significant reduction in FAM107A protein (Extended Data Fig. 14f), we now also provide zoomed images of 7–9 individual cells per condition in the VZ and SVZ, respectively (new Extended Data Fig. 14e). These zoomed images supplement the quantification and visually show the reduction in FAM107A protein levels in GFP+ electroporated cells.

The antibody used to detect human FAM107A represents a rabbit polyclonal antibody, for which the entire protein was used as an immunogen. FAM107A represents a small protein of 144 aa (17 kDa). Even though we cannot rule out the possibility that a truncated protein was generated in some of the targeted cells, overall, we detected a significant reduction in protein levels (Extended Data Fig. 14f).

POINT-BY-POINT RESPONSE TO REVIEWERS

Reviewer #3 (Remarks to the Author):

I believe the authors have fully addressed all of my concerns, which has significantly enhanced the credibility and robustness of the manuscript. Thanks to their extensive efforts, this paper now makes a highly valuable contribution to the field.

We are pleased to read that reviewer 3 is fully satisfied with the revised manuscript, and we thank them for their input on our study.
